# Peptide-based therapeutics targeting GPCRs: recent applications in the treatment of metabolic disorders

**DOI:** 10.3389/fphar.2026.1930768

**Published:** 2026-09-16

**Authors:** Simona Di Martino, Maria De Rosa

**Affiliations:** Ri.MED Foundation, Palermo, Italy

**Keywords:** conjugation, diabetes, fatty acid, glucagon, G-protein coupled receptor, hormone, obesity

## Abstract

G protein-coupled receptors are among the largest protein superfamilies in the human genome and are responsible for sensing diverse extracellular signals and mediating them into cellular responses. Owing to their broad range of subunits and downstream effectors regulated by specific ligands, these receptors have been appealing pharmacological targets for the discovery of new drugs. Peptide agonists emerged as compelling treatment options for metabolic disorders, and many are currently used in therapy. This perspective comprehensively summarizes the medicinal chemistry efforts over the past 5 years toward the discovery of novel agonists targeting G protein-coupled receptors as promising, life-changing peptide therapies to treat metabolic disorders, like obesity and diabetes.

## Graphical Abstract

**Figure:**
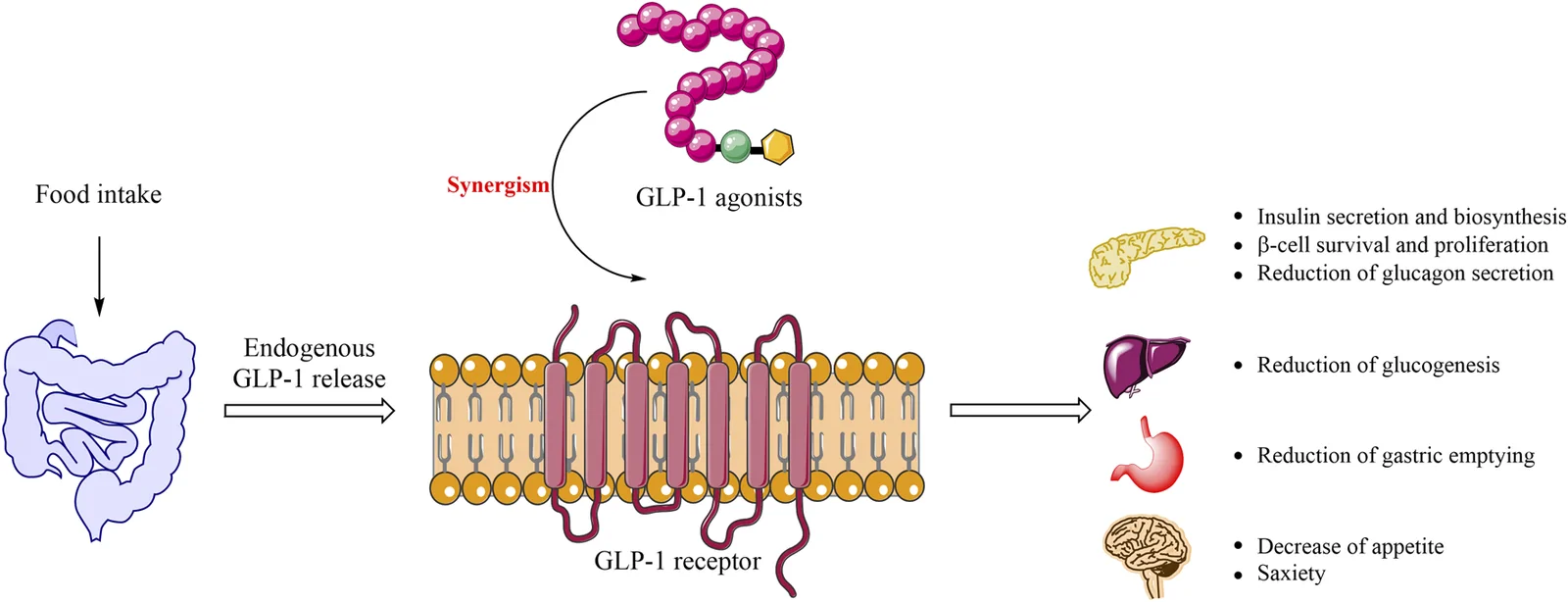

## 1 Introduction

G-Protein Coupled Receptors (GPCRs) are the largest and more diverse family of integral membrane proteins in eukaryotes, with nearly 950 genes encoded in the human genome (Kim et al., 2025). This protein family comprises a single polypeptide wrapped into a circular shape that is twisted into seven transmembrane α-helical segments embedded in the cell membrane, and, for this reason, GPCRs are also known as seven transmembrane receptors. The C-terminus is located in the intracellular environment, while the extracellular N-terminus detects a wide range of stimuli, including light (e.g., by the light-sensitive receptor rhodopsin) (Varma et al., 2019), odour (by olfactory receptors located in the olfactory epithelium) (Spehr and Munger, 2009), molecules, peptides, hormones and neurotransmitters (Rehman et al., 2026). Upon binding with the agonist, the receptor undergoes conformational changes at the intracellular side, promoting the interaction with the associated guanine nucleotide-binding (G) proteins. This reaction triggers the biochemical cascade selectively activating or inhibiting different cellular functions, ultimately eliciting physiological responses across multiple organs. GPCRs are expressed in almost all cell types of the human body, and are generally grouped into four families based on the sequence homology and functional similarity: class A (rhodopsin-like), class B (including the secretin-like B1, and adhesion B2 subtypes), class C (metabotropic glutamate/pheromone receptors), and class F/O (frizzled/smoothened receptors) (Arimont et al., 2019).

Class A represents the largest family, and is further divided in three subgroups, namely, group 1 including rhodopsin and β-adrenergic receptors with the binding site located within the transmembrane segment; group 2 containing receptors for peptides, with the binding site including the N-terminus, the extracellular loops, and the superior parts of the transmembrane segment; group 3 comprising GPCRs for glycoprotein hormones, which are characterized by large extracellular domains and binding sites (Tuteja, 2009).

Class B includes receptors for 15 different peptide hormones (e.g., glucagon, the glucagon-like peptide (GLP-1), the glucose-dependent insulinotropic polypeptide (GIP), and calcitonin) (Hollenstein et al., 2014). The agonists of this family are typically large peptides derived from endogenous ligands, like the antidiabetic drug exenatide (approved by FDA in 2005) (Bond, 2006), and the synthetic salmon calcitonin used to treat postmenopausal osteoporosis (Miacalcin, approved by FDA in 1986) (Lewiecki, 2009; Congreve et al., 2011). In fact, the large orthosteric binding sites in secretin-like GPCRs are less druggable for small molecules compared to class A, except for the deep region of the corticotropin-releasing factor receptor 1 (Congreve et al., 2011; Mizera and Latek, 2021; Dzierba et al., 2008).

Class C consists of 22 human proteins with a large extracellular N-terminus binding the endogenous ligands. Drug discovery efforts toward allosteric modulators for class C GPCRs have been highly successful, with several chemotypes identified for multiple members. Cinacalcet is a positive allosteric modulator of the calcium-sensing receptor and was one of the first GPCR allosteric ligands to enter in clinical phase (Leach and Gregory, 2017).

Class F/O comprises 11 cell surface receptors called Frizzled 1–10 and Smoothened; it is undergoing target validation studies in cancer therapy. Classes D and E are non-mammalian families, with class D including fungal mating pheromone receptors, while class E comprises cyclic adenosine monophosphate (cAMP) receptors from slime mould.

Given the wide range of G protein subunits and downstream signalling pathways together with their ability to recognize a variety of ligands (spanning classical peptide hormones and a broad spectrum of bioactive metabolites), GPCRs are appealing and versatile targets (Cho et al., 2025). For decades, they attracted tremendous interest in both the pharmaceutical industry and academia accounting today about 35% of all approved drugs (Schröer and Seifert, 2025). In general, dysregulation of GPCRs signalling is involved in the pathogenesis of several chronic diseases, such as cancer, viral infections, inflammation, neurological and metabolic disorders. Obesity, metabolic syndrome and type-2 diabetes mellitus (T2D) are three related conditions where more than thirty different GPCRs are associated to the disease development and progression (Barella et al., 2021). In this context, peptides emerged as the most successful GPCR-targeting therapeutics bridging the gap between biologics and small molecule ligands, due to their higher affinity, potency, and selectivity to the receptors (Ma et al., 2025; Xue et al., 2025; Griffith et al., 2022). Being intrinsic signalling molecules for many physiological functions, peptides offer the opportunity to closely mimic natural pathways (Lau and Dunn, 2018). Indeed, most of the existing peptide therapeutics are agonists, and derive from chemical modifications of the sequence of endogenous GPCRs ligands. However, new strategies are being employed to develop both agonists and antagonists, offering chemical novelty and improved drug-like properties. When engaging GPCRs, it is important to consider the dynamic nature of the target because the multiple active or inactive receptor conformational states influence the interaction with the peptide; each state is stabilized by a different ligand interaction determining whether a peptide works as an agonist or antagonist (Xue et al., 2025). Thus, the design of ligands requires a state-specific strategy to ensure high binding affinity combined with a selective stabilization of the receptor conformation. Notably, in the recent years, artificial intelligence-driven peptide rational design has garnered considerable interest helping to design agonist and antagonist candidates for specific receptor states (Bryant and Elofsson, 2022; Sawyer et al., 2025).

In this perspective, we overviewed the developments over the past 5 years of peptide therapeutics targeting GPCRs to treat metabolic disorders. The rational design, the synthesis, and the applications of (semi)synthetic peptides and peptide-small molecule conjugates are the focus of this work.

## 2 Peptide-based therapeutics: pros and cons

Natural bioactive peptides consist of a short chain of amino acids, typically ranging between 2 and 50 residues, and present a broad pharmacological spectrum including antimicrobial, antiplatelet, antioxidant, antihypertensive, and antitumor properties (Rodríguez-Cabello et al., 2026). Their biological function depends on the amino acid composition and structural conformation, including α-helices, β-sheets, turns, or random coils. The structural arrangements straightly modulate the overall stability, bioavailability, and target specificity. Due to their crucial role in the regulation of energy homeostasis and metabolism, peptides gained an ever growing relevance as drug candidates for clinical development (Zhu et al., 2022). In the past decades, advances of structural biology, recombinant biologics, and new synthetic technologies expanded the peptide therapeutics pipeline (e.g., the investigational MariTide by Amgen (Wu et al., 2026), (see paragraph 3.1.6.1) cotadutide by MedImmune (Ambery et al., 2018), the FDA approved tirzepatide by Eli Lilly (Coskun et al., 2018), and Novo Nordisk’s semaglutide (Lau et al., 2015), see Table 1), with over 2,000 peptides reaching the development stage, and mainly including GLP-1 receptor (GLP-1R) agonists for oncology, metabolic and rare diseases (Inside, 2026; Valencia-Rincón et al., 2025; Rajagopal et al., 2023). Compared to macromolecules, peptide drugs offer several advantages enabling rapid tissue penetration, while retaining potency and selectivity for the target, higher specificity, lower toxicity, and superior long-acting delivery properties. Moreover, peptides show a predictable metabolism, determining a low incidence of adverse effects, and low failure in clinical development; conversely, small molecule candidates often release toxic metabolites. Finally, peptides can be easily synthesized via high throughput standardized production. However, major challenges include poor oral bioavailability and rapid enzymatic degradation, with consequent short half-life, and frequent dosing (typically via parenteral administration) (Li Petri et al., 2022; Chen et al., 2022). In some rare cases, peptides can also cause immunogenic responses. Moreover, the low absorption seems mainly due to their big size and high molecular mass. Also, the lack of specific transport system determines a rapid excretion from the body. Further limitation in the use of peptide therapeutics is the lack of a robust secondary and/or tertiary structure, together with the high hydrophilic character, which contribute to make them unstable and easily denatured. Indeed, the short half-life is due to rapid renal clearance (for peptides with low molecular weight below 30–60 kDa) (Tan et al., 2021), and degradation by proteolytic enzymes (proteases/peptidases). Dipeptidyl peptidase-4 (DPP-4) is one of the main enzymes responsible for the rapid degradation and inactivation of incretin hormones, specifically GLP-1 and GIP. This ubiquitous serine protease cleaves N-terminal dipeptides with alanine or proline residues, and catalyses the removal of two N-terminal residues of GLP-1 (His-Ala) and GIP (Tyr-Ala) (Figures 1, 2) leading to a significant drop in activity. For this reason, DPP-4 represents a key modulator of GLP-1R and GIP receptor (GIPR) activity within the epithelial and endothelial cells of many tissues including the liver.

**TABLE 1:** Peptide drugs targeting GPCRs used in clinic to treat metabolic disorders.

Drug	Peptide template	Key chemical modifications^a^	GPCR	Therapeutic use	Brand name	Administration route and dosing frequency	Clinical benefits	Side effects
Salmon calcitonin	Salmon calcitonin	NA	CTR	Post-menopausal osteoporosis, Paget’s disease, hypercalcemia	Miacalcin	Subcutaneous, intramuscular, intravenous infusion (dosages adjusted based on the patient’s condition), or intranasal spray (daily)	Vertebral fracture prevention, analgesic benefits, bone turnover suppression	Nausea, facial flushing, injection site irritation, hypocalcemia, risk of various cancer
Pramlintide	h-Amylin	Ala25Pro Ser28Pro Ser29Pro	AMYR	Diabetes	Symlin	Subcutaneous (prior meal)	Slows gastric emptying, suppresses glucagon secretion, satiety	Gastrointestinal symptoms, severe hypoglycemia (in co-administration with insulin)
Exenatide	Gila monster Exendin-4	NA	GLP-1R	Diabetes	Byetta	Subcutaneous (twice daily)	Effective glycated hemoglobin reduction, modest weight loss, low risk of hypoglycemia	Gastrointestinal symptoms, hypoglycemia, pancreatitis, ischemic renal failure, gallbladder disease, risk of thyroid C-cell tumors (confirmed in rodents)
Liraglutide	GLP-1	Arg34Lys, γGlu-palmitic at Lys26	GLP-1R	Diabetes, obesity	Victoza	Subcutaneous (once daily for 1 week)	Insulin sensitivity, slow digestion, satiety, menstrual regularity, protective effect against hepatic steatosis, anti-inflammatory effect, prevention of muscle atrophy	Acute pancreatitis, gallbladder disease, kidney impairment, slightly elevated resting heart rate
Dulaglutide	GLP-1 fused to IgG4	Ala8Gly Gly22Glu Arg36Gly	GLP-1R	Diabetes	Trulicity	Subcutaneous (once weekly)	Effective glycated hemoglobin reduction, cardiovascular benefits	Gastroparesis, increased risk of acute pancreatitis, gallbladder disease, acute kidney injury, risk of thyroid C-cell tumors
Albiglutide	GLP-1 fused to hSA	Ala8Gly	GLP-1R	Diabetes	Tanzeum	Subcutaneous (once weekly)	Improved glycemic control, low hypoglycemia risk, gastrointestinal tolerability	Acute pancreatitis, gallbladder disease, risk of medullary thyroid carcinoma
Semaglutide	GLP-1	Ala8Aib Lys34Arg 2xAEEA-γGlu-C-18 fatty diacid at Lys26	GLP-1R	Diabetes, obesity	Ozempic	Subcutaneous (once weekly), oral (tablet, once daily)	Improved glycemic control, effective weight loss, satiety, cardiovascular benefits, anti-neuroinflammatory effects, menstrual regularity, renal protection	Chronic gastrointestinal issues, acute pancreatitis, acute kidney injury, nutrient malabsorption, anesthetic risks (e.g., pulmonary aspiration, residual gastric content), muscle loss, nonarthritic anterior ischemic optic neuropathy and diabetic retinopathy, Thyroid C-cell tumors Gallbladder disease
Tirzepatide	GIP	Modified with GLP-1 and extendin-4 tail Ala2Aib Ala13Aib 2xAEEA-γGlu-C-20 fatty diacid at Lys20	GLP-1R, GIPR	Diabetes, obesity	Zepbound, Mounjaro	Subcutaneous (once weekly)	Effective glycated hemoglobin reduction, substantial weight loss, cardiovascular benefits, sleep apnea treatment, kidney protection, anti-inflammatory effective menstrual regularity	Gastrointestinal symptoms Rare: Pancreatitis, gallbladder disease, kidney injury, severe stomach paralysis

^a^ Amino acid numbering is based on the sequence of the natural ligand.

**FIGURE 1:**
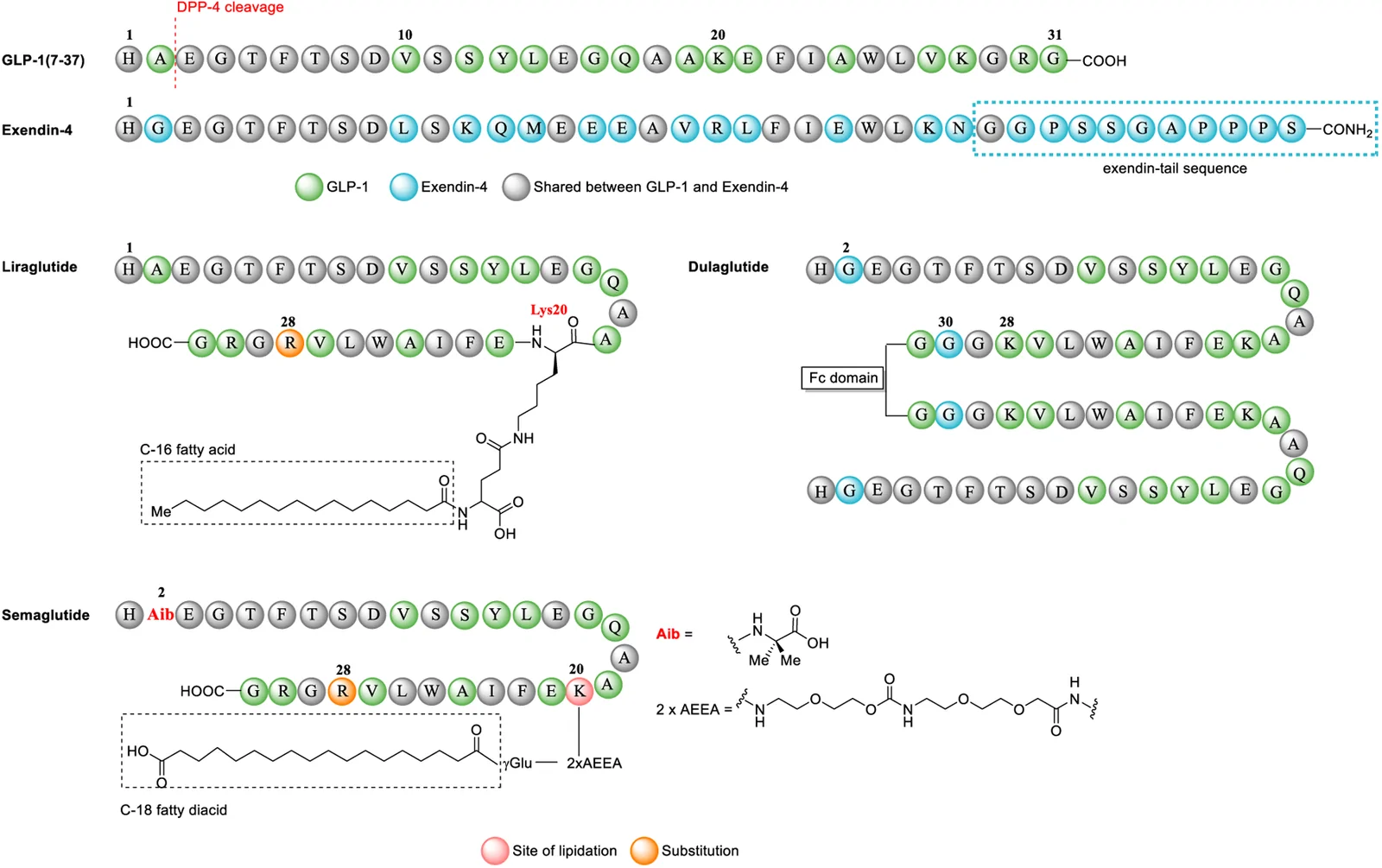
Amino acid sequence of natural GLP-1 and exendin-4, and semi-synthetic derivatives liraglutide, dulaglutide, and semaglutide.

**FIGURE 2:**
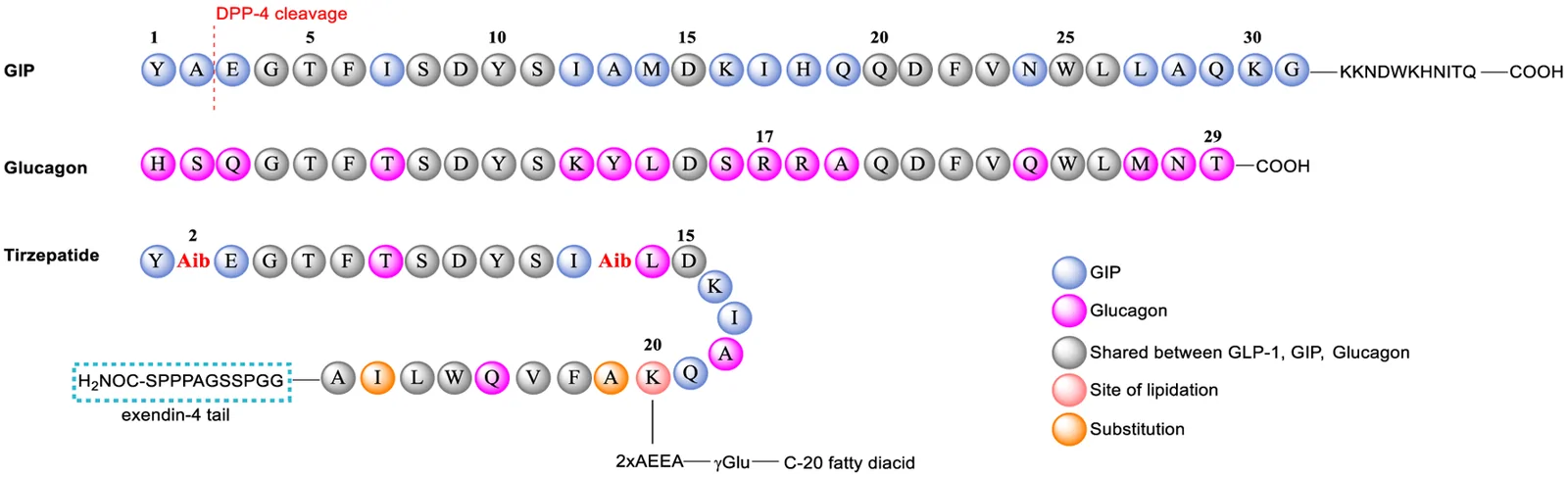
Amino acid sequences of GIP, glucagon and tirzepatide.

Extensive medicinal chemistry programs toward the discovery, optimization, and production of peptide drugs helped to overcome the above-mentioned drawbacks. Synthetic peptides are prepared through both chemical and biological methods to retain the pharmacophore of the native peptide, while improving the pharmacological properties. Typical structural modifications include (non)covalent conjugation to biomolecules (e.g., lipids, short peptide sequences, steroids, *etc.*) and synthetic molecules (e.g., polyethylene glycol (PEG) polymer chains (“PEGylation”), unnatural amino acids to make disulfide bond mimetics, or stapled peptides) (Wang L. et al., 2022; Nielipińska et al., 2024). For instance, a common approach to improve the pharmacokinetic (PK) and pharmacodynamic (PD) profiles of anti-obesity peptides consists in the insertion of albumin-binding moieties able to lower the free fraction of peptides in plasma and interstitial fluid (Jacobsen et al., 2025; Sun X. et al., 2022). Fatty acid conjugation (or lipidation, e.g., semaglutide and liraglutide) (Zhang J. et al., 2021; Kobayashi et al., 2022), direct albumin fusion (e.g., albiglutide, developed by Human Genome Sciences and GlaxoSmithKline) or recombinant fusion with an albumin binding domain (Yousefpour et al., 2020), the use of mycophenolic acid albumin binders (Tang et al., 2020), covalent conjugation to proteins (e.g., fragment crystallizable (Fc) fusion with dulaglutide) of GLP-1 derivatives proved to confer steric hindrance and turned out to be protective against DPP-4 proteolysis. For example, in the design of liraglutide, the fatty acid length was fundamental to enhance the metabolic stability (Madsen et al., 2007). Moreover, replacement of Ala8 with α-aminoisobutyric acid (Aib), or 1-aminocyclobutyl-1-carboxylic acid, or D-serine is a chemical modification exploited to extend the short intravenous half-life, and enhance the stability, and the overall therapeutic potential of GLP-1-based drugs (Zheng et al., 2025b).

Alternative strategies envisaged to compromise the proteolytic activity include: (i) insertion of anti-parallel β-sheet domains at the amino or carboxyl ends of GLP-1 to increase steric hindrance (Sun L. et al., 2022); (ii) the use of an α/β peptide scaffold in the backbone of the construct (Li Petri et al., 2022); (iii) the insertion of fluorinated amino acids at key points; (iv) backbone side chains modifications with saccharides; (v) N-terminus alkylation; (vi) use of extendin-4-hapten conjugates as naturally occurring antibody binders (Dai et al., 2021). Moreover, genetic fusion and peptide conjugation to antibodies are emerging as very promising strategies to increase the hydrodynamic size, and prevent renal filtration, thus favoring neonatal Fc receptor-mediated antibody recycling, and long-acting systems (Wu et al., 2026). Innovative design and delivery systems via conjugation or synthesis of nanoparticles might enable the advancement of this field accelerating the time to the market (Lee et al., 2025).

### 2.1 Natural and semi-synthetic peptides targeting GPCRs in obesity and diabetes

Metabolic disorders are a broad group of diseases in which normal processes of conversion of nutrients into energy and building blocks are disrupted (Sull and McClain, 2025). The impaired glucose and lipid metabolism are correlated to complications like kidney and cardiovascular diseases, foot ulcers, retinopathy, and neuropathy. T2D, obesity, hypertension, and dyslipidemia are common metabolic syndromes that often occur together. Obesity represents a heavy burden on public health and is declared a global epidemic by the World Health Organization (Keller et al., 2026). It is a multifaceted chronic disease driven by the interplay of genetic, environmental, and behavioral factors, and is responsible for shortening life expectancy. Obesity coexists with other comorbidities like non-alcoholic fatty liver, renal and neurological diseases, and some types of cancer (Pocai, 2023). Despite bariatric surgery is still the most effective long-term treatment, its invasive nature and limited accessibility make the pharmacological therapy most desirable. In this scenario, hormonal peptide treatment targeting GPCRs revolutionized obesity care and emerged as a balanced therapeutic scheme for weight management and T2D treatment. By leveraging the body’s natural hormonal mechanisms, these medicaments delivered efficacious weight loss that was impossible to achieve with small molecule drugs. Remarkable results were obtained by using GLP-1R agonists, along with the recently developed unimolecular dual, triple, and tetra agonists targeting simultaneously GIPR, glucagon (GCGR), amylin (AMYR), neuropeptide Y (NPYR, including NPY1R, NPY2R, NPY4R, and NPY5R), glucagon-like peptide-2 (GLP-2R, e.g., dapiglutide) (Nielsen et al., 2026), or melanocortin-4 (MC4R)receptors. Additional non-GPCR endocrine pathways have also emerged as promising targets, including growth differentiation factor 15 (GDF-15) (Zhang et al., 2024), and fibroblast growth factor 21 (e.g., HEC88473) (Zheng et al., 2024; Østergaard, 2026; Moon et al., 2010; Wang S. et al., 2025; Finan et al., 2015) (e.g., HEC88473) (ClinicalTrials.gov, 2025f).

Beyond incretins, other GPCRs are known to contribute to the regulation of glucose homeostasis and are being explored as potential therapeutic targets. Among these, the somatostatin receptor family attracted considerable attention. Somatostatin is a potent inhibitory peptide hormone produced by endocrine cells and by the central nervous system. It exerts its biological function by binding to five different subtypes, promoting inhibition of both exocrine and endocrine secretory functions of the pancreas, suppression of growth hormone secretion, and reduction of the levels of insulin-like growth factor-1. Recent findings showed that selective inhibition of subtypes 2 and 5 improves glucose homeostasis through paracrine regulation of intestinal GLP-1 (Tamura et al., 2023; Jepsen et al., 2021). More GPCRs currently under evaluation as promising therapeutic targets for metabolic syndrome include oxytocin receptor, vasopressin systems, and parathyroid hormone receptor 1 (Izquierdo-Lahuerta, 2021; Elsamad et al., 2026).

#### 2.1.1 GLP-1R ligands

GLP-1R belongs to class B GPCR; is primarily located on pancreatic β cells and in the brain. Its natural ligand agonist is GLP-1 (Figure 1), a 30-amino acid hormone produced in the intestinal epithelial endocrine L-cells by differential processing of proglucagon. Upon binding of GLP-1 to the extracellular domain, the receptor undergoes structure modifications resulting in the exposure of the central region. Subsequently, the transmembrane domain binds to the ligand N-terminal favoring the endocytosis of GLP-1R/GLP-1 complex (Song et al., 2017). Following internalization and endosomal acidification, GLP-1 migrates to the lysosome while GLP-1R redistributes to early endosomes, and, consequently, is recycled to the plasma membrane. Alanine scanning studies on full-length GLP-1 identified His1, Gly4, Phe6, Thr7, and Asp9 as key residues of the N-terminal 11-mer sequence (His1-Ala-Glu-Gly-Thr-Phe-Thr-Ser-Asp-Val-Ser11∼), correlating with >10-fold–10,000-fold decreased potency based on EC_50_ values determined by *in vitro* radiolabeled competitive receptor binding assays and cAMP functional assays of modified analogues (Adelhorst et al., 1994; Gallwitz et al., 1994). Indeed, cryogenic electron microscopy and X-ray analyses showed that GLP-1 adopts an α-helical conformation when bound to its receptor, and its N-terminus binds directly to the active site. Moreover, a few residues from the C-terminal site (i.e., Tyr13, Glu15, Glu21, Phe22 and Ile23) engage key interactions with the receptor extracellular domain, and are crucial for potency (Underwood et al., 2010). Further analysis of mature GLP-1 (7–36) indicated residues 2, 6 and 11 as key determinants of α-helical propensity (Sawyer et al., 2025). At the cellular levels, following binding with GLP-1, Gα protein is recruited leading to downstream cAMP production. Then, β-arrestin is involved for receptor internalization and signal desensitization. GLP-1 plays a crucial role in the management of blood glucose levels, lipid metabolism, and many other biological functions. It stimulates the release of insulin in a glucose-dependent manner leading to β cell mass increase. These two features make targeting GLP-1R a highly effective treatment option for patients affected both by T2D and obesity. However, the underivatized native GLP-1 (7–36 amide or 7–37 acid, Figure 1) has a short half-life *in vivo* (1.5–5 min), primarily caused by the high renal clearance and proteolysis, generating the main metabolite GLP-1 (9–36 amide) with several orders of magnitude lower potency toward GLP-1R. Exenatide (Byetta, by Amylin Pharmaceuticals in collaboration with Eli Lilly, Table 1) was the first in class GLP-1R agonist approved by the Food and Drug Administration (FDA) in 2005 for T2D management (Darwish et al., 2025), and is the synthetic analogue of exendin-4 (Figure 1), a hormone derived from the venom of the lizard Gila monster (*Heloderma suspectum*), with approximately 50% homology to the native GLP-1 (Horowitz et al., 2024). This drug requires a twice-daily injection to provide active circulating concentrations, resulting in patient discomfort. In addition, being a natural toxin, exenatide is immunogenic and can elicit allergic reactions upon administration; indeed, clinical trials showed the production of antibodies against exenatide in treated patients (Zheng et al., 2025b). Liraglutide, semaglutide (both by Novo Nordisk), and dulaglutide (by Eli Lilly) (Figure 1; Table 1) followed as second-generation long-acting molecules with enhanced receptor engagement profiles, longer half-lives, and enabled once-weekly administration, while significantly improving glycemic control and weight reduction (Ansari et al., 2024). Liraglutide (approved by FDA in 2010) was obtained by functionalization with a palmitic fatty acid chain at the position 26 of the native GLP-1 structure, and its design was conceived to extend the action of insulin (Kurtzhals et al., 1995). Such modification helped to protect the drug against DPP-4 cleavage by formation of heptameric oligomers, thus extending its half-life (Østergaard, 2026). However, the low albumin-binding affinity enabled once-daily dosing. Dulaglutide was developed by Eli Lilly and was marketed in 2014 to treat T2D. It is a covalent dimer of GLP-1 obtained by replacing the Ala8 of the native sequence with glycine (Østergaard, 2026). This modification conferred resistance against DPP-4 and allowed once-weekly treatment. Each GLP-1 monomer is fused to the Fc domain of human IgG4 through three repeats of the 4xGly-Ser linker, forming a dimeric version of GLP-1. Despite the efficacy in treating glycemic control, the drug showed modest weight loss. Semaglutide (trade name Ozempic) is a 31-amino acid GLP-1 analogue; it was approved by FDA in 2017 to treat T2D, and, in 2022, for weight control in adults with obesity or overweight with comorbidities (Wegovy®). The use of semaglutide leads to delayed gastric emptying, suppression of appetite through hypothalamic signals, and improvement of cardiovascular, renal, and metabolic parameters. Additional benefits include the high effectiveness in both injectable and oral formulations, wide range of dosing options, lower cardiovascular risks. Substantial evidence supports its benefits in conditions such as hepatic steatosis, sleep apnea, obesity-related heart failure with preserved ejection fraction, and prevention in prediabetic patients (Deanfield et al., 2024). Compared to the endogenous ligand, semaglutide presents three key features: (i) the noncanonical amino acid Aib at position 2 [i.e., Ala8Aib in GLP-1 (7–36)] which confers improved peptide stability by restricting the polypeptide backbone conformations (De Filippis et al., 1998); (ii) a lipid modification on the Lys20 with a C-18 fatty diacid chain attached through a spacer composed of a L-γ-glutamyl (γGlu) unit and two hydrophilic oligoethylene glycol (OEG) linkers [i.e. (2-[2-(2-aminoethoxy)ethoxy]acetic acid (AEEA)] (Salvador et al., 2025). This last chemical modification favors albumin binding, reducing the breakdown, and prolonging its half-life to 1 week. Additionally, (iii) an arginine replaces the lysine at position 28 providing the dual advantage to enhance resistance to enzymatic cleavage without affecting its potency, and to minimize the formation of by-products during manufacturing, by preventing undesired fatty acid acylation at this site, thereby ensuring higher yield and safety production.

#### 2.1.2 GIPR and GCGR ligands

GIPR is a 59 kDa Class B GPCR located on pancreatic β cells, adipose tissue, bone, and brain. Its activation in response to food intake promotes insulin secretion, regulation of blood glucose levels, and whole-body metabolism (Zhang Q. et al., 2021; James-Okoro et al., 2025). Recent findings showed that it can also act centrally via vagal afferents and hypothalamic circuits to reduce food intake and increase satiety (Adriaenssens et al., 2019; Adriaenssens et al., 2023; Smith et al., 2022). Multiple genetic studies performed in humans demonstrated that lower GIPR expression or function is linked to decreased body mass index (Okada et al., 2012; Miyawaki et al., 2002). Moreover, multiple knockout mouse models, including whole-body GIPR, central nervous system, and intestinal K cells knockout exhibited protection against diet-induced obesity mice (Althage et al., 2008; Nasteska et al., 2014). Tirzepatide is the first-in-class dual GLP-1R/GIPR agonist approved by FDA in 2022 for T2D (Figure 2; Table 1), and, in 2023 for obesity and overweight. It is a 39-amino acid synthetic peptide based on GIP amino-acid sequence, containing a C-20 unsaturated diacid acyl chain at Lys20 attached via a linker (2xAEEA− γGlu) (Willard et al., 2020). In clinical trials, the drug exhibited a greater hypoglycemic effect than basal insulin or GLP-1R agonists (e.g., semaglutide and dulaglutide) (Del Prato et al., 2021; Inagaki et al., 2022) with weight loss up to 22.5%. Its efficacy has been attributed to the dual activation of GIPR and GLP-1R signals in the brain, which provide a better glycemic control via acting on pancreatic β cells and insulin secretion; in addition, the GIPR-driven improvements in white adipose tissue seems to contribute to the strong anorexigenic effect.

GCGR is another Class B member with an important role in glucose homeostasis maintenance. When stimulated, the receptor promotes cAMP production and activation of protein kinase A which in turn modulates membrane ion channels, and the activation of transcription factors that influence cell gene expression, particularly in the liver (Welch and Vella, 2024). To date, advanced clinical trials are investigating GCGR dual agonists [e.g., survodutide (ClinicalTrials.gov, 2026i; ClinicalTrials.gov, 2026o; ClinicalTrials.gov, 2026n), pemvidutide (ClinicalTrials.gov, 2026b)] and triple agonists [e.g., retatrutide (ClinicalTrials.gov, 2026l; ClinicalTrials.gov, 2026k)] for obesity, cardiovascular disease, and non-alcoholic steatohepatitis treatments (NASH).

#### 2.1.3 AMYR and NPYR ligands

AMYRs are heteromeric complexes formed by the association of calcitonin receptor (CTR, i.e., a class B1 GPCR) to one of three receptor-activity-modifying proteins, which are a small family of membrane-spanning accessory proteins that interact and alter GPCRs activities. These receptors respond to amylin and calcitonin hormones and represent druggable targets to treat obesity and metabolic disorders. Amylin (also known as islet amyloid polypeptide or IAPP5) is a 37 amino acid neuroendocrine hormone (Figure 3) co-secreted with insulin by pancreatic islet β-cells; after binding to the receptor, it promotes glucoregulatory actions, suppressing appetite, slowing gastric emptying, and enhancing insulin sensitivity, thereby improving insulin resistance (Karra and Batterham, 2010). Analogues of human (h)-amylin proved to be successful in weight management but their clinical use was limited by the intrinsic short-acting nature and poor chemical and physical stability in aqueous solution, being prone to aggregation and deamidation (Fischer Munch et al., 2025). In particular, the amino acid sequence Asn22–Phe–Gly–Ala–Ile–Leu27 of h-amylin favors a process of misfolding, in which monomeric amylin initially generates soluble β-sheet-rich oligomers that may be cytotoxic. These oligomers further mature into elongated structures which finally form insoluble protein aggregates (i.e., the amyloid fibrils) in islets of patients with T2D, some of which are responsible of β cell death and disease progression. In preclinical model, it was shown that, the prevention of fibril formation, for example, by introducing i + 3 or i + 4 salt bridges and proline residues at suitable positions (like in cagrilintide, see Section 3.2.1), ensures better β cell survival, and glucose control (Yan et al., 2006). On the contrary, rodent (r)-amylin fibrillation is limited by the presence of many proline residues at positions 25, 28, and 29 (Figure 3). Pramlintide (developed by Amylin Pharmaceuticals, Figure 3; Table 1) (Fischer Munch et al., 2025) was the first h-amylin analogue to receive regulatory approval in 2005 to treat T2D, and was also evaluated as novel anti-obesity treatment; it was obtained by modifying the native backbone of h-amylin via insertion of proline residues at positions 25, 28, and 29 to afford derivatives less prone to fibrillation (similarly to r-amylin) (Jacobsen et al., 2025; Walker et al., 2025). Calcitonin is another hormone consisting of 32 amino acids secreted from thyroid C cells; it regulates calcium homeostasis through the inhibition of osteoclast activity and consequent reduction in blood calcium levels. In addition, recent findings demonstrated that salmon calcitonin (Figure 3; Table 1), the most widely used variant in clinic, is able to acutely delay gastric emptying and increase energy expenditure. A better control of glucose homeostasis and insulin sensitivity was observed with the use of dual AMYR and CTR agonists (DACRAs, see Section 3.2.2) (Zong et al., 2025; Andreassen et al., 2021; Mohamed et al., 2024; Dahl et al., 2024). Considering the multiple role in metabolic regulation, targeting AMYR has been appealing for the clinical development of anti-T2D and anti-obesity therapeutics. Late last year, a new combination of cagrilintide (i.e., a long-acting amylin analogue by Novo Nordisk), semaglutide and tirzepatide was submitted to FDA under the name CagriSema. Cagrilintide (for structure see Figure 4) was designed by insertion of a fatty acid linker at the C-terminus to limit amyloid formation and extend its half-life while retaining the biological activity (Kruse et al., 2021). The disulfide bond near the N-terminus (positions 2 and 7) proved to be essential to ensure dual activity against both AMYR and CTR. Moreover, cagrilintide exhibited a synergistic effect through co-activation of both GLP-1R and AMYR or GIPR, enabling up to 22%–24% weight loss in clinical trials, a result previously attainable only with bariatric surgery. Several ongoing medicinal chemistry campaigns are focused on dual targeting of AMYR and GLP-1R (Gabery et al., 2025), with some candidates advancing to clinical trial phases (e.g., Novo Nordisk’s amycretin currently in phase 1b/2a clinical trials) (Dahl et al., 2025).

**FIGURE 3:**
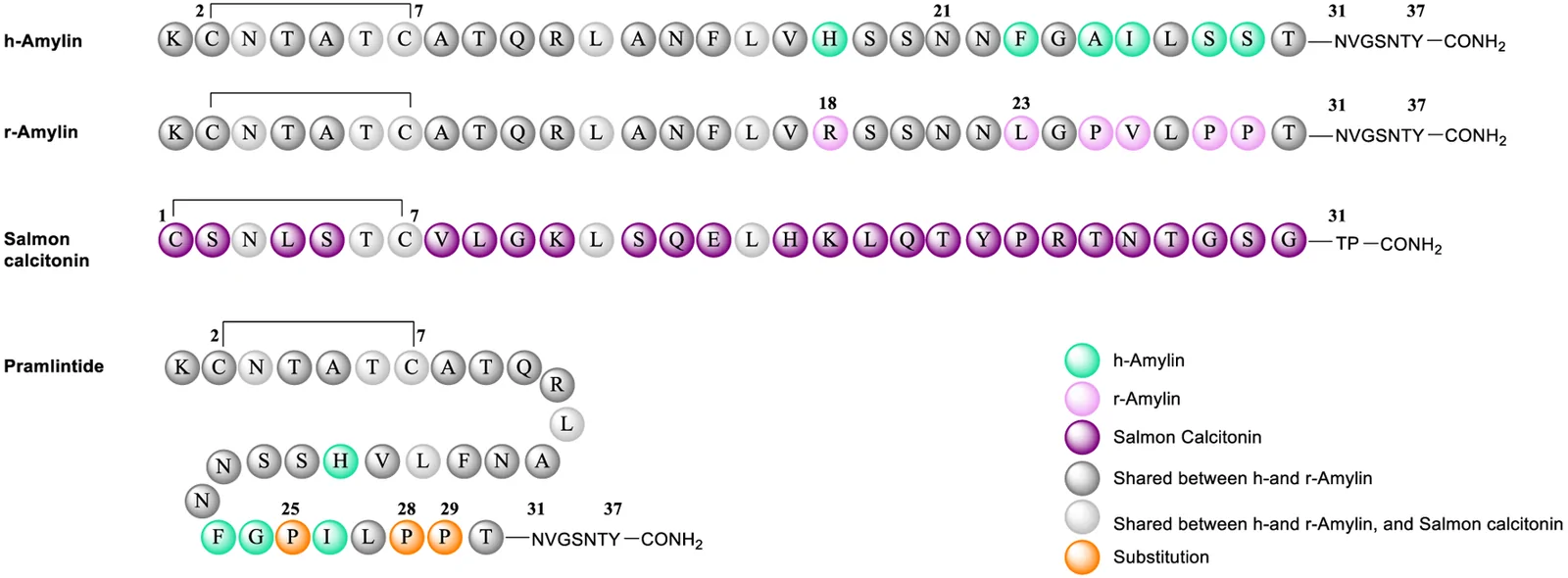
Amino acid sequence of h- and r-amylin, calcitonin and pramlintide.

**FIGURE 4:**
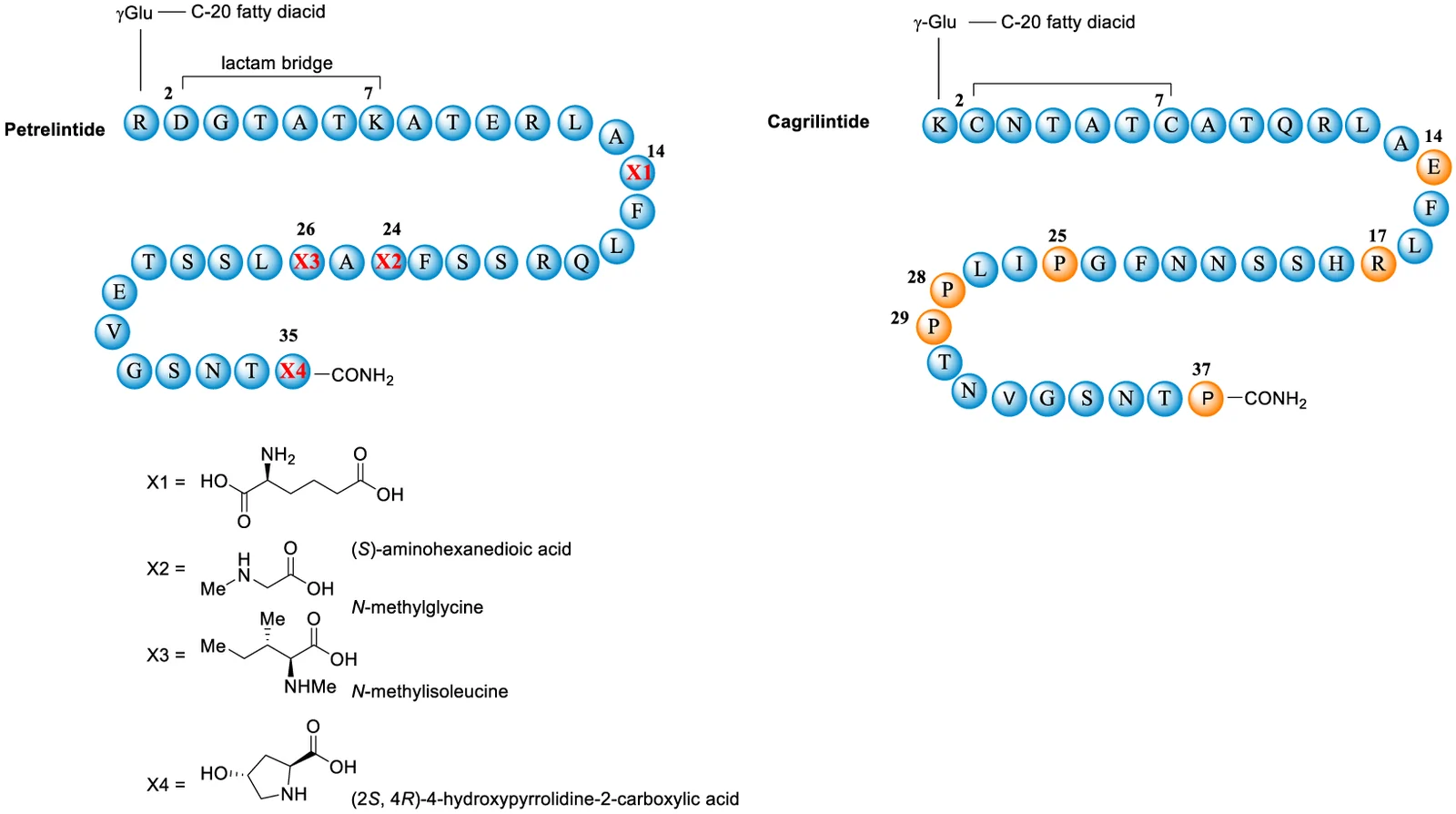
Amino acidic sequence of clinical candidates petrelintide and cagrilintide.

NPYR belongs to Class A GPCRs and is mainly located in pre-synapsis of the central and peripheral nervous system. NPY and PYY (Figure 5a) are its endogenous ligands. NPY is a 36 amino acid peptide characterized by the typical hairpin-like tertiary structure of this family and modulates vital functions like food intake, stress response, and circadian rhythms. PYY is a gut post-meal hormone and is released by enteroendocrine L-cells. Two primary forms of PYY are present in the human body, namely, PYY_1-36_ and PYY_3-36_ (Chen et al., 2023). Upon binding to NPY and PYY (Figure 5), NPYR couples to G_i/o_ proteins to inhibit cAMP production. NPYR activation promotes reduction of food intake, favoring appetite suppression and neuroprotection (Zhu et al., 2024). Many NPYR/GLP-1R dual agonists (see Section 3.1.3.2) currently reached the preclinical phase as promising therapeutic peptides to treat metabolic disorders (Zhang Y. et al., 2021; Liu J. et al., 2025; Xu et al., 2023; Milliken et al., 2021).

**FIGURE 5:**
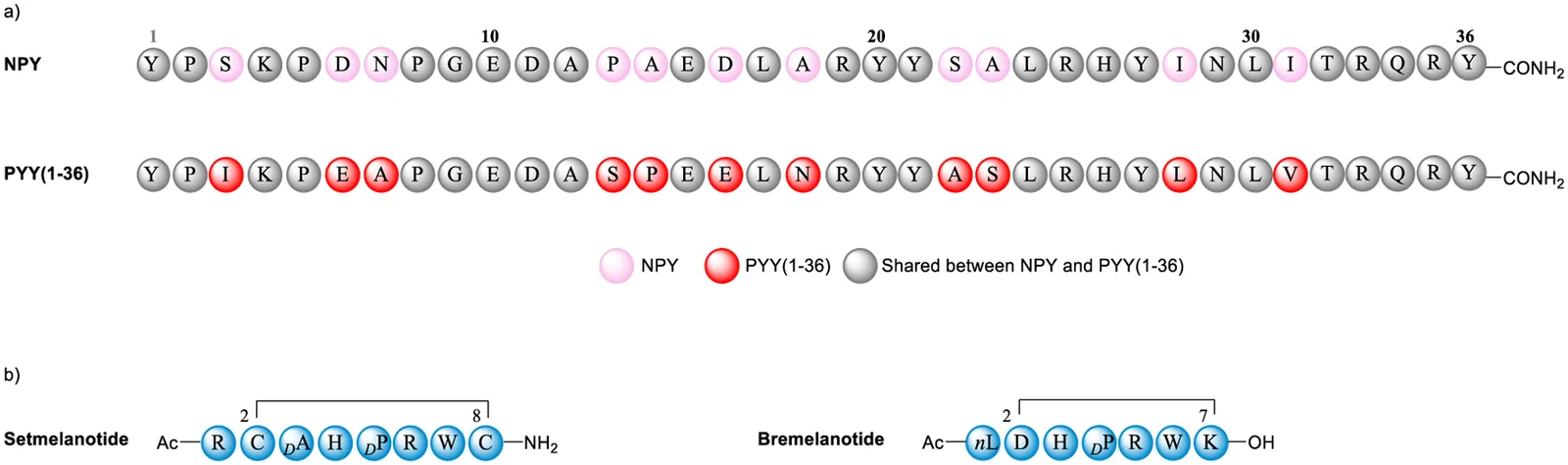
**(a)** Amino acid sequence of NPY and PYY ligands; **(b)** Amino acid sequence of setmelanotide and bremelanotide.

#### 2.1.4 Ligands of MCRs

MCRs are members of GPCRs family and consist of five different subtypes (MC1R to MC5R). α-, β-, γ-melanocyte-stimulating and adrenocorticotropic hormones are the endogenous agonist ligands of each subtype, respectively. These receptors are broadly distributed throughout the body and are involved in a wide number of physiological functions, such as, pigmentation, steroidogenesis, energy homeostasis, exocrine secretion, sexual function, analgesia and inflammation. For instance, MC3R and MC4R are key modulators of energy homeostasis (Girardet and Butler, 2014). MC3Rs contributes to modulate energy homeostasis, fat partitioning, and metabolic health. It is able to influence body’s storage and use of energy rather than directly controlling intense appetite (Seng Lee et al., 2007). On the other hand, genetic studies demonstrated that mutations in the MC4R gene are the most prevalent cause of obesity in humans, highlighting its central role in the regulation of food intake, satiety, and energy expenditure.

Setmelanotide is a selective MC4R agonist; it is a cyclic octapeptide featuring an acetylated N-terminus, an amidated C-terminus, and a disulfide bridge at positions 2–8 (Figure 5b). It was approved by FDA in 2020 for chronic weight management in specific rare genetic diseases and acquired hypothalamic obesity (U.S. Food and Drug Administration, 2022). Bremelanotide is another MC4R agonist (Figure 5b); it is currently under clinical evaluation for obesity treatment in combination with the dual GLP-1R/GIPR agonist tirzepatide, showing synergistic weight-loss effects (ClinicalTrials.gov, 2025e; Spana et al., 2022; Palatin, 2024).

### 2.2 Peptide drugs targeting GPCRs: limitations and challenges

Peptide therapeutics targeting GPCRs revolutionized the treatment landscape of metabolic disorders; however, their clinical application is still hampered by several challenges, and by the marked heterogeneity of therapeutic responses. Indeed, durable weight loss is not consistently achieved across all individuals. Major limitations concern the PK profile, such as, low oral bioavailability, enzyme degradation, plasma instability, poor permeability through the gastrointestinal epithelium, and consequent poor oral delivery. Moreover, proteolytic enzymes favor the rapid clearance in the liver and other body tissues, reducing their *in vivo* half-lives. Subcutaneous and intramuscular injections are preferred routes of administration, and, currently, semaglutide is the only approved oral GLP-1R agonist with 0.8%–1% of absolute bioavailability. Co-formulation with the absorption enhancer sodium *N*-(8-[2-hydroxybenzoyl]amino)caprylate protects this peptide from stomach acid, and facilitates its absorption through the gastric mucosa. On the other hand, subcutaneous administration generally enables moderate to high systemic exposure, with absolute bioavailability values of approximately 89% for semaglutide, 80% for tirzepatide, 55% for liraglutide, and 47%–65% for dulaglutide, depending on the administered dose (1.5 mg and 0.75 mg dose, respectively). Nevertheless, this administration route is often poorly accepted by patients and may increase the risks of immunogenic effects. In addition, strategies used to extend the half-life of the peptides, such as, PEGylation, lipid conjugation, or the use of unnatural amino acids may trigger the adaptive immune system. For example, phase 3 clinical trials of tirzepatide showed a higher incidence of immunogenicity, with anti-drug antibodies production in 51.1% of treated patients. However, this did not affect the PK or efficacy of the drug, and hypersensitivity or reactions at the injection site had mild to moderate severity (Mullins et al., 2024). Pramlintide resulted highly immunogenic; however, these immune responses rarely impact the clinical efficacy, safety, or glycemic control. Instead, clinical trials of semaglutide demonstrated that the incidence of induction of anti-drug antibodies was less than 1%, and was not linked to cases of loss of glycemic effect or hypersensitivity (Marso et al., 2016).

Receptor desensitization is another relevant limitation associated to GPGR targeting peptides, especially following chronic treatments. In general, GPCR desensitization is a regulatory mechanism which protects cells from receptors overstimulation. The repeated or prolonged stimulation is able to promote receptor phosphorylation, β-arrestin recruitment, and receptor internalization, ultimately attenuating G protein-mediated signalling, and leading to a reduction in therapeutic efficacy over time, and to the drug tolerance as longer-term consequence. This phenomenon is typical of some subfamilies (e.g., SCR, oxytocin receptor), but results less pronounced for currently approved GLP-1R agonists, due to the efficient receptor recycling, and sustained endosomal signalling. Indeed, biased GLP-1R agonists successfully overcome receptor adaption bypassing pathways that cause desensitization or adverse side effects (Rankovic et al., 2016). Nevertheless, GPCR desensitization is still an important aspect to consider in the design of next-generation GPCR-targeting peptide drugs.

The development of effective delivery strategies still remains an unmet clinical need. Ongoing developments in structural modifications and delivery systems show potential for enabling oral peptide formulations with enhanced stability, bioavailability, and patient compliance. Notably, recent advancements focus on the development of innovative GPCR-targeted peptide-drug conjugates (European Commission, 2025), and on the design of other delivery platforms, including nanoparticles (see Section 3.1.7) and liposomes (Li et al., 2025).

Finally, the structural similarities among GPCR family members may cause off-target interactions, leading to unwanted receptor engagement, particularly following prolonged exposure and/or at high systemic concentrations. Such off-target activity may rise to safety concerns, hampering the therapeutic window of the drugs, and the achievement of optimal receptor selectivity is one of the main goals to ensure patient safety. For instance, the most common symptoms observed with the use of GLP-1R agonists are mainly related to the gastrointestinal system (e.g., nausea, vomiting, diarrhea, constipation, abdominal cramps, gastroparesis). Table 1 summarizes the therapeutic benefits and adverse effects of FDA approved peptide drugs used to treat obesity and diabetes. Prolonged therapy with semaglutide has been linked to more severe side effects, such as, the insurgence of acute pancreatitis, anesthetic risks like pulmonary aspiration or residual gastric content, acute kidney and gallbladder injury, non-arteritic anterior ischemic optic neuropathy, and diabetic retinopathy. Moreover, recent findings showed that retatrutide is able to increase the heart rate by 6.7 beats per minute, probably producing a more pronounced chronotropic effect compared to semaglutide and tirzepatide. Further studies are required to understand and assess the molecular mechanisms and clinical implications of GPCRs targeting peptides, and optimize their long-term safety profile.

## 3 Recent medicinal chemistry advances toward the discovery of novel peptide drugs targeting GPCRs

GPCRs ensure direct pharmacological action due to the presence of druggable sites at the cell surface; while their coupling to intracellular effectors and second messengers enable efficient signal transduction, and facilitate targeted therapeutic intervention. Indeed, modulation of these receptors has been validated to treat a broad spectrum of human diseases, including cancer, inflammatory disorders, metabolic, central and peripheral nervous system diseases. Hence, it is not surprising that, starting from the early 20th century, GPCRs have been of particular long-standing interest for the scientific community, and still represent one of the major areas of pharmaceutical research.

### 3.1 GLP-1R peptide agonists recently developed

Long-term use of semaglutide is often accompanied by low oral bioavailability, dose-dependent gastrointestinal side effects, high costs. To overcome these limitations, novel optimized GLP-1R agonists were recently designed including fatty acids conjugation, and showing a promising superior efficacy compared to the parent peptide in treating T2D, obesity, and NASH triad (Wong M.-T. et al., 2025). When designing a conjugate, the optimization of the fatty acid spacing, the site for albumin binding, and the drug efficacy are key aspects. Table 2 summarizes peptides targeting GPCRs (mainly GLP-1R and AMYR) developed in the past 5 years, and currently in clinical trials.

**TABLE 2:** Peptide drugs targeting GPCRs recently advanced in clinical trials.

Drug	Peptide template	Key chemical modifications^a^	GPCR	Mechanism of action	Indications	Clinical trial	Advantages	Side effects
Ecnoglutide	GLP-1 (7–37)	Ala8Val 2xAEEA-γGlu-C-18 fatty diacid at Lys24	GLP-1R	Biased for induction of cAMP signalling over GLP-1R internalization	Diabetes	Phase 3^b^ ^,^ ^c^	Effective glycated hemoglobin reduction, potential monotherapy (once-weekly injection)	Mild-to-moderate gastrointestinal symptoms
Pemvidutide	Engineered glucagon	Ser2Aib E16-Lys20 lactam bridge Met27Leu Asn28Gln, 1-*O*-[(17-carboxyheptadecyl)oxy] β-*D*-glucopyranuronoyl)-C-18 fatty diacid at Lys17	GLP-1R/GCGR	GLP-1R/GPCR dual agonism	Obesity, NASH, alcohol use disorder	Phase 2^d^	Direct action on the liver by stimulating fatty acid oxidation and suppressing lipogenesis	Gastrointestinal symptoms
Retatrutide	GIP	Ala2Aib Ala13αMeLeu Gln20Aib AEEA-γGlu-C-20 fatty diacid at Lys17 C-terminal extendin-4 tail	GLP-1R/GCGR/GIPR	GLP-1R/GIPR/GCGR triple agonism	Obesity, diabetes, metabolic liver diseases	Phase 3^e^	Effective body weight reduction, improvements in glycemia, knee osteoarthritis pain, obstructive sleep apnea, effective glycated hemoglobin reduction (in 24 weeks)	Gastrointestinal symptoms, dysesthesia, dose-dependent heart rate increase Rare: Acute pancreatitis, gallbladder disease, thyroid tumors
MariTide	GLP-1, GIP	hIgG1 monoclonal antibody against hGIPR conjugated at E384C to two peptide GLP-1R agonists ([Aib8; Tyr16; Glu22; Gly36](G4S)3Lys) via amino acid linkers	GLP-1R GIPR	GIPR antagonism GLP-1R agonism	Obesity, diabetes	Phase 2/3^f^	Effective body weight reduction (12.3%–16.2% at 52 weeks), effective glycated hemoglobin reduction	Gastrointestinal symptoms
Petrelintide	h-Amylin	Lys1 replacement with Arg-γGlu-C-20 fatty diacid, Gly24N-MeGly, Ile26(NMe)Ile, Tyr37Pro, Asn21 and Asn22 deletion, Asn3 Gly, Asn14(*S*)-aminohexanedioic acid, Asn31Glu, and Asp2-Lys7 lactam bridge	AMY_1_R AMY_3_R CTR	Long acting AMYR and CTR agonism	Obesity, diabetes	Phase 2^g^	Body weight reduction (up 10.7% at week 42% vs.1.7% with placebo)	Mild gastrointestinal symptoms
Cagrilintide	h-Amylin	Asn14Glu, Val17Arg, Ala25Pro, Ser28Pro, Ser29Pro, Tyr37Pro, γGlu-C-20 fatty diacid at Lys1	AMY_1_R, AMY_2_R, AMY_3_R CTR	Long acting AMYR and CTR agonism	Obesity, diabetes	Phase 3^h^	Delayed gastric emptying, better glucose stability, lower blood glucose spikes by glucagon inhibition, synergistic effect with semaglutide in sustained weight management	Gastrointestinal symptoms, fatigue

^a^ Amino acid numbering is based on the sequence of the natural ligand.

^b^ Already approved in China in 2026 for diabetes and obesity.

^c^ NCT05680155 (ClinicalTrials.gov, 2025g).

^d^ NCT04561245 (clinicalTrials.gov, 2025a), NCT05292911, (clinicalTrials.gov, 2023c), NCT05006885 (clinicalTrials.gov, 2023a; Harrison et al., 2025), NCT04972396 (clinicalTrials.gov, 2025d), NCT05989711 (ClinicalTrials.gov, 2026b), NCT07009860 (clinicalTrials.gov, 2026h).

^e^ NCT05929066 (clinicalTrials.gov, 2026j; Abdrabou Abouelmagd et al., 2025).

^f^ NCT07226765 (clinicalTrials.gov, 2026e), NCT06858839 (clinicalTrials.gov, 2025c), NCT07037459 (clinicalTrials.gov, 2026d), NCT06858878, (clinicalTrials.gov, 2025b; Jastreboff et al., 2025).

^g^ NCT06926842 (ClinicalTrials.gov, 2026a).

^h^ NCT04940078 (Lau et al., 2021; clinicalTrials.gov, 2025j).

Wong and co-workers developed a versatile multi-arm linker forming a two-fatty acid (FA in Figure 6) bundles including two lysine and two PEGylated residues with a reactive N-terminal alkyne or maleimide, and an esterified or acidic C-terminal site for peptide-drug attachment. The two fatty acids with identical or differing chain lengths and/or terminal groups were bound to the lysine ε-amino groups of the linker through a glutamate motif, and different configurations were explored modifying (i) the conjugation linkage (alkyne−azide or maleimide−thiol), (ii) the fatty acid chain length (C-16, C-18, or C-20), (iii) the fatty acid terminal group (methyl o carboxylate), (iv) the spacing (n = 2, 4, or 6 ethylene glycol units), and (v) the linker C-terminal group (hydrogen or methyl). All the synthesized bundles were conjugated to semaglutide template at Lys20. Most of the newly obtained analogues with an esterified C-terminus displayed stronger albumin binding compared to the reference semaglutide, thus suggesting that the explored combinations were able to increase hydrophobic and electrostatic interactions with albumin’s multiple fatty acid binding sites. Peptide **1** (Figure 6) emerged as the most promising and, in diabetic mice, showed better longer-lasting glycemic control, and long-term efficacy in decreasing blood glucose levels and reduce fatty liver disease symptoms, compared to semaglutide. Furthermore, peptide **1** stimulated insulin secretion under high blood sugar conditions; at low doses (5 nmol/kg), it decreased liver fat content and improved liver health, demonstrating an overall enhanced *in vivo* efficacy in both diabetic and obese models, a low-dose reduction of liver steatosis, and increased liver health in NASH mice, highlighting the potential of the multi-arm linkers to provide a versatile platform for the generation of optimized fatty acid conjugate peptide therapeutics.

**FIGURE 6:**
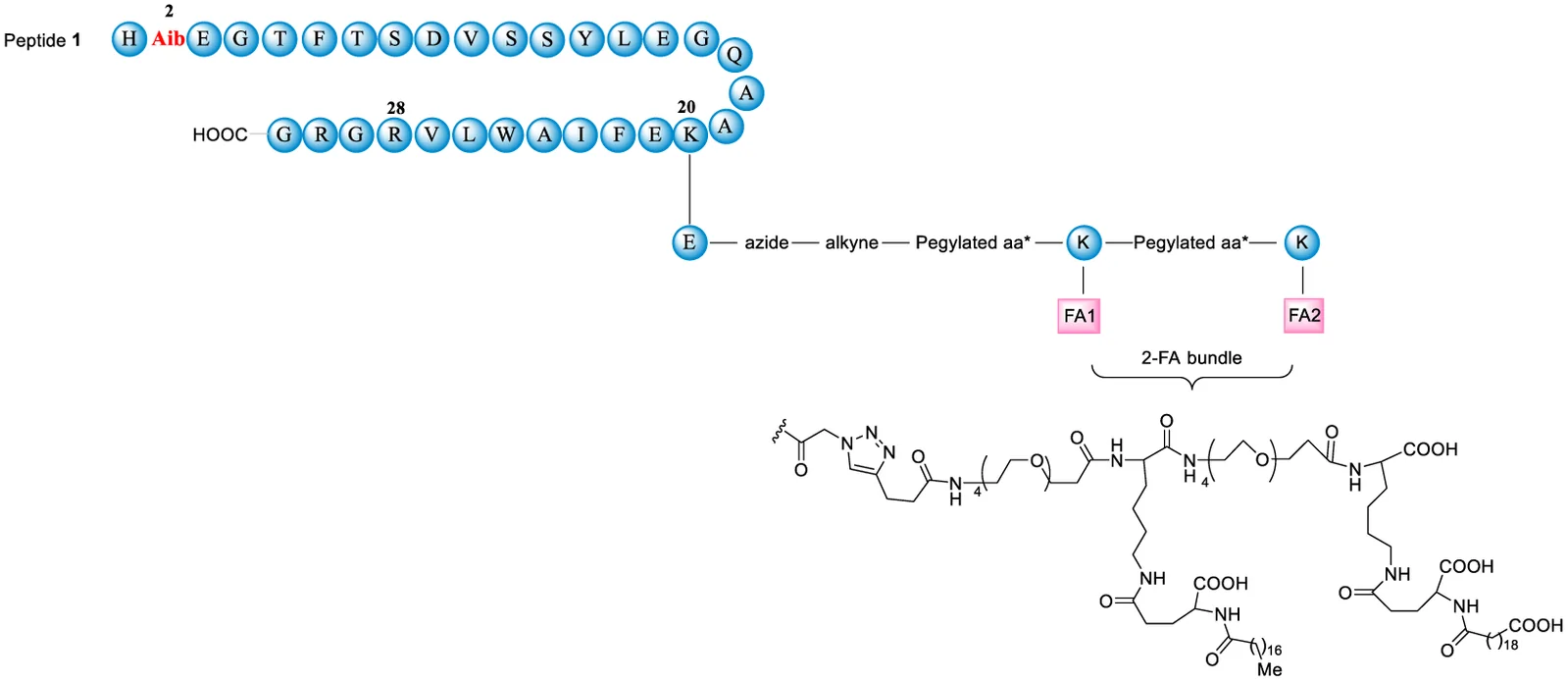
General structure of peptide **1** (*: amino acids).

Starting from the crystal structure of semaglutide in complex with GLP-1R (Zhang X. et al., 2021), Wei and collaborators applied a new approach that coupled deep learning-based protein design with functional screening, to obtain novel ultra-long acting agonists with improved stability and efficacy (Wei et al., 2025). *De novo* peptides were designed in combination with ProteinMPNN (Dauparas et al., 2022), via modifying the Gly31 C-terminus, and by retaining the critical hotspots of the reference ligand necessary for target recognition, binding at the transmembrane core, and receptor activation. Several promising candidates with greater diversity were selected for *in vitro* and *in vivo* validation studies, among which, peptides **2–4** (Figure 7) emerged as the most interesting, with EC_50_ values of 0.011, 0.012, and 0.036 nM, respectively, in cAMP accumulation assay (vs. 0.019 nM for semaglutide). They exhibited binding affinity comparable to the native ligand, with dissociation constant (K_D_) in the order of 10^−6^ (8.58 10^−6^, 5.19 10^−6^ and 6.78 10^−6^, respectively vs. 2.78 10^−6^ for semaglutide). *In vivo*, compounds **2** and **4** showed improved, half-life (t_1/2_ = 23.16 h and 19.86 h respectively), and extended time required to reach maximum plasma concentration (T_max_ = 12 h and 10.67 h, respectively) vs. semaglutide (t_1/2_ = 8.17 h; T_max_ = 8.17 h). In diabetic mice, peptides **2** and **4** exerted a prolonged hypoglycemic effect; of note, **4** significantly reduced blood glucose levels, in both single- and multiple-dose regimens, with a less pronounced weight loss, promoting artificial intelligence-powered high-throughput protein design as a promising way to improve stability and efficacy of biotechnological peptide drugs.

**FIGURE 7:**
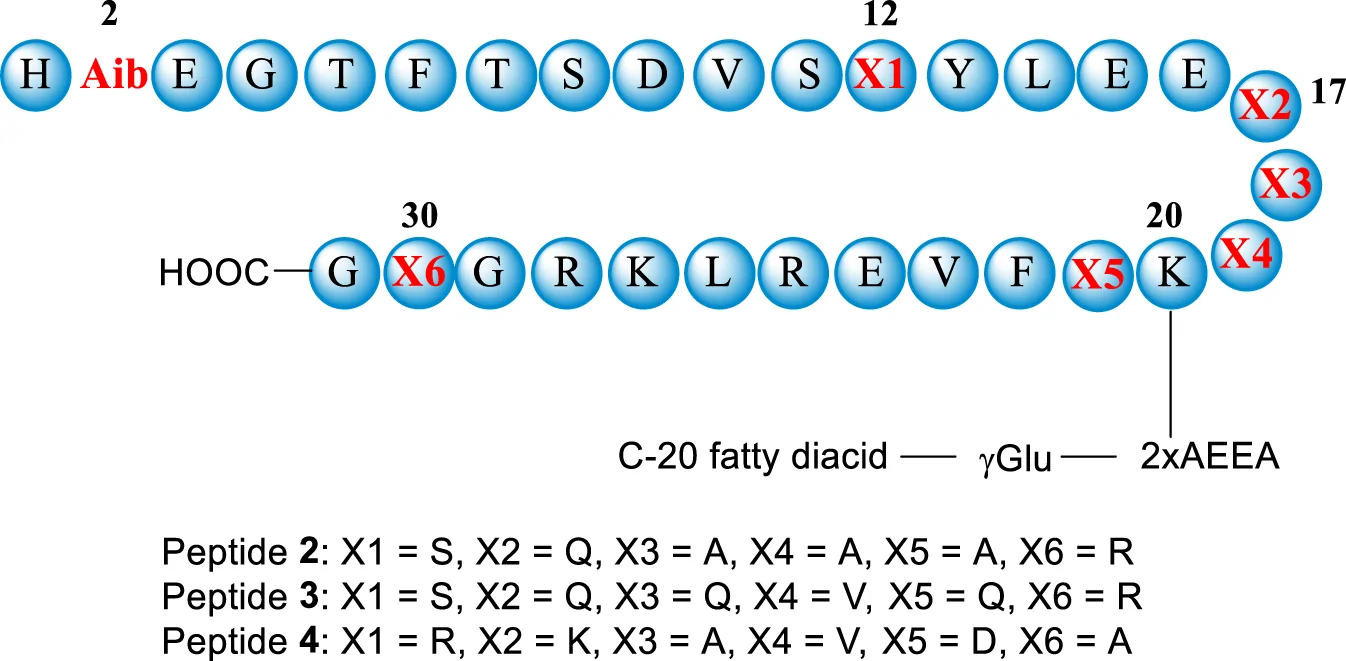
Rational design of peptides **2–4** from semaglutide template.

The commercial production of semaglutide is carried out through semi-synthesis, by initial recombinant expression of the peptide (9–37) in yeast, followed by chemical synthesis to introduce the fatty acid side chain, and final coupling of the N-terminal peptide. Conjugation of the fatty acid content makes the sample preparation and large-scale purification very challenging and expensive. Chandrashekar and co-workers developed a new bioactive glycosylated GLP-1 (**5**, Figure 8) by a cost-efficient chemoenzymatic peptide synthesis, as potential alternative to semaglutide (Chandrashekar et al., 2024).

**FIGURE 8:**
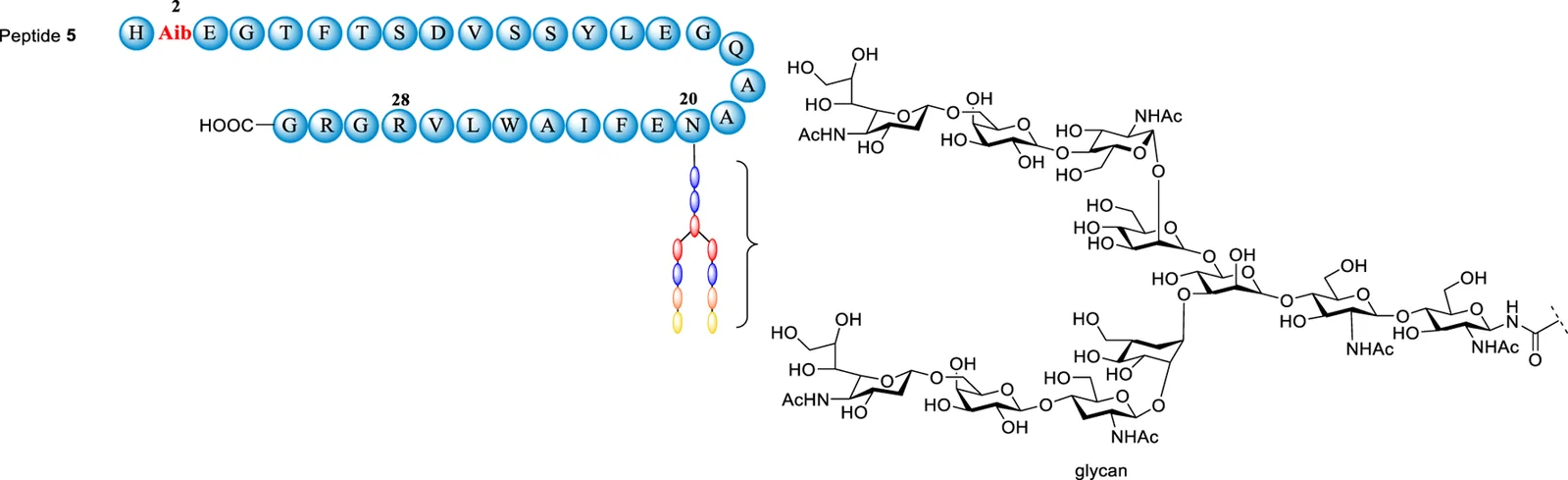
General structure of the new bioactive glycosylated GLP-1 analogue **5**.

To this purpose, omniligase-1 analogue, that is a peptiligase variant with broad substrate specificity, was used, and, starting from semaglutide template, the new peptide was obtained by replacing the Lys20 with Asn20, and by conjugation to the hydrophilic glycan Fmoc-Asn (dibenzyldisialyloligosaccharide)−OH, which was isolated from hen egg yolk, given its known beneficial effects on solubility, half-life, and *in vivo* stability (Nishiuchi et al., 2025; Chandrashekar et al., 2023). The glycoGLP-1 analogue was evaluated for its efficacy in glycemic control and, after subcutaneous administration in rat, elicited a rapid and sustained reduction in blood glucose levels (compared to the parent semaglutide), with a prolonged effect up to 2 h after injection, indicating that glycosylation of GLP-1 analogues represents an effective strategy to generate compounds more synthetically accessible with low production costs; while maintaining therapeutic efficacy comparable to semaglutide which, to date, is globally highly demanded.

In line with this, Xu et al. developed a novel synthetic biology plan for engineering semaglutide based on a post-translationally modified peptide technology to obtain new GLP-1R agonists to deliver via intestinal flora (Xu and Kuipers, 2025). These peptides were synthesized by the ribosomes followed by post-translational modifications, such as, dehydration, hydroxylation, glycosylation, and insertion of noncanonical amino acids conferring the final bioactive properties. Key design elements included: i) the insertion of ornithine at position 2 of semaglutide backbone to improve resistance to enzymatic degradation and consequent metabolic stability, thus exploiting the conformational change due to the presence of a noncanonical amino acid (structurally similar to lysine), and preventing enzyme recognition, and peptide bond cleavage; ii) introduction of an azide-containing amino acid at position 20, enabling click chemistry functionalization, given the evidence that aromatic amino acids had stronger binding affinities to human serum albumin (hSA) (Ding et al., 2024). Ornithine was successfully introduced *in vivo* through the catalytic conversion of arginine by the peptide arginase OspR, demonstrating the feasibility of introducing noncanonical residues via post-translationally modified peptide biosynthesis. This was the first example of ornithine-modified semaglutide analogue, with the simultaneous insertion via click chemistry of a handle at amino acid 20 for bio-orthogonal conjugation (e.g., lipidation) into the semaglutide core-encoding sequence (sem-2R). The approach enabled modular post-synthetic functionalization as scalable route to expand the chemical space of GLP-1 analogues.

In the same context, Bird and co-workers evaluated the insertion in semaglutide template (from C-terminus to N-terminus) of *S*-octenyl alanine, bis-pentenyl glycine, and *R*-octenyl alanine residues at sequential i, i + 7 positions to increase the proteolytic protection (see Figure 9) (Bird et al., 2020). The newly synthesized hydrocarbon-stitched peptide agonists (30 amino acids length) showed a helical structure with the N-terminal segment sitting deeply within the receptor binding pocket, and with the C-terminal participating in a more traditional helix-in-groove interaction with the extracellular domain of GLP-1R (Underwood et al., 2010).

**FIGURE 9:**
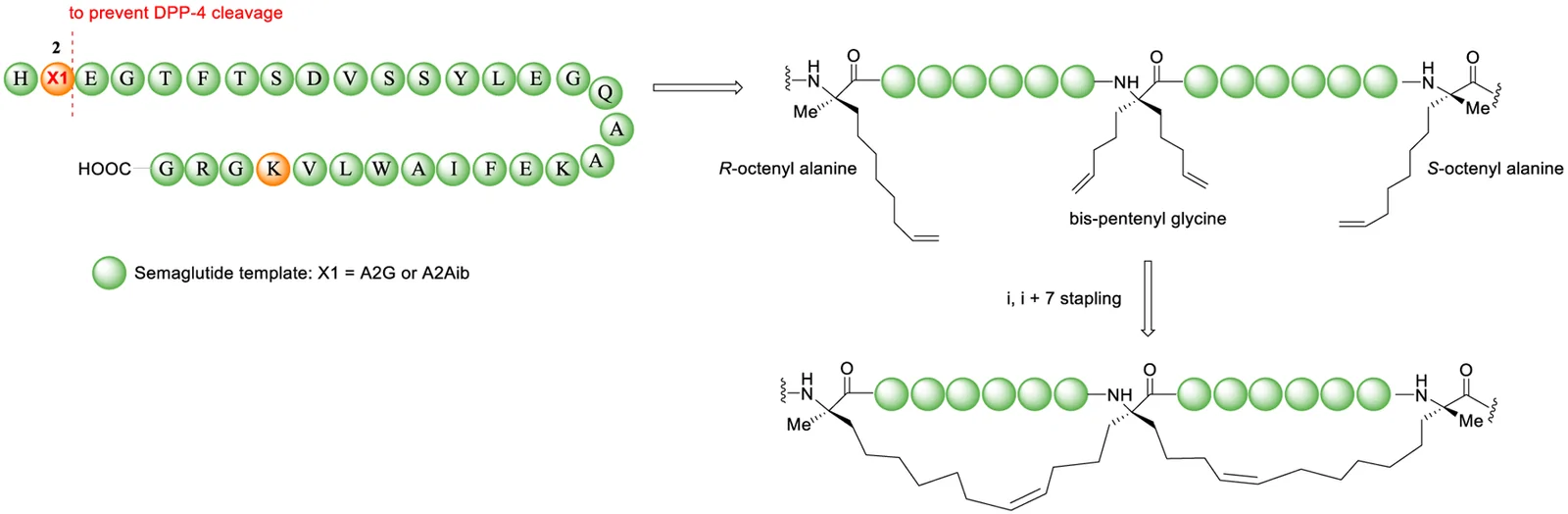
Schematic representation of novel i, i + 7 hydrocarbon-stitched GLP-1R agonists.

Optimization studies led to the stabilized alpha-helix construct, in which the alanine residue of the dipeptide Ala2-Glu3 was replaced by the Aib (Deacon et al., 1998), confirming its ability to bind GLP-1R, thus resulting in cAMP levels increase (EC_50_ value of 160 pM). Moreover, when evaluating the impact of the installed i, i + 7 stitch on the secondary structure of the template by circular dichroism, the semaglutide template containing the 10,17,24 stitches demonstrated to recall the GLP-1 α-helicity. *In vitro* proteolytic degradation assay (using the proteinase K as highly active and broad-spectrum serine protease) showed high protease resistance, supporting the hydrocarbon stitching as a valuable tool to design lengthy, α-helical peptides with drug-like properties.

#### 3.1.1 Novel GLP-1R agonists of natural origin

Han and co-workers identified new GLP-1 analogues from amphibians, known to be the furthest species from mammals utilizing the endogenous ligand for insulinotropic purposes (Jiang et al., 2024). Among all, bullfrog GLP-1 (bGLP-1, Figure 10) showed the highest potency (EC_50_ value of 0.051 nM in GLP-1R activation assay) and notable *in vivo* acute hypoglycemic effect in ICR mice. On bGLP-1 template, alanine scanning studies from position 10 (to ensure a conservative N-terminal sequence required for GLP-1R activation) afforded novel derivatives (Bueno et al., 2020). Peptide **6** (EC_50_ value of 0.018 nM in GLP-1R activation assay, Figure 10) derived from Ala2 substitution displayed enhanced potency in activating GLP-1R, and was selected for further modifications. To this purpose, two distinct hSA binders, namely, C-18 diacid-γGlu-2xAEEA and palmitic acid-γGlu, on the Lys17 or Lys20 residues, respectively, were evaluated to enhance the stability (Knudsen and Lau, 2019; Lau et al., 2015). Moreover, Ala2 was replaced with Aib2 to boost resistance toward DPP-4 enzymatic proteolysis. Peptide **7** featuring a C-18 diacid-γGlu-2xAEEA albumin binder, and Glu17 substitution showed a 2-fold higher activity (EC_50_ value of 0.019 ± 0.005 nM in GLP-1R activation assay) compared to the reference compound semaglutide (EC_50_ value of 0.052 nM). In addition, it exhibited a persistent hypoglycemic effect in nonfasted diet-induced obesity mice, demonstrating a potent and sustained glucose-lowering activity throughout the entire experimental period (48 h), and superior hypoglycemic effect. In ICR mice, this peptide showed a dose-dependent decrease in food intake, and, a dose-dependent anti-hyperglycemic action, and beneficial effects on body weight, glucose homeostasis, and lipid metabolism, in diet-induced obesity mice, thus emphasizing the advantage to develop from natural sources novel antidiabetic and anti-obesity agents with superior therapeutic benefits than conventional drugs.

**FIGURE 10:**
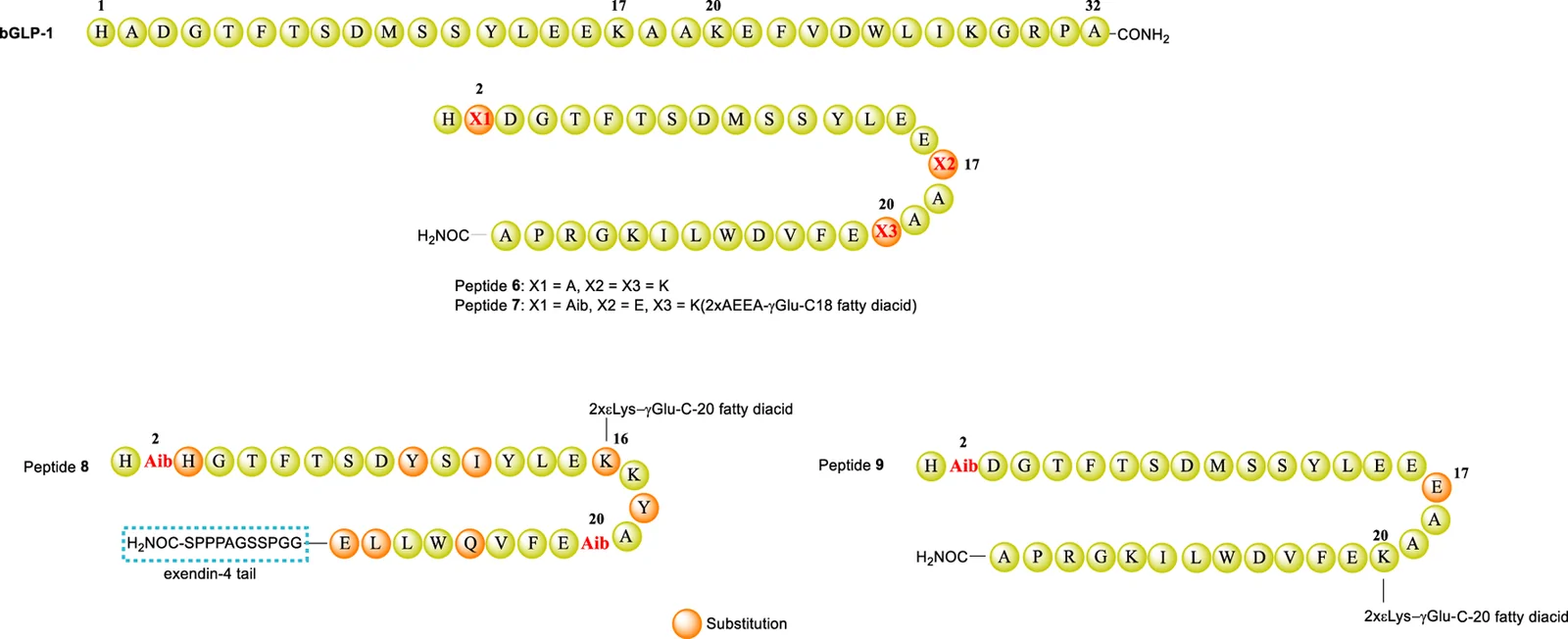
General structure of novel GLP-1R agonists **6–9** derived from bGLP-1 template.

More potent and long-acting bGLP-1 analogues were designed and synthesized by Sun and co-workers (Sun et al., 2024), on the basis of the promising results obtained with peptide **8** (Figure 10) (Jiang et al., 2024). In addition to the fatty diacid linker εLys-εLys-γGlu-C-20 used for peptide **8** (Knerr et al., 2022), four more albumin binders were evaluated with the goal to improve the metabolic stability of the newly designed peptides, including 2xAEEA-γGlu-C-20 fatty diacid [used in tirzepatide (see Figure 2)], AEEA-γGlu-C-20 fatty diacid [used in retatrutide (see Figure 11)], γGlu-γGlu-Ser-Glu-Ser-γGlu-γGlu-C-18 fatty diacid (Simonsen et al., 2022), Gly-Gly-Ser-Gly-Ser-Gly-C-18 fatty diacid (Zimmermann et al., 2022). Again, fatty acid modification was explored on Lys17 and Lys20 residues. Furthermore, on peptides including fatty acid modification at Lys20, Lys17 amino acid was replaced by Glu17 being favorable for the biological activity (Han J. et al., 2018). Moreover, Ala2 residue was replaced by Aib2 to increase the resistance against degradation by DPP-4. Compounds acylated at Lys20 exhibited better potency compared to the analogues with acylation at Lys17. The albumin binder εLys-εLys-γGlu-C-20 fatty diacid resulted the optimal choice to achieve long-acting modifications of bGLP-1. Assessment of the *in vitro* affinity toward hGLP-1R was performed in the absence (to measure the true receptor affinity without interference from albumin affinity) or in the presence of hSA (to evaluate the albumin binding abilities). Peptide **9** emerged as the most promising compound and was more potent than its precursor **8** (IC_50_ values of 1.5 nM vs. 3.3 nM (for 0% of hSA), and 453 nM vs. 578 nM (for 2% of hSA) in GLP-1R activation assay). In Kunming mice, peptide **9** efficiently reduced glucose excursion in 2 h period post-glucose (P < 0.001), with a hypoglycemic effect similar to semaglutide at 30 nmol/kg dose. Next, in diet-induced obesity mice, the hypoglycemic effect of peptide **9** was higher than semaglutide at the dose of 10 nmol/kg during the 2 h period. Moreover, in a 3-week study in diet-induced obesity mice, to evaluate the impact of peptide **9** alone or in combination on lipid metabolism, glucose regulation, and body weight, co-administration of peptide **9** with cagrilintide yielded enhanced weight loss effects, leading to 38% body weight reductions and greater reduction in food intake, and significantimprovement in glucose tolerance (compared to the group treated with semaglutide in combination with cagrilintide), demonstrating the great potential of using bGLP-1-derived GLP-1R agonists as effective partners for amylin analogues, and paving the way for new combination therapies in diabetes and obesity management.

**FIGURE 11:**
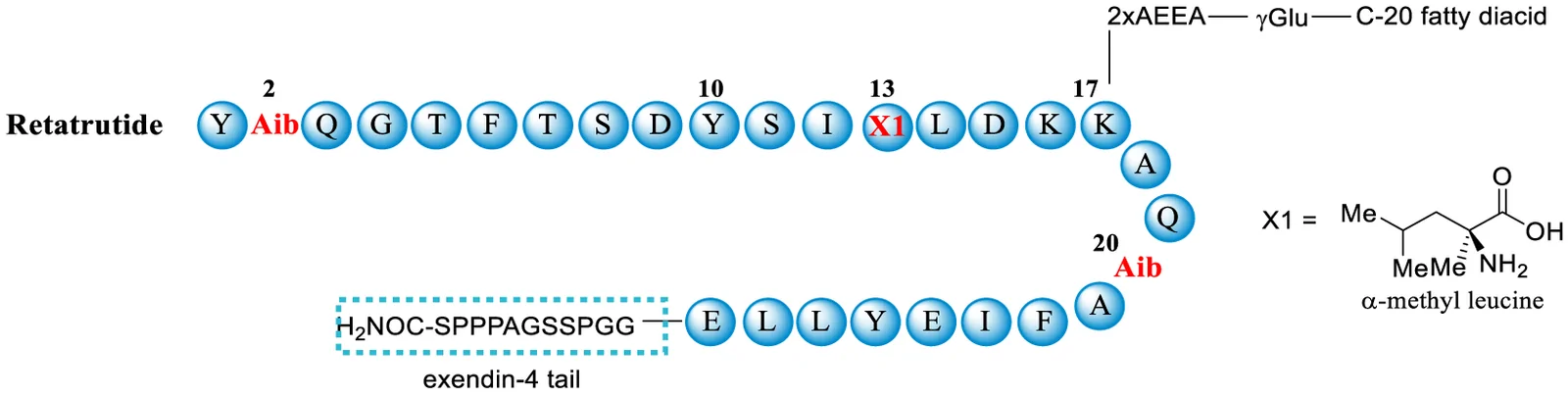
General structure of the clinical candidate retatrutide.

#### 3.1.2 Biased GLP-1R agonists

Balanced agonism refers to the conventional classification of native ligands, like GLP-1 and exendin-4, able to similarly activate both Gα/cAMP signalling and β-arrestin recruitment (Douros et al., 2024). On the contrary, biased ligands selectively activate a specific downstream pathway. For instance, signalling bias at GLP-1R refers to ligands that selectively stimulate cAMP synthesis, decreasing β-arrestin recruitment, thus sustaining receptor signalling while minimizing its internalization and desensitization. However, growing evidence show that signalling bias is not an intrinsic and immutable property of ligands; it rather depends on biological assays format. The magnitude and direction of the observed bias can be altered by several factors, including levels of receptor expression, receptor reserve, relative abundance of G proteins, presence of GPCR kinases, β-arrestins, and other signalling partners. Often, it also depends on the read out and background of pharmacological responses. Indeed, the selected endpoint (e.g., cAMP accumulation, β-arrestin recruitment, extracellular signal-regulated kinase 1 and 2 phosphorylation, receptor internalization, or insulin secretion), assay design, signalling readout, signal amplification, temporal resolution are all variables that effectively bias GPCRs signalling. For instance, oxyntomodulin (OXM), a natural ligand of GLP-1R (see Section 3.1.3.1), and exendin-4 show signalling profiles that differ from the native ligands, promoting altered activation of canonical pathways, with β-arrestin recruitment observed in recombinant systems, and GLP-1R-expressing insulinoma cells (Wootten et al., 2016). Hence, the pharmacological profiles observed in recombinant cell systems with high receptor density may not accurately predict signalling behaviour in native tissues, where receptor abundance and downstream signalling networks differ markedly. These limitations highlight the need to evaluate biased agonism across multiple complementary experimental platforms and physiologically relevant models, to better define the link between *in vitro* signalling results and therapeutic outcomes (Hager et al., 2017).

Many studies demonstrated that cAMP biased agonism can promote better glycemic control and weight loss (Baumer-Harrison et al., 2026; Hao et al., 2022). Thus, functional selectivity is a promising approach to improve therapeutic efficacy while optimizing safety profiles, and is a compelling area of investigation for the development of novel anti-diabetics. Recent studies showed that mutations at the N-terminal “hydroxyl patch” of GLP-1 are useful in studying signalling bias (Wang P. et al., 2022). Four hydroxyl residues (i.e., Thr11, Ser14, Ser17, Ser18) within GLP-1 (7–36) were found to strongly influence intracellular Ca^2+^, ERK1/2, and β-arrestin-2 signalling, while determining weak action on cAMP stimulation or insulin secretion. Small modifications on these residues elicited diverse effects on intracellular signalling, creating new biased agonists. Last year, researches from Novo Nordisk Research Centre Indianapolis demonstrated in preclinical models how partial Gαprotein cAMP-biased GLP-1R agonists are able to confer superior weight lowering efficacy compared to balanced agonists (Douros et al., 2025). They supposed that biased agonism determines the retention at the plasma membrane of a larger receptor pool that can be continually reengaged by agonists circulating at pharmacological concentrations, thus enabling higher maximal efficacy. A small set of fatty-acylated GLP-1R agonists were synthesized starting from GLP-1 (7–36) template, profiled *in vitro* by calculating the ratio of cAMP:β-arrestin signalling (relative to the reference native GLP-1), and peptide **10**, featuring a C-18 diacid-γGlu-2xAEEA albumin binder on the C-terminal of Lys31 (Figure 12), emerged for its interesting profile. Compared to semaglutide, **10** partially activated GLP-1R (as determined by synthetic miniG_s_α and minG_q_α recruitment in dose-response and temporal measurement), promoting a partial guanosine triphosphate production. On the other hand, it retained maximal cAMP production albeit showing a reduced potency compared to semaglutide (EC_50_ values of 73.9 vs. 7.6 nm in GLP-1R - cAMP assessed in bioluminescence resonance energy transfer (BRET)-based assay). Moreover, it showed lower GLP-1R β-arrestin 2 recruitment and GLP-1R internalization compared to the native GLP-1 and semaglutide. When evaluated *in vivo*, peptide **10** exhibited high maximal efficacy with high maximal body weight reduction in diet-induced obesity mice, highlighting the importance to evaluate biased agonism in the search of candidate molecules as stronger predictor of the *in vivo* efficacy of GLP-1R agonists.

**FIGURE 12:**
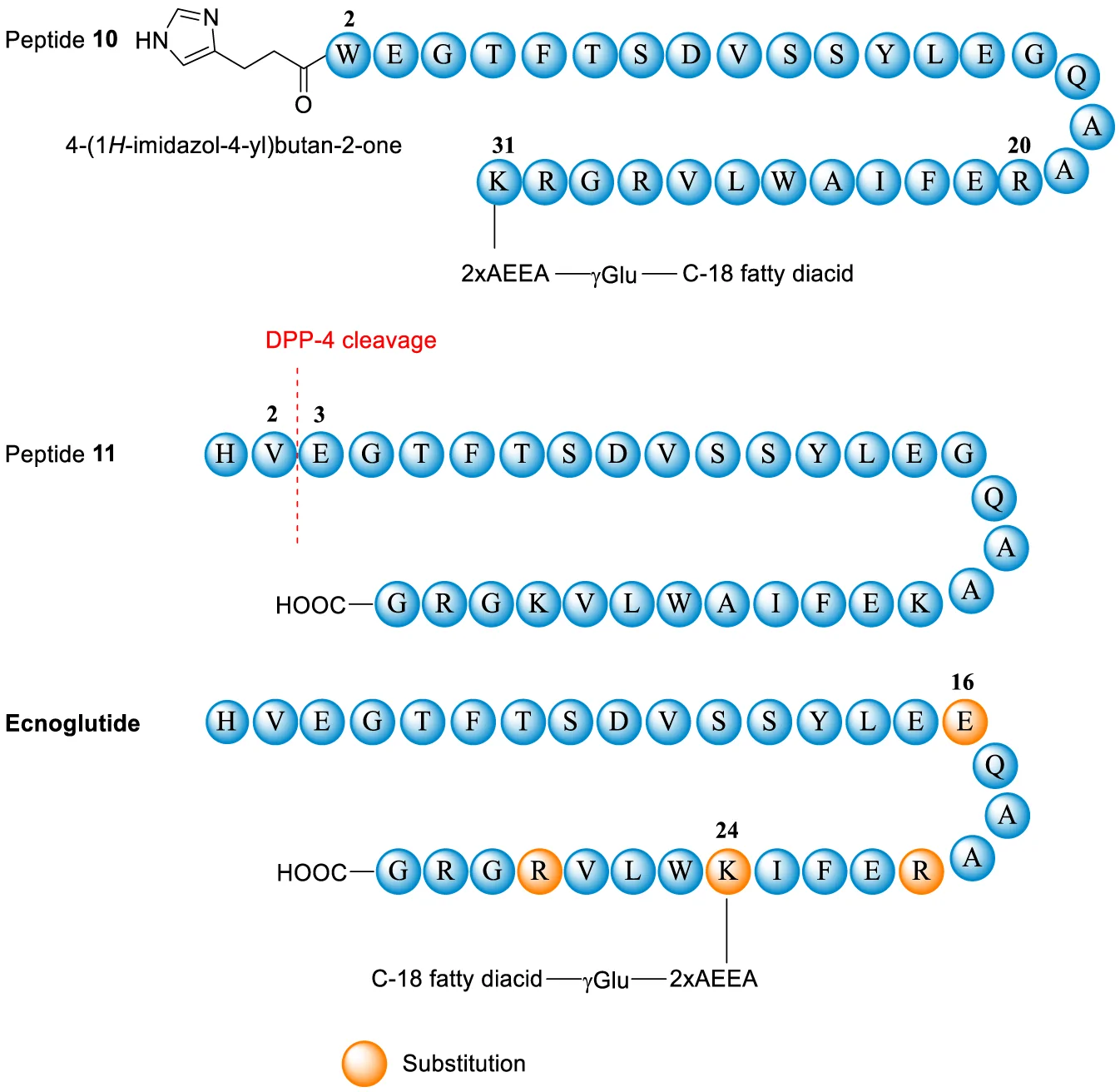
General structure of biased GLP-1R agonists peptide **10–11**, and ecnoglutide derived from GLP-1 (7–36).

Another example of biased GLP-1R agonist was developed by van der Velden and co-workers, which probed that replacement of the single Ala8 residue with Val8 at the N-terminus of GLP-1 (7–36)NH_2_ dramatically affected the ability of the peptide to internalize GLP-1R (van der Velden et al., 2021). It is known that class B1 GPCRs signalling is a two-stage binding process. In detail, the C-terminus of the ligand interacts with the extracellular domain of the receptor, which, in turn, helps the N-terminal region of the peptide to dock deep into the transmembrane domain triggering receptors conformational changes and leading to its activation. Due to the critical role of the N-terminus for receptor activation, the impact of a single amino acid substitution on ligand binding, receptor signalling, and internalization was evaluated. The introduction of a valine was expected to improve the stability, tolerability, PK, and efficacy profile. Compared to methyl side chain of alanine, the bulkier and more hydrophobic isopropyl side chain of valine was sought to modulate the protein’s surface properties, enzyme binding, and N-terminal processing. Moreover, this substitution is known to reduce N-terminal methionine excision and subsequent acetylation, thus protecting the peptide from a potential premature degradation, instability and misfolding. The newly obtained peptide **11** (Figure 12) maintained the same α-helical properties of the native ligand, and, *in vitro*, showed a similar profile compared to GLP-1 (7–36) when evaluating cAMP production (EC_50_ of 0.0316 nM vs. 0.0158 nM) and calcium mobilization (EC_50_ of 39.8 nM vs. 20 nM) using BRET-based assay; while, β-arrestin 2 recruitment diminished (EC_50_ of 39.8 nM vs. 15.8 nM). Moreover, receptor internalization capacity was heavily impaired upon **11** binding, with a 9.5-fold lower potency compared to GLP-1 (EC_50_ of 316 nM vs. 39.8 nM). Further studies confirmed that the compound greatly promoted cAMP synthesis, calcium mobilization, and recruitment of β-arrestin 2 over internalization. Although GLP-1 and **11** shared similar binding affinity constants (K_D_) (pK_D_ of 8.3 and 8.2, respectively), the potency observed for cAMP production was much higher, suggesting the presence of receptor reserve. *In vivo*, a decreased insulinotropic effect of peptide **11** was observed during acute administrations in a perfused rat pancreas model, overall demonstrating that subtle changes at the N-terminus of GLP-1 can dramatically influence hormone release.

Starting from these evidences, Guo and collaborators from Sciwind Biosciences designed and optimized a new series of GLP-1 analogues, culminating with the discovery of the long-acting biased agonist ecnoglutide (Figure 12) (Guo et al., 2023). The peptide presents an Ala2Val substitution, and a C-18 fatty diacid attached to the Lys24 through a γ-Glu-2xAEEA linker to prolong the half-life. *In vitro*, ecnoglutide was equipotent to semaglutide and showed signalling bias towards cAMP production (EC_50_ value of 2.322 ng/mL vs. EC_50_ of 2.437 ng/mL for semaglutide in cAMP induction assay), over β-arrestin recruitment and receptor internalization (EC_50_ values >10 vs.0.093 μM for semaglutide). In kinetic studies, ecnoglutide was found to strongly bind the hGLP-1R with higher affinity than semaglutide. In Sprague Dawley rats, it showed good PK properties (t_1/2_ = 11.7 h, T_max_ = 18 h, C_max_ = 387.3 nM, AUC_last_ = 16,274.8 h*nM), and, in line with the increase in activity of cAMP biased GLP agonists, it exhibited a remarkably higher efficacy (compared to semaglutide) in terms of blood glucose control and weight reduction. In phase 1 single- and multiple-ascending-dose studies involving healthy volunteers (n = 64), ecnoglutide was safe and well tolerated following once-weekly subcutaneous administration up to 6 weeks. An oral formulation of the compound was tested in phase 1 clinical trial (ClinicalTrials.gov, 2025k). The administration of oral ecnoglutide once-daily at 30 mg dose for 6 weeks determined a mean body weight reduction of 6.8% from baseline, compared to 0.9% for the placebo group. Moreover, the new formulation appeared well tolerated, with the insurgence of mild gastrointestinal side effects that are typical for other GLP-1R agonists. Results from a randomized, double-blind, placebo-controlled phase 3 trial to evaluate the efficacy and safety in adults with T2D, showed that once-weekly administration of the drug (0.6 mg or 1.2 mg) determined at week 24 the least changes in reduction of glycated hemoglobin (1.96%, with 0.6 mg dose, and 2.43% with ecnoglutide 1.2 mg, vs. 0.87% with placebo (clinicalTrials.gov, 2025h; Zhu et al., 2026). The drug received approval in China in early 2026 for T2D treatment, and is under review for chronic weight management (Chakravarthy et al., 2026; Scheen, 2025; He et al., 2025; Ji et al., 2025).

#### 3.1.3 Dual agonists targeting GLP-1R

GLP-1R agonists revolutionized the therapeutic landscape of obesity and T2D treatment with significant improvements in long-term blood glucose and body weight management while also conferring cardiovascular (Mullur et al., 2024) and antiatherosclerotic (Rakipovski et al., 2018) benefits. However, the use of GLP-1R agonists in monotherapy is restricted by variable efficacy that often plateaus, in addition to dose-dependent gastrointestinal adverse effects. For this reason, prompted by the clinical success of dual GLP-1R/GIPR agonists (e.g., tirzepatide), a growing interest has been directed in leveraging unimolecular multi-receptor targeting agonists to achieve improved metabolic benefits over single GLP-1R agonism.

##### 3.1.3.1 Dual-agonist peptides targeting GLP-1R and GCGR

A promising strategy to improve the efficacy of traditional therapy is to combine conventional GLP-1R agonists (e.g., glucagon) with gastrointestinal hormones like OXM (Figure 13) targeting pathways involved in energy metabolism and nutrients. OXM is a naturally occurring 37 amino acids peptide found in the colon, produced by the oxyntic cells, and able to activate both GLP-1R and GCGR. Its action promotes energy expenditure and reduces food intake, leading to weight loss in obese patients (Wynne et al., 2005). However, its clinical use has been limited by the extremely short half-life (∼10 min). Thus, the design of modified OXM analogues with increased potency toward GLP-1R and GCGR, and longer circulating half-life appealed the scientific community for the development of drugs for T2D and/or obesity management (Yang et al., 2020). Many studies demonstrated that the use of OXM-derived co-agonists was able to improve the duration of action of GLP-1R mono-agonists, by reducing target desensitization and downregulation, thereby maximizing weight loss and glycemic control (Pickford et al., 2021). Novel dual GLP-1R/GCGR agonists OXM-based were developed by Melander and co-workers, with peptide **12** (Figure 13) identified as the most promising candidate to treat obesity, T2D and NASH (Melander et al., 2024). Starting from OXM, (i) conjugation to a C-18-carbon fatty acid through a double γ-Glu-based linker at position 16 to improve albumin binding; (ii) and addition of a non-proteinogenic amino acid (i.e., Aib) at position 2 to prevent DPP-4 proteolytic cleavage were performed. These modifications improved the durability, stability, and potency of peptide **12** compared to the native ligand. *In vitro* studies showed that, while no cross activity was found against GIPR, peptide **12** exerted dual activity for GLP-1R and GCGR (EC_50_ of 417 nM and 3.02 nM, respectively, in GLP-1R mediated cAMP response element-driven -luciferase (CRE-Luc) production functional assay). In obese mice, peptide **12** promoted a dose-dependent reduction of the body weight (30 nmol/kg was the most effective dose). Treatment of diet-induced obese mice, NASH mice, and db/db mice with **12** decreased body weight by 35%, 40%, and 21% respectively, and, this effect seemed linked to increased energy expenditure rather than reduced food intake. Moreover, in NASH mice, the peptide emerged for the potent efficacy in lowering fasting blood glucose levels, accompanied by markedly reduced levels of insulin, while no effect was observed for the groups treated with semaglutide for 8 weeks. Finally, in db/db mice, the levels of glycated hemoglobin were lowered by 17.1%, and peptide **12** demonstrated a significant improvement of the overall β cell health, providing better glucose control. Furthermore, peptide **12** had hepatoprotective effect, exerting superior reduction in steatosis and fibrosis than semaglutide, meaning that, despite its less potency *in vitro* against GLP-1R and GCGR, the derivative demonstrated more efficacy *in vivo.* This effect was attributed to the intrinsic signalling bias toward GLP-1R, demonstrating the superior efficacy of the dual GLP-1R/GCGR agonism. Peptide **12** is a promising therapeutic candidate to treat obesity and related comorbidities, and represents a valuable option for NASH and liver health improvement.

**FIGURE 13:**
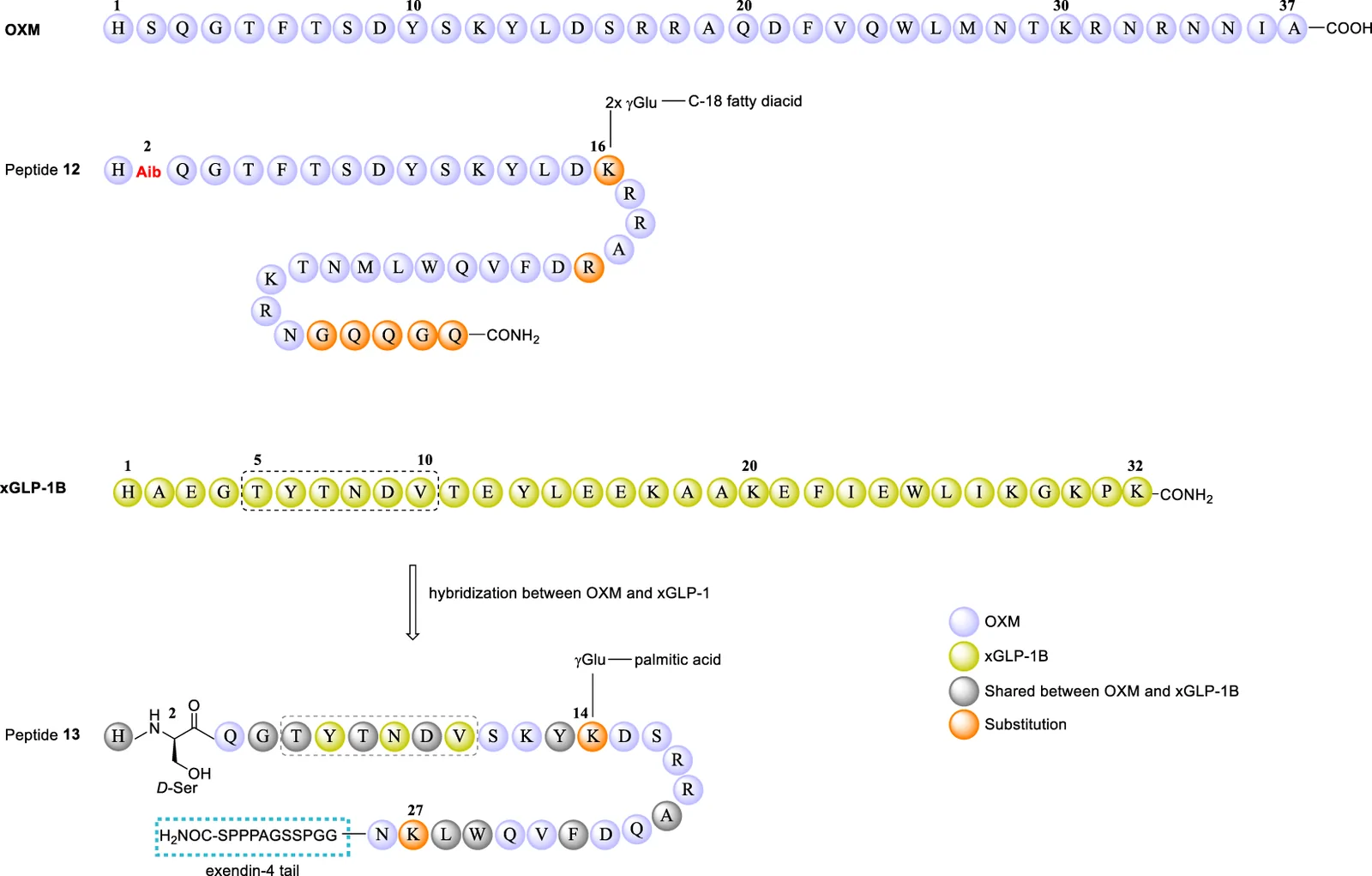
Amino acid sequence of OXM, xGLP-1B, and of dual GLP-1R/GCGR agonists **12**–**13**.

More OXM-based GLP-1R/GCGR dual agonists were disclosed by Zhang and collaborators (Zhang X. et al., 2023). OXM consists of the full glucagon sequence with an additional C-terminal octapeptide (Lys–Arg–Asn–Arg–Asn–Asn–Ile–Ala). The authors sought to expand the structure diversity of OXM-based dual agonists using the hybridization approach to combine the key structural elements of OXM, exendin-4 and xGLP-1B derived from *Xenopus* (x) laevis (Figure 13), together with fatty acid conjugation. The first step in the SAR exploration was the substitution of the N-terminal region of native OXM (Phe6-Thr-Ser-Asp-Tyr10) with the corresponding Thr5-Tyr–Thr–Asn–Asp–Val10 pentapeptide found in xGLP-1B, with the goal to modulate the potency. At the C-terminus, the exendin-4 tail was introduced. Further fatty acid modification with γGlu-palmitic acid side chain at position 14 afforded peptide **13** (Figure 13) with increased agonistic activity for both GLP-1R and GCGR (EC_50_ values of 0.024 nM (GLP-1R), and 0.082 nM (GCGR) in GLP-1R activation assay measured by cAMP accumulation), and resulting equipotent to liraglutide against GLP-1R (EC_50_ values of 0.018 nM), and two-fold more potent than cotadutide (EC_50_ values of 0.051 nM). The lead peptide **13** was profiled *in vivo*, and cotadutide was selected as a reference due to the structure similarity, as they share the same fatty acid side chain. In ICR mice, peptide **13** showed potent glucoregulatory effect, together with anorectic effect. In db/db mice, it exhibited a dose-independent hypoglycemic duration longer than the reference, probably due to its higher stability resulted from the replacement at position 2 of *L*-Serine with *D*-Serine to avoid rapid degradation by DPP-4. In diet-induced obesity mice, peptide **13** displayed prominent weight loss, glucoregulatory, and lipid regulation effects. Moreover, it enhanced markers of liver function leading to a significant weight loss, determined lowered hepatocellular vacuolation, reversed steatosis, and decreased liver total cholesterol and triglycerides levels, supporting its further development as a novel anti-NASH agent.

Pemvidutide (Figure 14) is another example of dual GLP-1R/GCGR agonist developed by Altimmune to treat obesity and NASH (Nestor et al., 2021), and its design was conceived based on earlier studies in which it was demonstrated that glycolipid surfactant conjugation was beneficial for a significant prolongation of peptides duration of action. To this purpose, covalent non-ionic glycolipid modifiers were explored to design balanced dual agonists suitable for weekly administration (Nestor and Wang, 2021). Starting from the structure of peptide **14** (Day et al., 2009), SAR exploration focused on (i) replacement of Met27 to prevent oxidation, and enhance the stability; (ii) removal of Asn28 to overcome synthetic limitations; (iii) evaluation of surfactants size and physicochemical properties. Moreover, (iv) the effect of the carbohydrate head at Lys20 toward the C-terminal of the side chain lactam linkage (1-*O*-alkyl β-D-glucopyranosiduronic acid, 1′-*O*-alkyl [β-(α-D-galactopyranosiduronic acid-(1→6′)]-D-glucoside, or 1-*O*-alkyl β-[β-D-glucopyranosiduronic acid-(1→4)]-*D*-glucopyranosiduronic acid), and (v) the alkyl chain length and structure were investigated to study the impact on solubility, potency, balance, and duration of action. In particular, the compound including the 1-*O*-[(17-carboxyheptadecyl)oxy] β-*D*-glucopyranosiduronic acid, and the glycolipid surfactant conjugation at Lys17 (Lys17GC18c), named pemvidutide, was designed to test the effect of mimicking the head group of fatty acids by incorporation of a carboxylic acid function at the terminus of the surfactant alkyl chain, to resemble semaglutide structure. The peptide emerged for its high and balanced potency toward both GLP-1R and GCGR (EC_50_ values of 39 pM and 42 pM, respectively, in GLP-1R activation assay measured by cAMP accumulation, with a selectivity ratio GCGR EC_50_/GLP-1R EC_50_ = 1) and, following subcutaneous administration, it showed prolonged PD profile in rats, with a mean resistance time of 21 h (vs. 15 h of semaglutide), T_max_ up to 8 h, a C_max_ of 76 ng/mL, and an AUC of 2,350 ngh/mL, supporting the suitability for once weekly dosing in humans. In diet-induced obesity mice, pemvidutide exhibited a more potent and longer-acting glucoregulatory effect in comparison to semaglutide, while achieving a more progressive maximum glucose-lowering potency. Moreover, animals treated with a low dose of pemvidutide (6 nmol/kg) displayed a similar feeding suppression at twice the molar equivalent dose, while determining greater weight loss. The lead compound also promoted a decrease of liver weight greater than semaglutide. Pemvidutide is currently advanced through phase 1 trials and phase 2 to evaluate its efficacy against obesity and NASH (clinicalTrials.gov, 2023b; clinicalTrials.gov, 2023a). Preliminary results from a 12-week randomized, double-blind, placebo-controlled study, evaluating the efficacy of pemvidutide in reducing the liver fat content, showed that the drug was well tolerated. Significant reductions in liver fat content, markers of hepatic inflammation, and body weight compared to placebo were observed (clinicalTrials.gov, 2023a). Unlike GLP-1R agonists, which elicit weight loss through centrally and peripherally mediated effects on appetite, the dual GLP-1R/GCGR agonist pemvidutide offers the advantage to directly act on the liver by stimulating fatty acid oxidation, and suppressing lipogenesis. This dual mechanism may provide a more effective strategy for liver fat content reduction than weight loss alone.

**FIGURE 14:**
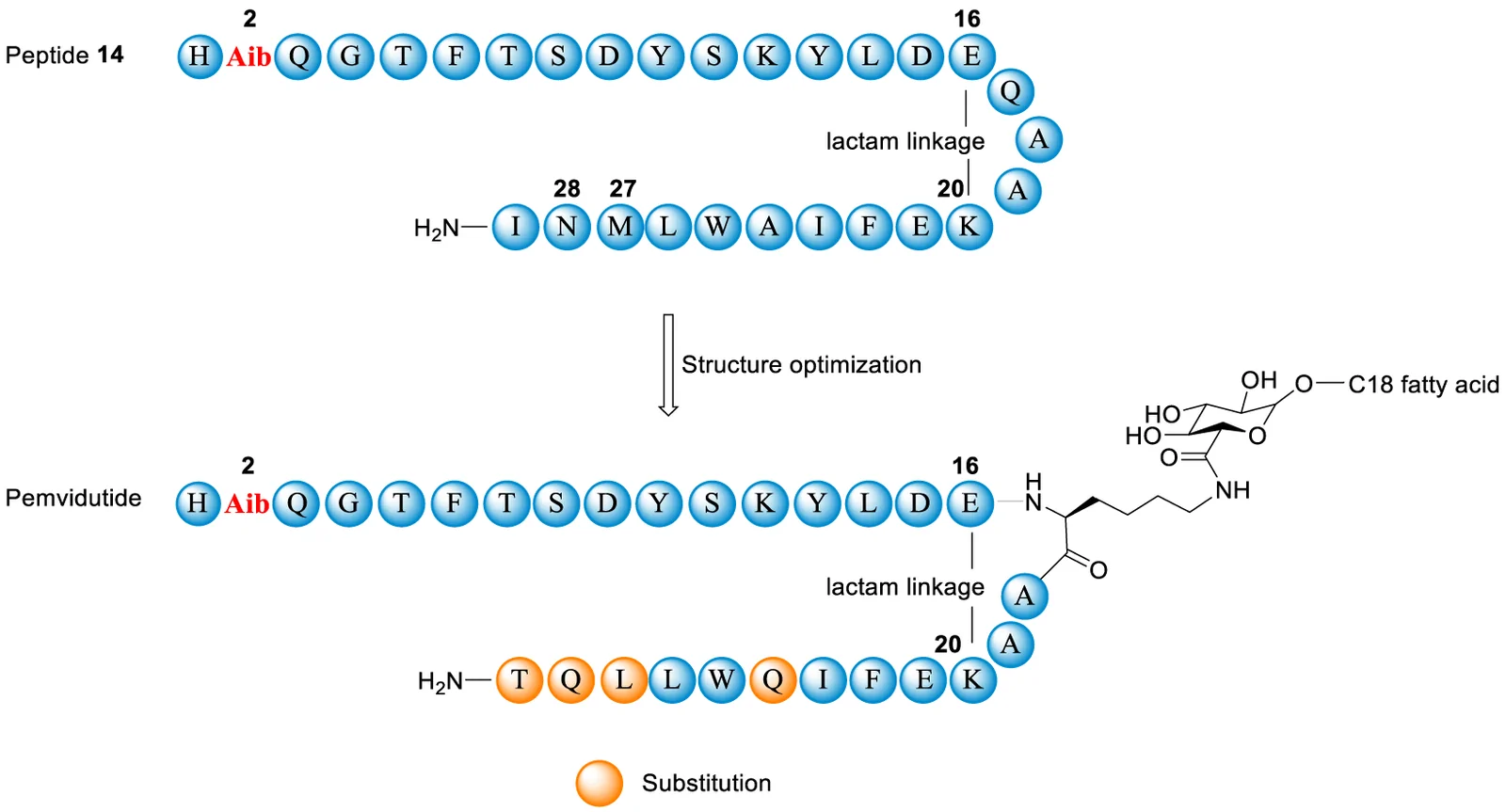
Rational design of pemvidutide starting from peptide **14**.

##### 3.1.3.2 Dual-agonists targeting GLP-1R and NPY2R

As above mentioned, the gut hormone PYY is released post-meal by enteroendocrine L-cells; it belongs to the pancreatic polypeptide (PP) family, and is able to delay gastric emptying, reduce appetite, decrease weight, and lower blood glucose (Chen et al., 2023). PYY_3-36_ peptide originates from truncation by DPP-4 of PYY_1-36_ N-terminus, and is the main circulatory form; its concentration levels rise after feeding. PYY_3− 36_ presents high binding selectivity for NPY2R over NPY1R, NPY4R, and NPY5R, and plays a key role in developing and maintaining obesity, as its levels are usually lower in obese individuals (Poulsen et al., 2021). PYY analogues generally suffer from short half-live and gastrointestinal side effects. Indeed, the co-administration of GLP-1R agonists has been exploited to suppress appetite and achieve significant reduction of food intake (Østergaard et al., 2021; Milliken et al., 2021).

Liu and co-workers disclosed new dual-agonists targeting GLP-1R and NPY2R, as promising agents to manage glucose homeostasis and body weight regulation in obese patients (Liu J. et al., 2025). Three structurally unrelated GLP-1R agonists, i.e., (i) exendin-4, (ii) semaglutide, and (iii) the long-acting GLP-1 analogue **15** (i.e., an amphibian agonist derived from bGLP-1, respectively, Figure 15) were selected as the starting template for optimization studies. The novel dual agents were designed by connecting this peptide to PYY-2, being more selective toward NPY2R compared to PYY_3-36_ (Figure 15) via the OEG linker (Lear et al., 2020; Yang et al., 2022a). The left side of **15** sequence was modified with a cysteine residue at the C-terminus, while at the N-terminus of the right PYY-2 segment was inserted a 3- and 6-maleimide propionic acid. Moreover, four OEG linkers (i.e., 2xAEEA, 4xAEEA, 6xAEEA, and 8xAEEA) were introduced to evaluate the impact of the length on the bioactivity, and SAR exploration identified positions 17 o 20 of the left-side segment as the most suitable for fatty acid modifications. Preliminary data showed that the introduction of thiol-maleimide linkers compromised the *in vivo* stability of the peptides. Thus, a new design strategy evaluated the removal of the cysteine and maleimide propionic acid linkers, without significantly affecting the *in vitro* activity of the dual GLP-1R/NPY2R agonist. Moreover, the insertion of OEG linkers seemed to not directly participate in the interactions between GLP-1R and NPY2R. Rather, their effects on the activity were likely related to the linker length, which could modulate the spatial conformation of the molecule. Peptide **16** emerged as the lead candidate for its higher agonistic activity (GLP-1R EC_50_ value of 0.0015 nM, NPY2R EC_50_ value of 69 nM in GLP-1R and NPY2R activation assays). This dual agonist was structurally composed by a bGLP-1 analogue on the left-side segment, a central linker of four OEG units, PYY-2 at the right-side segment, and a Gly-Gly-Ser-Gly-Ser-Gly-C-18 fatty diacid side chain at Lys17 residue. In C57BL/6J mice and Sprague-Dawley rats, the compound suppressed fasting behavior with no significant gastrointestinal side effects. In diet-induced obesity mice, after a single subcutaneous dose of 100 nmol/kg, peptide **16** showed high stability, with half-life of approximately 13.6 h (vs. 14.7 h for semaglutide). Moreover, it significantly enhanced body weight loss (driven by reduction of adipose tissue with minimal impact on lean mass) at both low and high doses (2 and 10 nmol/kg), and the food intake was markedly lower compared to groups treated with semaglutide (P < 0.001). The compound also decreased serum cholesterol and triglycerides, and reduced hepatic lipid content, while significantly improving glucose tolerance, confirming the potential of dual-targeting conjugates as a promising strategy to develop glucoregulatory treatments, providing high weight loss accompanied by less gastrointestinal side effects.

**FIGURE 15:**
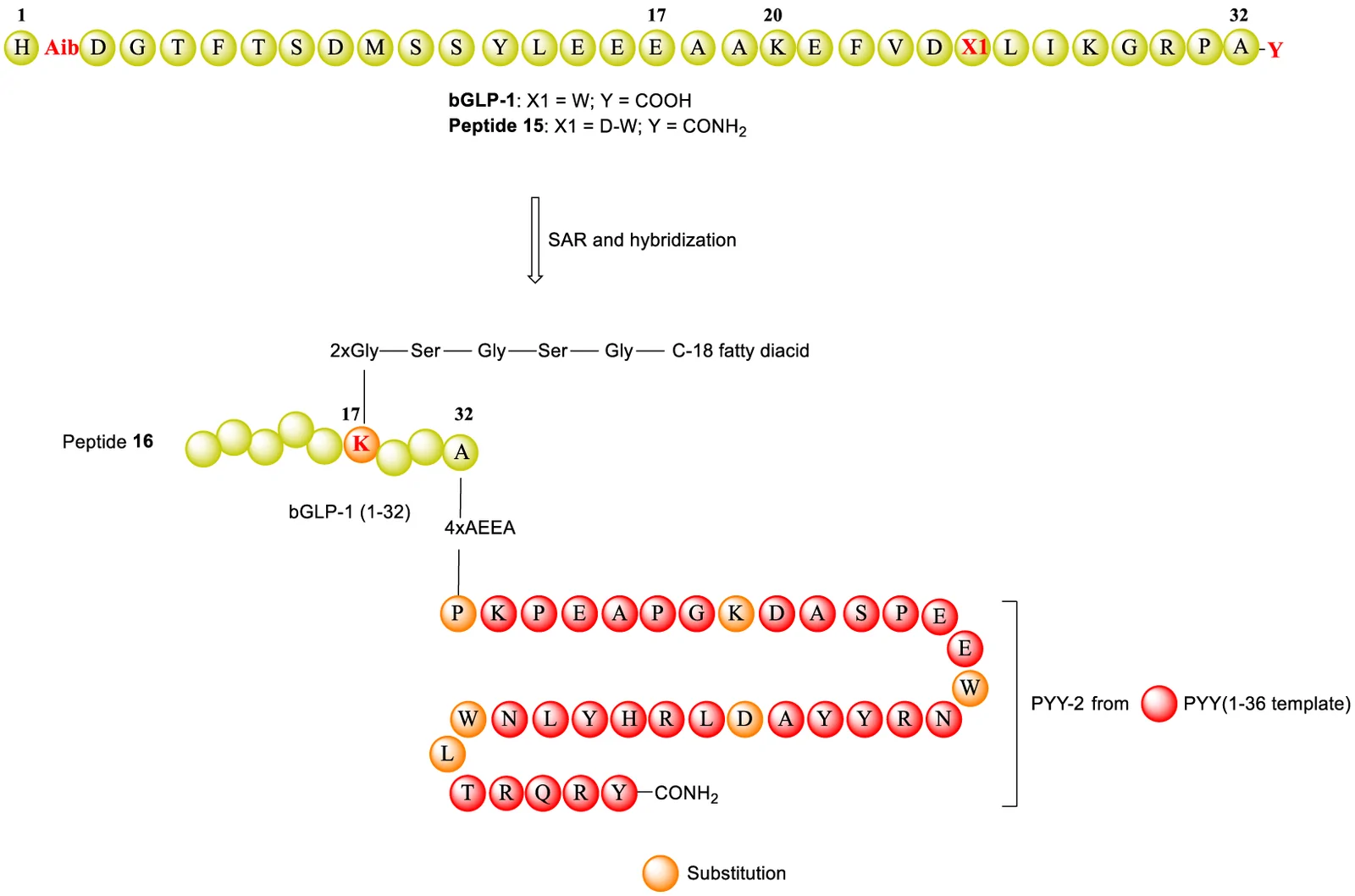
Amino acid sequences of bGLP-1 and peptide **15**, and SAR studies toward the novel hybrid peptide **16**.

Xu and co-workers disclosed a novel potent and long-acting GLP-1R/NPY2R dual agonist (compound **18**, Figure 16) to potentially use to treat T2D and obesity (Xu et al., 2023). The peptide was obtained via optimization studies, to overcome the instability of the cysteine and 3-maleimidopropionic acid linker of precursor **17** (Yang et al., 2022a). Novel dual agonists were designed by directly conjugating GLP-1 and PYY analogues through the OEG linker. Several albumin binders, including 2xAEEA-γGlu-C-18 fatty diacid (used for semaglutide), 2xAEEA-γGlu-C-20 fatty diacid (used for tirzepatide), γGlu-γGlu-Ser-Glu-Ser-γGlu-γGlu-C-18 fatty diacid (Simonsen et al., 2022), Gly-Gly-Ser-Gly-Ser-Gly-C-18 fatty diacid (structurally similar to the fatty acid side chain used in survodutide) (Zimmermann et al., 2022), and εLys-εLys-γGlu-C-20 fatty diacid (used for peptide **8**, Figure 10) were investigated (Knerr et al., 2022). Compound **18** with a 2xεLys-γGlu-C-20 fatty diacid chain showed equipotent GLP-1 and NPY2R activation potency (EC_50_ values of 0.027 nM (GLP-1R), and 1.8 nM (NPY2R) in GLP-1R and NPY2R activation assays), and resulted selective toward NPY2R over other receptor subtypes. The peptide exerted satisfying glucose-lowering effects and reduced food intake (compared to the reference liraglutide). In Kunming and diet-induced obesity mice, peptide **18** elicited hypoglycemic effect similar to semaglutide, while demonstrating a greater inhibition on food intake, with no vomiting and nausea. The absence of any significant adverse effects could be explained with the imbalanced potency observed in activating GLP-1R and NPY2R. PK studies supported once-weekly dosing after subcutaneous injection. In a 3-week study on diet-induced obesity mice, peptide **18** exerted greater effect than semaglutide on reducing food intake (P < 0.001), consequently leading to greater reduction in body weight (29.8% vs. 16.8%, respectively). Moreover, blood glucose levels decreased gradually. Finally, the observed weight loss induced by peptide **18** was linked to improvements in liver metabolism and histology, through the reduction of hepatocellular vacuolation, and reversing steatosis, thus demonstrating the potential of long-acting GLP-1R/NPY2R dual agonists as anti-obesity, anti-diabetic, lipid regulator, and antisteatotic agents.

**FIGURE 16:**
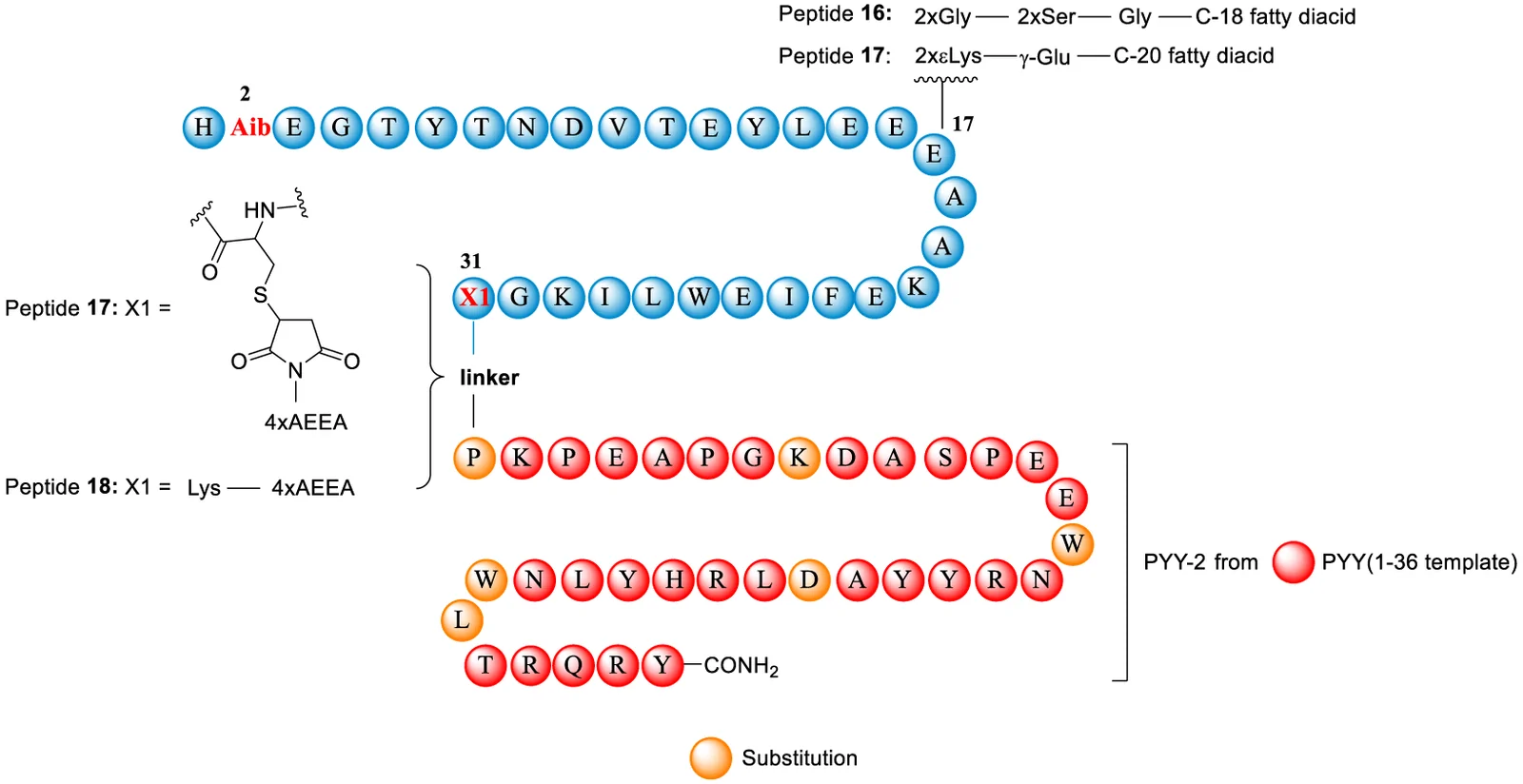
General structure of the optimized long-acting GLP-1R/NPY2R dual agonist **18** derived from optimization of peptide **17**.

##### 3.1.3.3 Dual-agonist peptides targeting GLP-1R and GIPR

Seeking to discover novel long-acting GLP-1 agonists (Sang et al., 2021), Shi *et al.* described new α/sulfono-γ-AApeptide (Shi et al., 2023), (i.e., a class of peptidomimetic foldamer derived from the backbone of the chiral peptide nucleic acid) with high resistance to proteolytic digestion, improved bioavailability, chemo-diversity, and cell permeability compared to the canonical α-peptides (Teng et al., 2016). The sulfono-γ-AApeptides subclass (Figure 17a) gained high interest for protein recognition and modulation of protein‒protein interactions (Sang et al., 2020b), due to the helical folding conformations, depending on intramolecular hydrogen bonding, and intrinsic turn-forming curvature of tertiary sulfonamide moieties on the molecular backbone (Sang et al., 2020a). Hybrids were obtained by replacement of amino acid residues at both the C- and N-terminus of GLP-1 with sulfono-γ-AA building blocks; moreover, to increase the metabolic stability, a fatty acid tail was attached at positions 17, 20 and 24 of selected peptide backbones. The newly obtained conjugates retained the right-handed helical conformation (Lau et al., 2015), with **19** (bearing Aib2, and a tail at the position 17, Figure 17b) emerging as lead candidate for its good potency (EC_50_ value of 0.27 nmol/L in GLP-1R mediated CRE-Luc production functional assay) and remarkable higher stability in human serum up to 24 h (compared to the native GLP-1). When evaluated *in vivo* in BALB/c female mice, compound **19** displayed prolonged half-life (approx. 100 min); however, it showed a delayed value of time of maximal concentration after sub cutaneous administration (T_max=_2.5 h), probably due to the injection site depot effect of the fatty acid modified peptide. Peptide **19** also had a long-lasting glucose lowering pharmacodynamic effect, highlighting the strong potential of α/sulfono-γ-AA-peptides in preserving the α-helical structure, and representing a valuable approach to improve peptides stability without compromising the biological activity.

**FIGURE 17:**
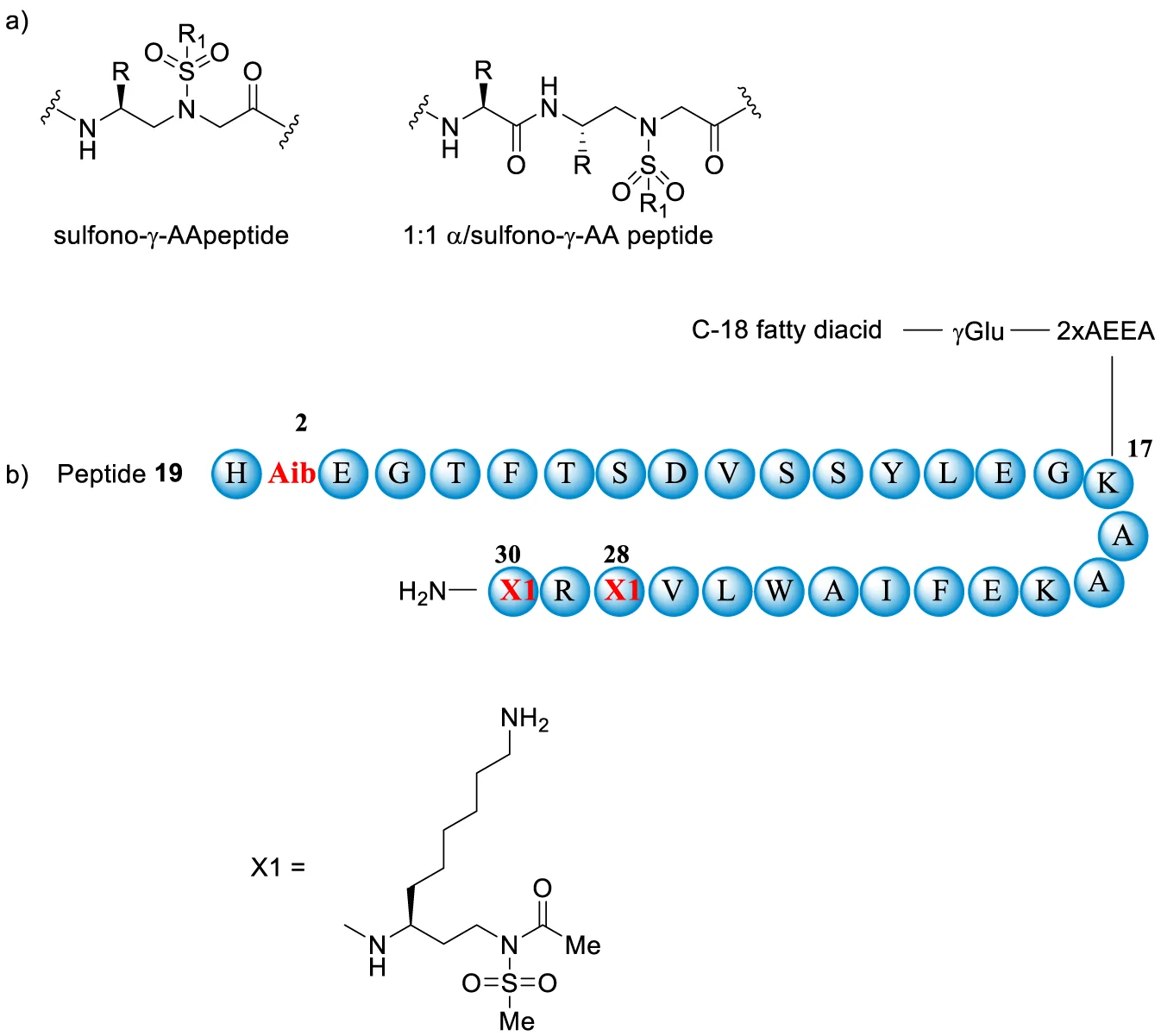
**(a)** Chemical structures of sulfono-γ-AApeptide and 1:1 α/L-sulfono-γ-AApeptide building blocks; **(b)** General structure of peptide **19**.

Another promising strategy aimed at enhancing resistance to DPP-4 hydrolysis was developed by Dinsmore *et al.*, which described new potent aza-peptide GLP-1R/GIPR dual agonists (Dinsmore et al., 2024). Aza-amino acids were used as bio-isosteric replacement of canonical α-amino acids in the backbone of both GLP-1 and selected dual GLP-1R/GIPR agonists, enabling structural backbone modifications of the ligand–receptor complex. Changes from the tetrahedral α-carbon to the trivalent substituted nitrogen yielded a dynamic chirality, thus expanding the three-dimensionality of the obtained peptides. It is worth to mention that, while Aib shows a preference for α-helical structures, aza-amino acids adopt β-turn conformations that may trigger ‘bias’ in the signalling pathways linked to GPCR activation (see Section 3.1.2). Aza-glutamic acid, aza-alanine, aza-glycine and aza-proline (Figure 18a) were selected for the substitution of the residue adjacent to the scissile bond of the ligands template. Derivative **20**, which was the structurally closest analogue of semaglutide, with Aib2AzaA and Aib20Ala modifications, and a lipidated residue at the C-terminus (Figure 18b), resulted the most potent with higher agonistic activity compared to native ligand toward both GLP-1R (EC_50_ values of 0.8 pM vs. 1.6 pM) and GIPR (EC_50_ values of 2.4 pM vs. 6.3 pM) measured by GLP-1R mediated CRE-Luc production functional assay. Moreover, it showed high stability toward DPP-4 proteolysis, with no difference in potency when incubated with the protease or with the vehicle, probably suggesting (i) a kinetically slow degradation; (ii) an unaligned scissile bond due to structural differences conferred by the aza-substitution; or (iii) a conformational change preventing the binding within the enzyme’s active site. These preliminary results encourage further investigation of aza-peptides as new templates for the development of promising protease-resistant drugs to treat T2D and obesity.

**FIGURE 18:**
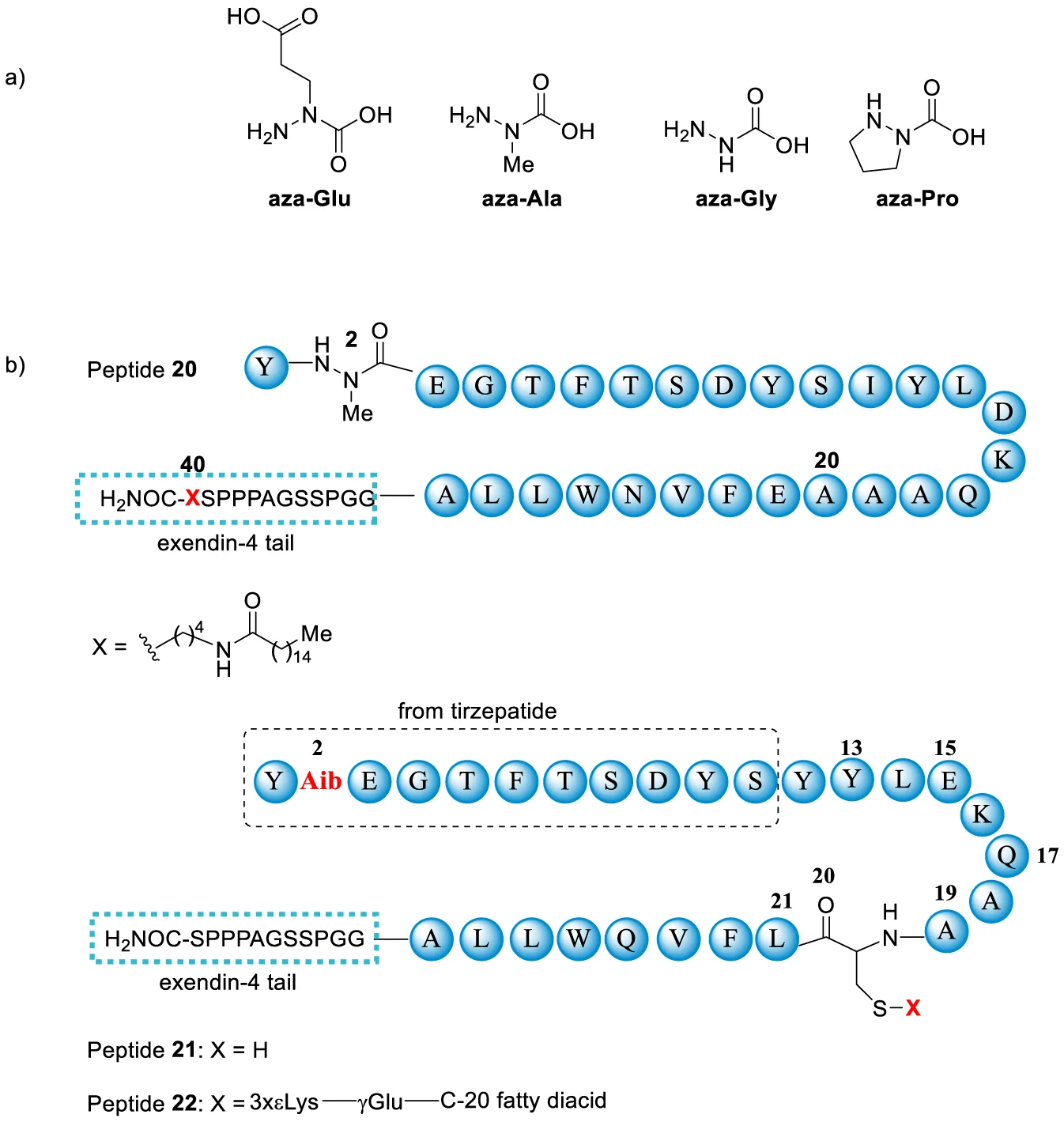
**(a)** Schematic representation of aza-glutamic acid, aza-alanine, aza-glycine and aza-proline; **(b)** General structure of dual GLP-1R/GIPR agonists **20–22**.

In the effort to optimize the structure of tirzepatide (Manchanda et al., 2025), Dong and co-workers disclosed a novel long-acting dual GLP-1R/GIPR agonist, with improved activity toward GLP-1R (Dong et al., 2024). As depicted in Figure 18, for the design, positions 1–11 of tirzepatide sequence were retained; Aib residue was inserted at position 2 to protect the new peptides against DPP-4 cleavage and inactivation; at the C-terminus, the 4-exendin tail was introduced to promote peptide helicity and structural stability via intramolecular interactions; moreover, GLP-1 amino acids were introduced at residues 13, 15, 17, 19, and 21. Finally, cysteine residues were inserted either at position 13, 16, 20, 21, 24, or 27 as anchor points for subsequent modification with albumin-binding side-chains. Peptide **21** emerged *in vitro* as the most promising of this first set of derivatives, and exhibited improved activity toward GLP-1R (EC_50_ values of 0.29 nM), but less potency against GIPR in cAMP dose response assays [EC_50_ values of 0.60 nM; vs. tirzepatide EC_50_ values of 0.03 nM (GIPR), 0.77 nM (GLP-1R)], and was used for subsequent modification with fatty acids. Indeed, a second group of analogues was obtained by conjugation to albumin binding moieties (including long alkyl chains of C-18 and C-20 diacid) using AEEA-2xγGlu-Lys, γGlu-2xAEEA-Lys, and γGlu-2xεLys-Lys as hydrophilic linkers. Peptide **22** (Figure 18), with a C-20 diacid-γGlu-3xεLys group conjugated to the sulfhydryl group of the cysteine side chain at position 20, showed high potency toward GLP-1R (EC_50_ values of 0.18 nM) and slightly less activity toward GIPR (EC_50_ values of 0.06 nM). *In vivo* chronic studies in diet induced obese mice showed that the compound was able to reduce body weight by 21%; while, in diabetic db/db mice, chronic dosing of the peptide appeared to have a more potent hypoglycemic effect than semaglutide up to day 31. Moreover, in comparison with tirzepatide, peptide **22** induced a stronger weight loss confirming is suitability for once-weekly administration in humans, and suggesting its potential as efficient agent to treat T2D and obesity.

##### 3.1.3.4 Dual agonists targeting GLP-1R and GDF-15 pathways

GDF-15 is a cell activation and stress response cytokine of the glial cell line-derived neurotrophic factor family and belongs to the TGF-β superfamily. Recent findings identified the molecule as key regulator of appetite homeostasis and established its therapeutic potential as effective anti-obesity agent. Indeed, dual targeting of GLP-1R and GDF-15 pathways emerged as highly promising to treat obesity, with minimal impact on skeletal muscle mass (Zhang et al., 2024; Ghidewon et al., 2022). A couple of years ago, Zhang and collaborators reported the design and synthesis of novel long-acting GLP-1/GDF15 fusion proteins as valuable poly-pharmaceutical tool in metabolic drug discovery (Zhang Y. et al., 2023).

The new hybrids were designed by fusing the C-terminus of GLP-1 variant with the N-terminus of GDF-15 analog via a flexible poly-peptide linker (Figure 19). The shift of the C-18 fatty diacid generated a new set of compounds with different ratio in GLP-1R/GDF-15 potencies. Fatty acid conjugation within or close to GLP-1 moiety in the linker region reduced GLP-1R activity, with minimal impact on GDF-15 pathway. Similarly, insertion of the C-18 diacid in the linker region adjacent to GDF-15 sequence dramatically affected GDF-15 signalling, while preserving GLP-1R activity. In fact, the middle region of the poly-peptide linker appeared the most optimal position to maintain both GLP-1 and GDF-15 agonism. The fusion protein conjugate with a C-18 fatty diacid at position 64and with GDF-15 variant emerged as the most promising balanced dual agonist [EC_50_ values of 0.05 nM (GLP-1R) in GLP-1R mediated CRE-Luc production functional assay, and 50 nM (GDF-15)]. In obese cynomolgus monkeys (the first-in-class animal model to predict compound efficacy in humans), the compound showed remarkable PK properties (T_max_ (h) = 16.0, C_max_ (nmol/L) = 120.4, t_1/2_ (h) = 15.9, terminal t_1/2_ (h) = 41.0, AUC_0–24h_ (hr*nmol/L) = 2,163.7), suggesting the opportunity for at minimum once-weekly dosing. Further *in vivo* assessment in diet-induced obese mice, showed that the lead candidate exerted a dose-dependent weight loss, normalization of hyperglycemia, improvements in circulating insulin levels, and decrease in plasma and liver triglycerides, and cholesterol. While, in db/db mice, the compound demonstrated high efficacy in reducing body weight, food intake, and fasting glucose, overall empowering that balanced GLP-1R/GDF15 dual agonism may represent an efficacious approach to treat obesity and associated morbidities.

**FIGURE 19:**
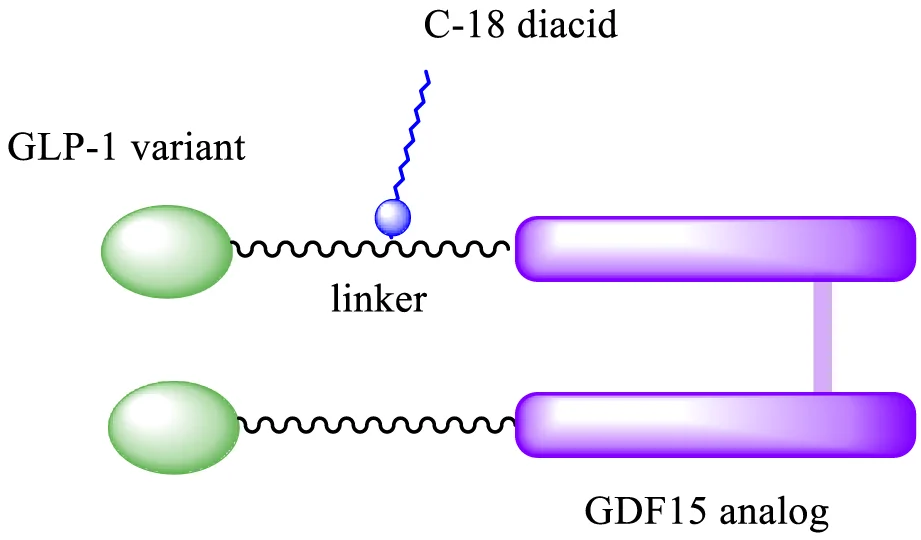
Schematic representation of the GLP-1/GDF15 fusion protein.

##### 3.1.3.5 Dual agonists targeting GLP-1R and cholecystokinin receptors

Cholecystokinin receptor −1 is primarily found in the peripheral nervous system and gastrointestinal tract, and its activation is responsible of appetite regulation, gastric emptying, pancreatic enzyme secretion, and gallbladder contraction. Many research studies showed the synergistic effect of co-administration of a cholecystokinin receptor-1 agonist with GLP-1R on metabolic improvements in obese rodents (Roth et al., 2021). Yang and co-workers developed novel GLP-1R/cholecystokinin receptor-1 co-agonists as potential antidiabetic agents (Yang et al., 2022b). The new scaffold was constructed by linking the N-terminus of either the native GLP-1 to the C-terminus of a cholecystokinin receptor-1 selective agonist (e.g., peptide **23**, EC_50_ value of 0.21 nM, Figure 20) via an OEG linker (AEEA also found in peptide **23**) (Christoffersen et al., 2020). A fatty acid was inserted at the side chain of Lys20 or Lys28 to achieve an optimal PK profile. Peptides **24** (EC_50_ values of 0.15 nM (GLP-1R) in GLP-1R activation assay, 1.25 nM (cholecystokinin receptor-1) in cholecystokinin receptor-1 activation assay measured by the phosphorylated extacellular signal-regulated kinase induction) and **25** [EC_50_ values of 0.074 nM (GLP-1R), 0.83 nM (cholecystokinin receptor-1)] emerged for their potent and selective *in vitro* activities and were selected for further *in vivo* evaluation. In Sprague–Dawley rats, following intravenous administration, the half-lives of **24–25** were 6.3 and 9.1 h, respectively. The enhanced stability of peptide **25** was attributed to the Aib2 mutation of xGLP-1 sequence. In normal ICR mice, both peptides showed better glucose tolerance than the native peptide **22**, and lowered the blood glucose concentrations after load. In db/db mice, both derivatives showed better glucoregulatory, insulinotropic efficacy, and pancreas function restoration compared to peptide **23** and semaglutide references. Moreover, in a 3-week sustained dosing study in diet-induced obese mice, **24** and **25** elicited greater reduction in weight, food intake, and fat mass, determining also improved glycemic control, insulin sensitivity, and lipid regulation (compared to semaglutide and peptide **23**).

**FIGURE 20:**
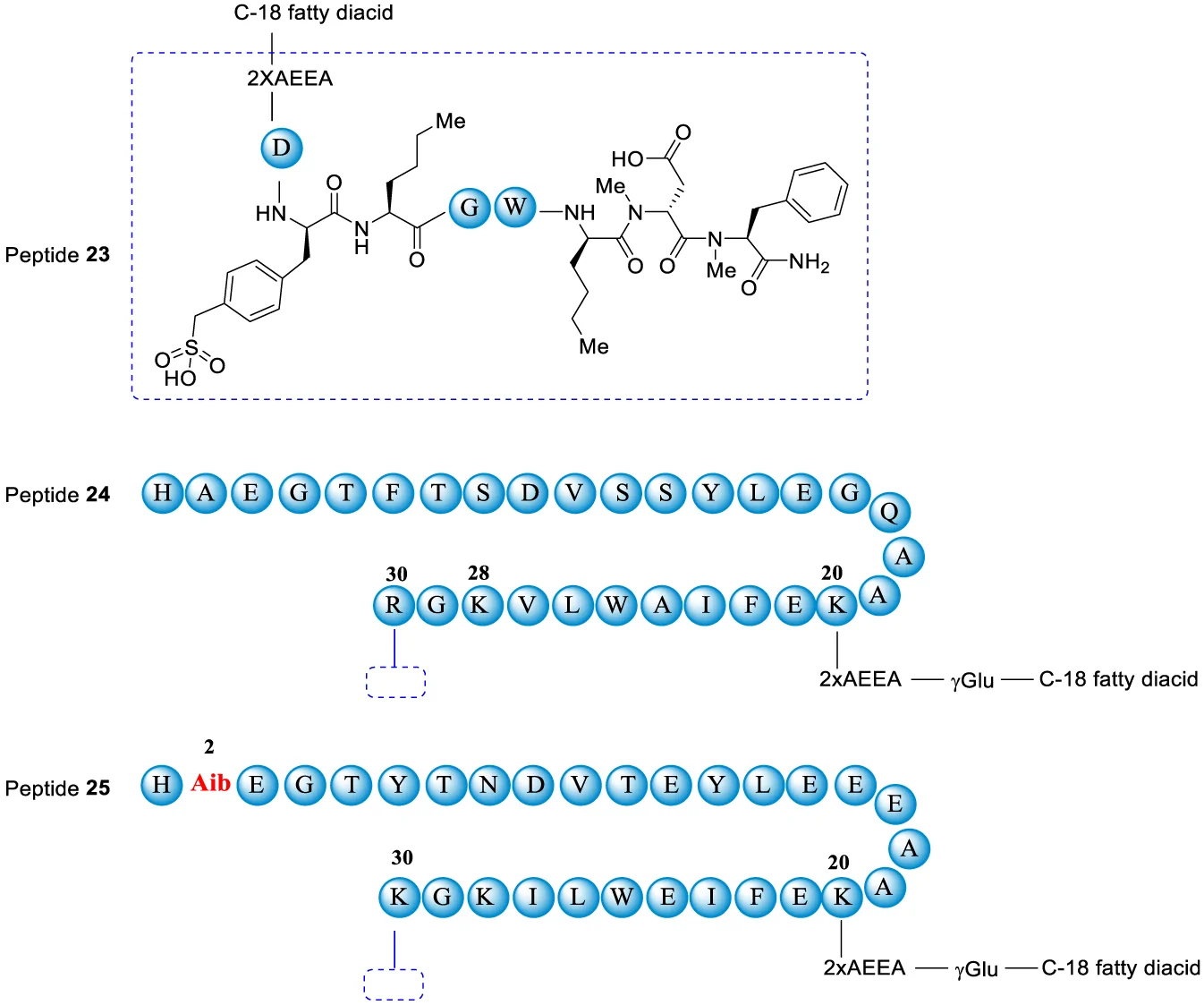
General structure of peptides **24–25** derived from the cholecystokinin receptor-1 selective agonist peptide **23**.

The same research group worked on the development of dual xGLP-1/cholecystokinin 2 receptor co-agonists (Zhou et al., 2022). Cholecystokinin 2 receptor is located in the central nervous system and stomach, and is responsible to regulate anxiety, pain perception, and gastric acid secretion. Cholecystokinin and gastrin (Tyr-Gly-Trp-Leu-Asp-Phe-NH_2_) are its endogenous ligands. Many studies demonstrated that co-administration of gastrin and epidermal growth factor, exendin-4, or GLP-1 in rodent models of diabetes restored normoglycemia, and was linked to enhanced β cell growth and differentiation, and preservation of β cell mass. Guided by sequence analysis, key structural features of two cholecystokinin 2 receptor agonist moieties were engineered into the xGLP-1, yielding hybrid peptides with potent dual receptor activity. Several fatty acid albumin binding motifs were inserted to increase the PK profile. Moreover, the length of the linker between the two moieties and the fatty acid modification site were studied. Peptides **26–27** (Figure 21) emerged for their promising *in vitro* bioactivities (EC_50_ values of 0.040 nM, and 0.032 nM (GLP-1R) in GLP-1R activation assay, >100 nM (cholecystokinin receptor-1), 6 nM, and 6.8 nM (cholecystokinin receptor-2), respectively in cholecystokinin receptor-1/-2 activation assay measured by the phosphorylated extracellular signal-regulated kinase induction), and, *in vivo*, both peptides determined remarkable appetite-suppressive effects over a period of 48 h, with improved glucose tolerance, and reduced glucose excursion. After intravenous injection, peptides **26–27** showed half-life comparable to semaglutide (t_1/2_ = ∼9.4, ∼8.8 h, vs. ∼8–9 h, respectively), suggesting the potential use once a week in humans. Furthermore, in a 5-week sustained dosing study in db/db mice, the compounds showed enhanced effects on pancreas function restoration and β cell proliferating action (compared to semaglutide). Finally, a greater reverse of steatosis was observed after 3 h treatment with **27**, supporting further investigation of dual GLP-1R/cholecystokinin 2 receptor agonists to develop therapeutic candidates for diabetes and related metabolic diseases.

**FIGURE 21:**
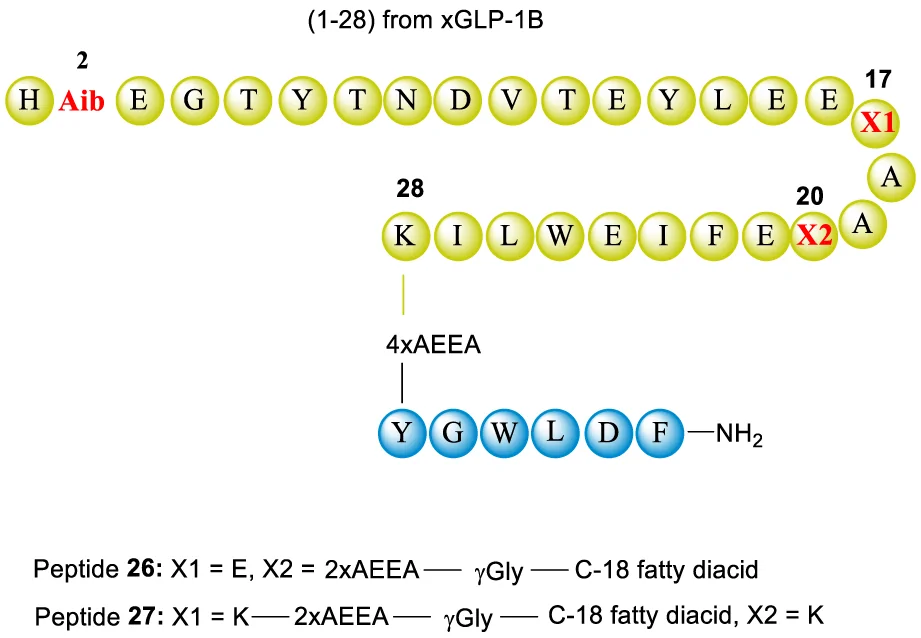
General structure of xGLP-1R/cholecystokinin 2 receptor co-agonists **26–27** starting from xGLP-1 template.

##### 3.1.3.6 Dual GLP-1R/secretin receptor (SCTR) agonists

In silico studies support medicinal chemistry programs by accelerating the process and enabling the exploration of wide chemical spaces that would be impractical to investigate experimentally (Wong A. et al., 2025). However, their application on peptide-based drug discovery for GPCRs has been historically curbed by small datasets. Structures were limited to the extracellular domain, as full-length GPCRs were unstable in conventional detergents. The breakthrough came with recent cryogenic electron microscopy structures, revealing the binding mode of ligands, and enabling accurate modeling of both class A and B GPCRs (Krumm and Roth, 2020; Mizera and Latek, 2021; Underwood et al., 2010; Bhushan et al., 2022). In 2024, Nielsen and collaborators developed a new platform called streaMLine to systematically explore the backbone of secretin, and to design, and synthesize potent, selective, and long-acting GLP-1R agonists, with advanced physicochemical properties (Nielsen et al., 2024). Secretin is a 27-amino acid hormone belonging to the glucagon superfamily (Figure 22). It modulates water homeostasis and bicarbonate secretion from the exocrine pancreas, and inhibits gastric acid secretion by stimulating SCTR. Since the native secretin peptide does not possess activity against GLP-1R, a first dual GLP-1R/SCTR agonist was developed introducing GLP-1 residues into the secretin native ligand (as depicted in the general structure of Figure 22), and the obtained peptide **28** was used as starting point for further optimization. Non-conserved residues of secretin template (e.g., position 2–3, 9–10, 12–14, and 17–25) were replaced by conserved amino acids of GLP-1, either one at a time or in combination, to afford a library of new peptides, which were screened against GLP-1R and SCTR. SAR exploration showed that amino acid at positions 2, 9, 18, and 22 were critical to improve GLP-1R potency; while, positions 3, 9, 10, 14, and 19 were essential for abolishing SCTR potency or enhancing GLP-1R selectivity. Mutation of amino acid at positions 12, 14, 18, 19, 21, 23, and 25 increased the tendency of peptides to form fibrils with subsequent reduced solubility. Moreover, insertion of Aib2 was sought to prevent DPP-4 proteolytic cleavage; while, Glu3 mutation prevented the isomerization of the aspartic acid residue in native secretin without affecting the potency. In addition, fatty acid conjugation was investigated to reduce renal clearance and enzymatic degradation. Peptide **29** was identified as the most promising candidate (EC_50_ values of 0.018 nM (hGLP-1R) in GLP-1R activation assay, and 190 nM (hSCTR) in SCTR activation assay measured by cAMP accumulation), with high chemical stability at neutral pH, minimal GLP-1R potency loss (less than 10-fold) compared to native GLP-1, but with a 10,000-fold selectivity ratio (GLP-1R over SCTR). Moreover, it was highly selective vs. GLP-1R with no activation of GIPR, GLP-2R, and GCGR (even when tested at very high concentrations). *In vivo*, peptide **29** had prolonged half-life of 22 h after intravenous dosing in rats, compatible with once-weekly dosing, and showed a marked reduction in food intake, and robust body weight loss comparable to the reference semaglutide, highlighting the potential of quantitative SAR as a valuable tool to accelerate the development of new peptide drug candidates.

**FIGURE 22:**
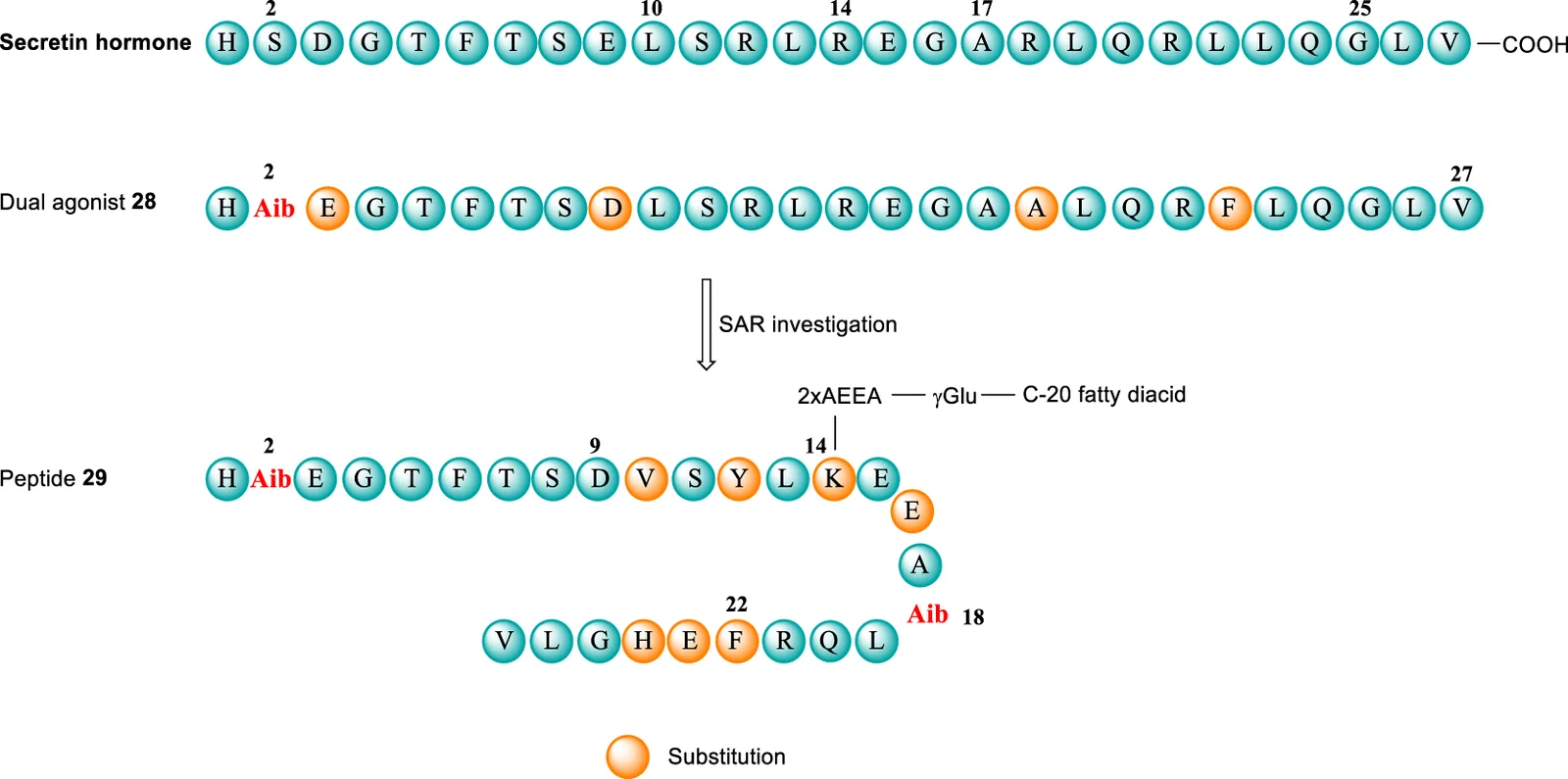
Amino acidic sequence of secretin hormone, dual GLP-1R/SCTR agonist **28**, and optimization studies toward peptide **29**.

#### 3.1.4 Triple agonism toward GLP-1R/GIPR/GCGR

The simultaneous activation of GLP-1R, GIPR, and GCGR with triple agonists owns the potential to enhance the therapeutic efficacy by synergistic regulation of both energy intake and expenditure (Li et al., 2024). Retatrudide (Figure 11) is a 39 amino acid single peptide engineered by Ely Lilly from GIP backbone to have triple GLP-1R/GIPR/GCGR agonism (Coskun et al., 2022). The peptide includes three non-coded amino acid residues, namely, Aib2, for stability against DPP-4 cleavage; α-methyl-L-leucine at position 13, contributing to an optimal glucagon and GIP activity; and Aib20, beneficial for GIP activity, good PK profile, and chemical feasibility. The backbone is conjugated to a C-20 fatty diacid moiety through a linker at Lys17, providing albumin binding as strategy to extend the half-life. When evaluated *in vitro*, retatrutide was 2.9-fold less potent than human glucagon toward hGCGR (EC_50_ values of 5.79 nM vs. 1.97 nM, respectively), 8.9-fold more potent at hGIPR than GIP(1–42)NH_2_ (EC_50_ values of 0.0643 nM vs. 0.574 nM, respectively), and 2.5-fold less potent than GLP-1 (7–36)NH_2_ vs. hGLP-1R (EC_50_ values of 0.775 nM vs. 0.312 nM, respectively) in the corresponding receptor activation assays. In hepatocytes, the peptide exhibited similar potency to native glucagon in stimulating glucose output; while, in adipocytes, it was more potent than the native GIP in promoting lipolysis. Similar to semaglutide, preliminary assessment of GIPR and GLP-1R activity through the intraperitoneal glucose tolerance test showed that the compound exerted improved glucose tolerance toward all evaluated genotypes (i.e., the wild type, GIPR-null mice, and GLP-1R-null mice). Upon administration on C57/Bl6 diet-induced obese mice, retatrutide determined a dose-dependent reduction in body weight, primarily due to reduction in fat mass with minimal impact on lean mass, and improved metabolic health. It provided decreased blood glucose and plasma insulin, suggesting improved glycemic control with beneficial effect in insulin sensitivity. Moreover, the drug improved hepatic function as demonstrated by decrease in plasma alanine aminotransferase, and liver triglycerides. Furthermore, retatrutide was able to increase energy expenditure and weight loss through GCGR engagement in obese mice, as evidenced by reduced efficacy when co-treated with GCGR antibody antagonist. Evidences from first-in-human phase 1 clinical study in a single ascending dose showed that the compound was well tolerated, and determined appetite regulation, with significant weight loss effects after 4 weeks of once-weekly dosing (clinicalTrials.gov, 2019). The overall results of safety, tolerability, and efficacy profile supported the current advancement of retatrutide in phase 3 clinical trial (clinicalTrials.gov, 2026m; ClinicalTrials.gov, 2026l; ClinicalTrials.gov, 2026k). Preliminary results from a 80-week, randomized, double-blind, placebo-controlled master trial, comparing retatrutide’s safety and efficacy vs. placebo in adults with overweight and obesity, showed that the drug was able to reduce body weight by 19.0%, 25.9%, and 28.3% for 4 mg, 9 mg, and 12 mg doses, respectively, corresponding to an absolute weight loss of approximatively 21–32 Kg.

Several studies demonstrated that co-administration of GLP-1R and GIPR agonists outperforms monotherapy promoting atherogenic lipoprotein clearance, and attenuating atherosclerotic lesion development (Modder et al., 2021). On the contrary, for triple agonists, cardiometabolic benefits still remain unexplored. In this context (Zhou et al., 2026; Han et al., 2021), Zhou and collaborators disclosed the first GLP-1R/GIPR/GCGR triple agonist with high efficacy in both obesity and atherosclerosis treatment (Zhou et al., 2025). The novel series of peptides was designed starting from the peptide template **30** (Yang Y. et al., 2022) (Figure 23b; EC_50_ = 3.39 nM (GLP-1R), 6.61 nM (GIPR), 107.46 nM (GCGR) in CRE-Luc production functional assay). Through a solid-phase 4-component Ugi macrocyclization (exemplified in Figure 23a), a protractor moiety including a mini-PEG spacer, γ-glutamic acid, and a C-20 fatty diacid was efficiently inserted into the glutamate-lysine mutated exocyclic bridge, spanning i−i+4 residue pairs in the region Tyr10 to Ala16. Many noncanonical amino acids were introduced at specific positions to enhance the proteolytic stability, including Aib, 1-aminocyclobutane-1-carboxylic acid, 2-fluoro-L-Phe, α-methyl-L-Leu, and N^6^-acetyl-L-lys.

**FIGURE 23:**
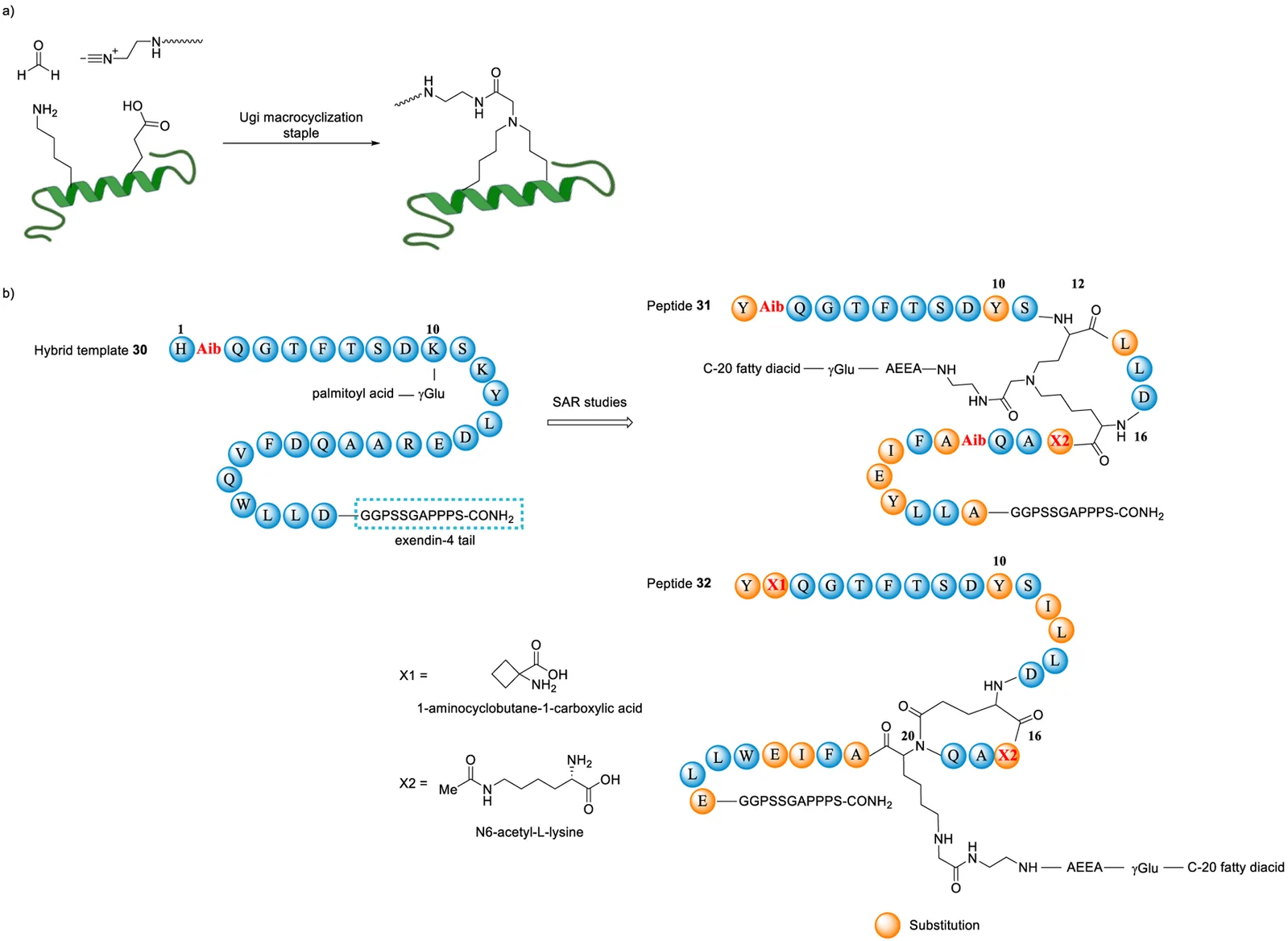
**(a)** Schematic representation of Ugi-cyclization; **(b)** Rational design toward the synthesis of **31–32** peptides, starting from template **30**.

Most peptides showed (sub)nanomolar potency toward one or two receptors in CRE-Luc production functional assay. Peptide **31** (cyclized at residues 12–16) and **32** (cyclized at residues 16–20) displayed a % of endogenous ligand potency greater than 50% across all three receptors. Moreover, while peptide **31** showed a biased triple agonism with a dominant GCGR activity, peptide **32** exhibited a more selective profile. In detail, the latter was more potent toward the hGLP-1R (with EC_50_ value of 126.3 pM, vs*.* hGLP-1 (7–37), EC_50_ value of 139.4 pM); while appeared less potent toward GIPR (with EC_50_ value of 29.2 pM, vs. hGIP EC_50_ value of 24.7 pM) and GCGR (with EC_50_ value of 250.2 pM vs. hGCG EC_50_ value of 199.5 pM). Studies of circular dichroism proved that peptide **32** maintained a more stable and increased helical structure (compared to semaglutide) possibly attributed to the conformational constraint conferred by the lactam bridge. Moreover, the peptide showed improved stability in rat serum, probably due to enhanced proteolytic resistance given by the staple constraint. PK studies in Sprague-Dawley rat showed prolonged half-life (13 h vs. ~8 h for semaglutide). In further *in vivo* studies, after a single dosing in lean mice, the compound showed a dose-dependent GLP-1R agonism based on glucose tolerance increase, acute food intake inhibition, transient reduction in body weight, and improved potency (compared to the reference semaglutide). In preclinical models of obesity, peptide **32** prevented diabetes progression with higher efficacy than the clinically approved GLP-1R/GIPR dual agonist tirzepatide. Finally, peptide **32** enhanced hepatic lipid clearance and fibrosis amelioration (compared to tirzepatide), partly attributed to glucagon-mediated fibroblast growth factor 21 induction, alleviating also the endothelial-to-mesenchymal transition, a key process implicated in atherosclerosis progression, thereby demonstrating its therapeutic potential as lead candidate, with balanced activities toward targeted receptors. Moreover, it showed improved PK parameters supporting once-weekly administration in humans, demonstrating the critical role of triple agonists beyond their application solely aimed at the regulation of glucose levels, representing a valuable tool to treat atherosclerosis and related diseases.

More GLP-1R/GIPR/GCGR triple agonists were developed by Zheng and co-workers and culminated with the discovery of the clinical candidate **33** (Figure 24), to potentially treat obesity and related metabolic diseases, including NASH (Zheng et al., 2026). The new derivatives were synthesized according to a “2-in-1” PEG-fatty acid stapling platform (Pflimlin et al., 2019). Three representative i−i + 7 cysteine pairs at positions 14–21, 17–24, and 21–28 were selected as candidate mutation sites for the introduction of the staple, because were solvent-exposed and seemed to not perturb receptor-binding interfaces. Peptide **33** (with a staple 21–28) emerged for its potent and balanced agonism (EC_50_ values of 33.9 pM (GLP-1R), 12.5 pM (GCGR) and 92.9 pM (GIPR) in receptors activation assays), resulting 3-fold more potent than the reference retatrutide toward GLP-1R activation (EC_50_ value of 101.2 pM). In rodents and non-human primates, the new peptide displayed extended plasma exposure and extended half-lives [rat (21.5 h, intravenous; 26.4 h, subcutaneous), cynomolgus monkey (83.3 h, intravenous; 97.2 h, subcutaneous), and minipig (101.9 h, intravenous; 129.0 h, subcutaneous)], together with good bioavailability (50.9, 75.7, and 122.4, respectively). The lead was then evaluated in two NASH models, characterized alternatively by a rapid and pronounced fibrotic responses, or by the progressive metabolic and inflammatory features of the disease. Indeed, peptide **33** demonstrated consistent therapeutic efficacy across both models enabling reduction in steatosis, inflammation, and fibrosis, as well as downregulation of fibrogenic markers, thus supporting its clinical development as next-generation of unimolecular multitargeting incretin-based therapeutic for metabolic and liver diseases.

**FIGURE 24:**
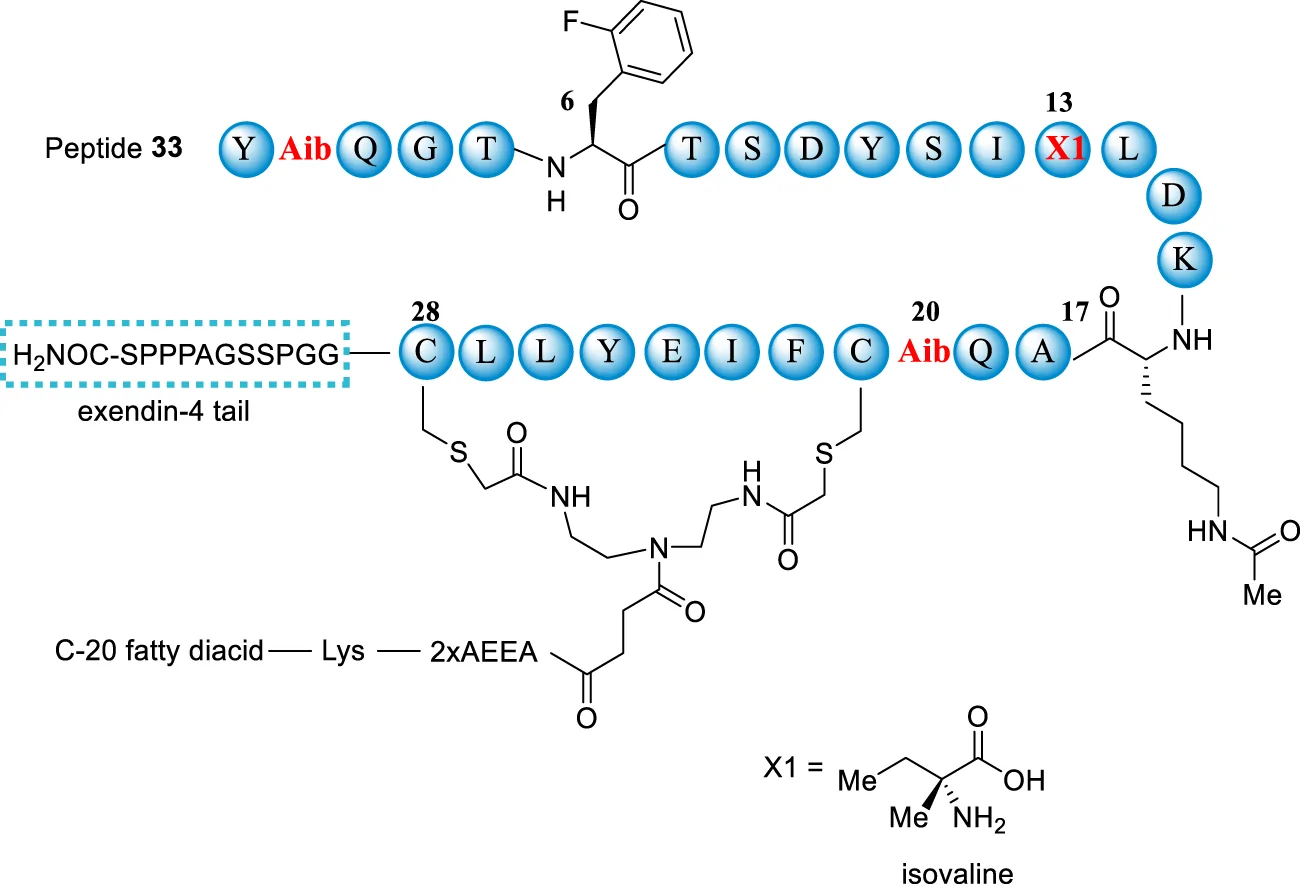
Amino acidic sequence of peptide **33**.

New triple agonists were also developed by Liu and collaborators (Liu X. et al., 2025) starting from the hypothesis that concurrent activation of GLP-1R, GIPR, and NPY2R could efficiently promote insulin secretion, body weight reduction, and lipid metabolism. The structure of two previously discovered potent GLP-1R/GIPR dual agonists (peptides **34**–**35**, Figure 25) (Jing, 2023) was used as template to design this new series by (i) connecting either peptide **34** or **35** to PYY-2 analogue (see Figure 15), and introducing a cysteine residue at the C-terminus of **34** or **35**, and a 3-maleimidopropionic acid at the OEG junction of the N-terminus of the PYY-2 analogue; or (ii) by directly combining peptides **34** or **35** with the PYY-2 segment. Amongst the newly synthesized conjugates, derivatives with peptide **35** as GLP-1/GIP part and PYY-2 as the NPY2R agonistic segment effectively retained the potency toward all three receptors with compound **36** showing the best EC_50_ values of 0.0036 nM for GLP-1R, 0.17 nM for GIPR, and 75.2 nM for NPY2R in receptors activation assays.

**FIGURE 25:**
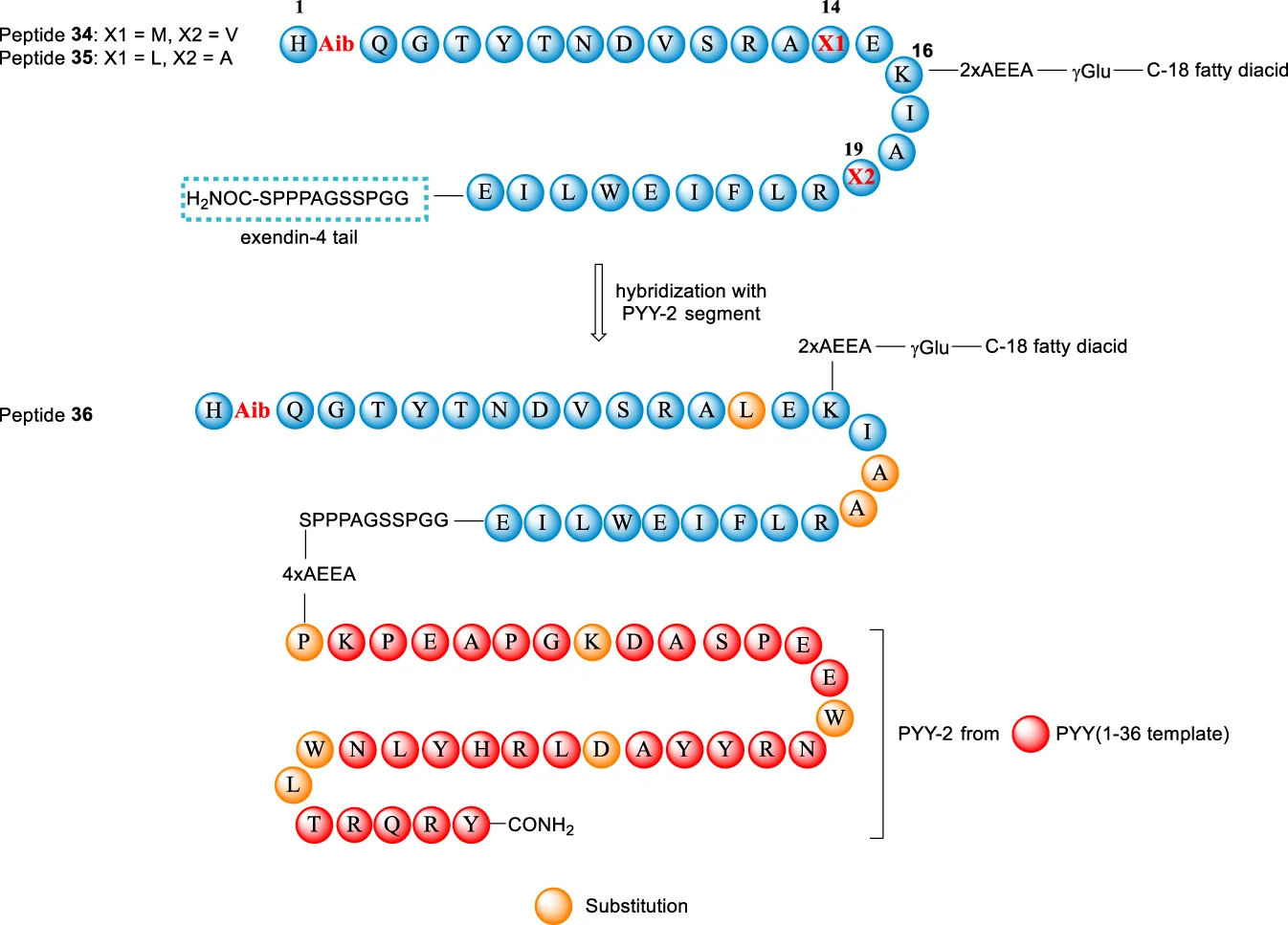
Rational design toward the discovery of peptide **36** derived from hybridization between precursors **34–35** and PYY-2.

Moreover, peptide **36** exerted high anorexigenic effect, and, in the long-term hypoglycemic experiment in diet-induced obesity mice, it quickly reduced blood glucose with a more stable and sustained effect as the administration time prolonged. Finally, treatment with **36** positively impacted on lipid metabolism and weight control, favoring liver function improvement, supporting the advantage of integrating gut hormone synergies beyond the canonical glucagon axis. Peptide **36** emerged as the first in-class GLP-1R/GIPR/NPY2R tri-agonist.

#### 3.1.5 Tetra-receptor agonists

The first-in-class chimeric tetra-agonists for GLP-1R/GIPR/GCGR/NPY2R were developed combining four different hormonal functions that potentially synergize to boost the therapeutic effect, and reduce the side effects (Dinsmore et al., 2025). While the cognate native ligands of GLP-1R, GIPR, GCGR share a sequence homology, PYY(1–36) belongs to a structurally divergent class and, for this reason, the introduction of NPY2R agonism represented a main challenge. Preliminary exploration focused on the tri-agonist **37** (Figure 26), (Finan et al., 2015) and introduction of a C-terminal exendin-4 tail was envisioned to afford a structural spacer between the N-terminal segment responsible for GLP-1R, GIPR, GCGR activation, and the NPY2R-targeting domain. Next, introduction of truncated PYY(1–36) (i.e., PYY(24–36)) at position 33 of the C-terminus produced the first modified derivative **38** (Figure 26). In cAMP-dependent CRE-Luc production functional assay, this compound exhibited 6.7-fold decrease NPY2R agonism (EC_50_ value of 400 pM) compared to the native PYY(1–36), with strong GLP-1R activation (EC_50_ value of 0.4 pM, 6.5-fold potency increase over native GLP-1), but with significant drop in potency toward both GCGR (EC_50_ value of 120 pM) and GIPR (EC_50_ value of 280 pM). Retatrutide was evaluated as an alternative template containing Aib2 and Aib20, which confer receptor agonism and resistance toward DPP-4 hydrolysis. Truncation of retatrutide (for the structure see Figure 11) at the diacid lipid moiety of both positions 17 and Ser32, and introduction of PYY(1–36) at the position 33 of the C-terminal afforded peptide **39** with better functional profile (6.5-fold increase in GLP-1R potency, EC_50_ value of 0.4 pM, vs. GIPR EC_50_ value of 2.3 pM; 11-fold reduction in GCGR activation (EC_50_ value of 130 pM); and a 2.8-fold decrease in NPY2R potency (EC_50_ value of 170 pM)), highlighting the critical role of the exendin-4 tail for optimal receptor engagement of these multi-agonists (Coskun et al., 2018; Knerr et al., 2022).

**FIGURE 26:**
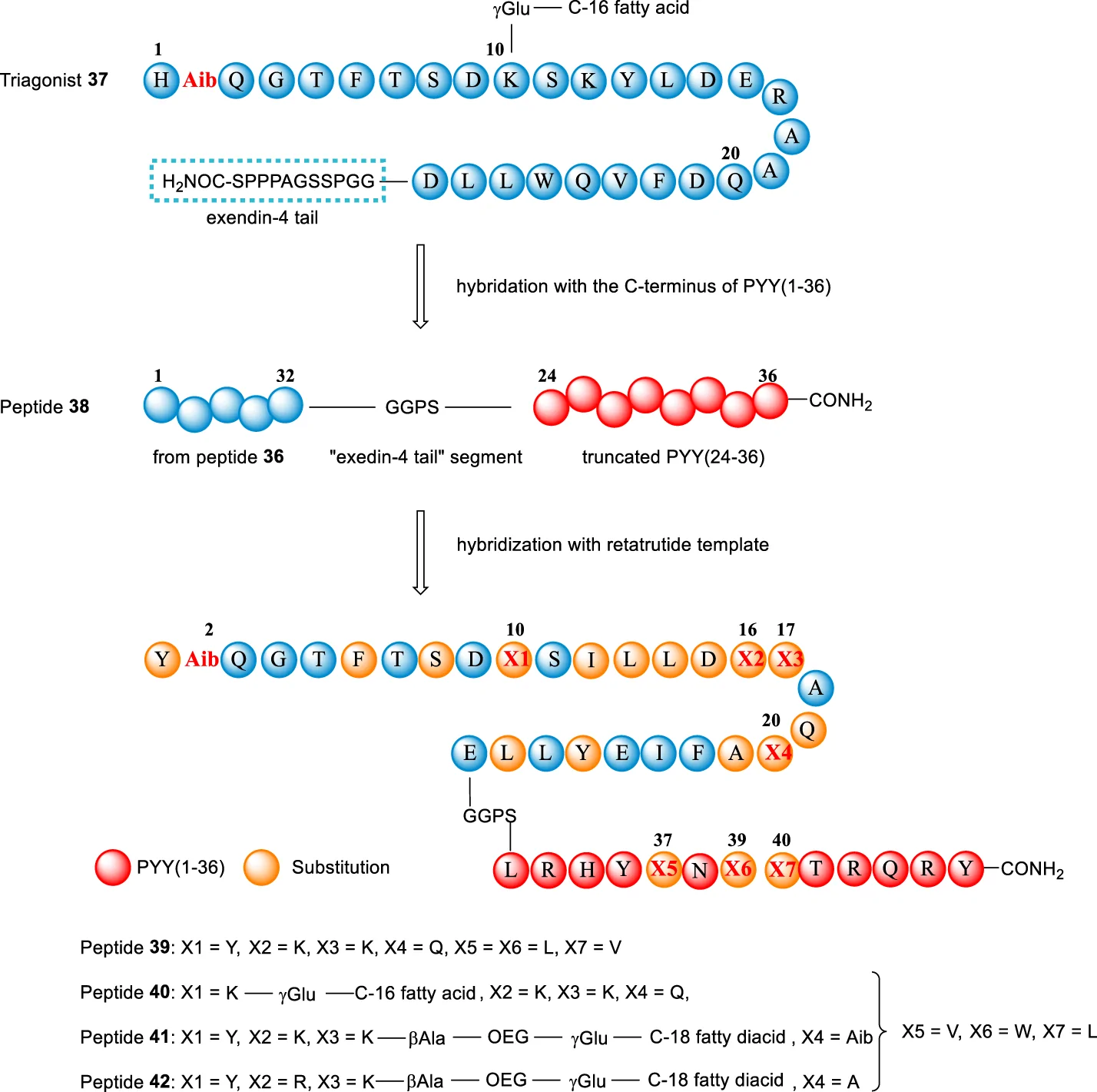
Structure optimization studies of peptides **37–38** toward novel tetra-agonists **39–42**.

A second derivative **40**, featuring lipidation at position 10 with a γ-*L*-glutamic acid and a modified PYY C-terminus (L37V, L39W, and V40L) showed equipotent activity toward GLP-1R (EC_50_ value of 2.1 pM), a 3.6-fold increase at GIPR (EC_50_ value of 0.47 pM), a 2.5-fold decrease at GCGR (EC_50_ value of 30 pM), and similar NPY2R potency (EC_50_ value of 55 pM). Further exploration of the lipid/protractor moiety focused on lipid diacids, known to impart considerable albumin affinity and prolonged *in vivo* circulation compared to monoacid lipids. Introduction of Aib residue at position 20 to reinforce α-helical structure, and variation of the linker composition (by inserting βAla-OEG-γE-C-18 fatty diacid motif) yielded **41**, which showed a modest improvement in GLP-1R potency, 4.7-fold enhancement vs. GIPR, 1.9-fold decrease vs. GCGR, and 7-fold reduction toward NPY2R (EC_50_ value of 1.1 pM (GLP-1R), 0.75 pM (GIPR), 23 pM (GCGR), and 420 pM (NPY2R)). Two further substitutions on peptide **41** (Lys16Arg and Aib20Ala) scaffold gave peptide **42**, which displayed uniform decrease of potency toward GLP-1R, GCGR, and NPY2R, while maintaining robust GIPR agonism (EC_50_ value of 31 pM (GLP-1R), 0.98 pM (GIPR), 81 pM (GCGR), and 1,100 pM (NPY2R) making it an interesting compound able to mitigate the nausea associated to GLP-1R and NPY2R activation. All synthesized constructs were evaluated for their ability to induce biased agonism, and peptide **40** was the only peptide chimera showing a distinctly biased profile, with significant reduction in β-arrestin-2 recruitment (43% of GLP-1), while maintaining potent cAMP signalling. When the effect of hSA on the potencies of the tetra-agonists was evaluated, only peptide **41** showed a relevant EC_50_ shift (EC_50_ values of 0.17 pM (for 0% hSA), 23 pM (with 2% hSA)), probably due to its diacid lipidic tail. Finally, peptides **40–42** showed resistance to proteolysis with GLP-1R EC_50_ values of 21 pM (40), 2.0 pM (41), and 41 pM (42) in absence of DPP-4 enzyme, and 27 pM (40), 4.6 pM (41), and 75 pM (42) after incubation with the protease. These evidences reinforce the design of unimolecular, tetra-chimeric agonists with expanded and tunable pharmacology, offering the potential to be a viable alternative therapy for patients abandoning the treatment because of nausea and vomiting.

#### 3.1.6 GLP-1 conjugates

GLP-1 conjugates aim to address the intrinsic limitations of native peptide therapeutics, including rapid enzymatic degradation, and short systemic half-life. Indeed, the covalent attachment of chemical moieties, such as, fatty acids, polymers, or other functional groups, helps to improve the metabolic stability, to modulate the receptor pharmacology, and finally promote a prolongment of the systemic exposure, by enhancing reversible plasma protein binding or reducing renal clearance.

##### 3.1.6.1 Antibody GLP-1 conjugates

The use of antibodies has been rapidly growing because of their significant efficacies toward complex multifactorial diseases (Elshiaty et al., 2021). Early this year, Wu and co-workers disclosed novel antibody conjugates combining GIPR antagonism with GLP-1R agonism with a potential use in obesity treatment (Wu et al., 2026). A series of hybrid molecules were prepared using synthetic GLP-1 analogues (e.g., [Aib8,22; Gly36]GLP-1 (7–37) and cyclic [Glu22-Lys26; Aib8; Gly36]GLP-1 (7–37)) tethered to a IgG-based GIPR through a suitable linker, enabling independent tuning of the antibody, of the linker, and of the peptide components (exemplified by the structure of Figure 27). The conjugation was carried out via C-terminal bromoacetamide on the linker, which selectively alkylated the engineered cysteine thiol yielding a stable thioether linkage. Three conjugation sites (i.e., D88C on the light chain near the N-terminal region, T487C on the Fc near the C-terminal region, and E384C on the Fc close to the hinge region) were evaluated for the conjugation of the antibody, to investigate their impact on the stability, activity, and PK profile of the resulting products. While D88C site resulted less favorable for linking the peptides, all constructs conjugated at the E384C and T487C sites exhibited comparable potency, with EC_50_ values ranging from 9.7 to 33.6 nM toward GIPR inhibition quantified through cAMP functional production. *In vivo* studies showed that E284C conjugation had the most beneficial effect on PK profile, with half-lives around 5.3 days. Indeed, in db/db mice, E384C conjugates generated greater and more durable body weight reduction. To enable efficacy studies in obese nonhuman primates, four GLP-1 analogues, [Aib8,22;G36], [Aib8; E22;G36], [Aib8,22;E15;G36], and [Aib8; Y16; E22;G36] GLP-1 (7–37) were conjugated to hGIPR-Ab E384C. Among these, the derivative hGIPR-Ab E384C [Aib8;Tyr16;Glu22;Gly36] (G4S)3Lys) retained robust activity in both the hGLP-1R agonism and hGIPR antagonism (EC_50_ value of 48.7 pM (GLP-1), IC_50_ value of 21 nM (GIPR)) and was selected for further *in vivo* studies. In a single-dose tolerability assay in obese cynomolgus monkeys, the conjugate was highly stable over the study period. Preliminary results from a phase 2 double blind, randomized, placebo-controlled clinical trial, once-monthly subcutaneous dosing of the conjugate (140 mg, 280 mg, or 420 mg every 4 weeks without dose escalation; 420 mg every 8 weeks without dose escalation; 420 mg every 4 weeks with 4-week dose escalation; 420 mg every 4 weeks with 12-week dose escalation; or placebo) demonstrated a substantial weight reduction in patients with obesity with or without T2D, with the mean % change in body weight from baseline to week 52 ranging from 12.3% to 16.2%, vs. 2.5% with placebo). In the obesity-diabetes cohort, the mean % change in body weight reduction from baseline to week 52 ranged from 8.4% to 12.3% (vs. 1.7% with placebo). Moreover, the glycated hemoglobin level decreased of 1.2%–1.6% in the treated groups, compared to 0.1% of the placebo group. Finally, the gastrointestinal adverse events were common, but occurred less frequently with gradual dose escalation and lower starting dose.

**FIGURE 27:**
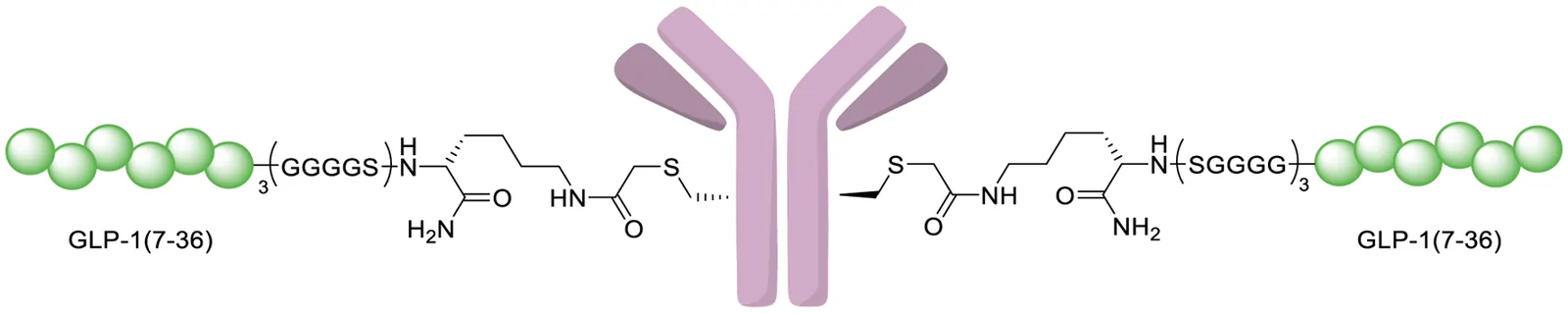
General representation of antibody GLP-1 via engineered cysteine handles.

These outcomes prompted its advancement in to phase 3 clinical trials under the brand name MariTide (Véniant et al., 2024; Jastreboff et al., 2025).

##### 3.1.6.2 Dual-fatty acid GLP-1 conjugates

Exogenous insulin still remains a reliable treatment for T2D, but its use is linked to the insurgence of drawbacks, like weight gain, and hypoglycemia, requiring daily frequent injections. GLP-1R agonists enable potent glucose-lowering effects, weight loss benefits, and cardiovascular protection, with a significant lower risk of hypoglycemia, but the dose-dependent induction of nausea and vomiting hamper their therapeutic application. Fc fusion, PEGylation, and fatty acid modifications have been explored as strategies to obtain long acting GLP-1R agonists with improved PK profile. In this context, last year, Wang and co-workers disclosed new GLP-1 conjugates modified with dual fatty acid connected by two γGlu linkers, and having at the C-terminus a hydrophilic polypeptide consisting of 100 amino acids, and including repetitive proline, alanine, and serine (PAS100) residues in a defined order, to improve the aqueous solubility (affected by the dual lipidation) (Wang C. et al., 2025). In the first set of GLP-1 analogues, a spacer consisting of 1–5 OEG units was introduced between the peptide and two C-12 fatty acid linked at the α- and ε-amino groups, respectively, of Lys20 (2 × γGlu-Lys module) (Lau et al., 2015). Further modifications were focused on the lipid portion of the conjugate, given its central role in determining the potency of the drug (as for liraglutide). In a second sets of GLP-1 analogues, the α-amino group of the lysine was coupled to either C-12 or C-14 fatty acid chains, while diacids ranging from C-12 to C-18 were introduced at the ε-amino position (Figure 28). Biological results, determined through GLP-1R activation assay measured by time-resolved fluorescence resonance energy transfer, showed that the gained high lipophilicity of the derivatives had a key role in the receptor activation potency, with peptides **43–44** being the most promising compounds (EC_50_ values of 128.3, 60.35 pM, respectively; relative efficacy of 122.66, 123.38%, respectively). Although the introduction of a double fatty acid substitution impacted on the hydrophobicity of the conjugates, the presence of PAS100 significantly increased the overall solubility (3.24 and 2.79 mg/mL, for **43** and **44**, respectively). *In vivo*, peptide **43** emerged for its balanced profile, and, in particular, it induced glucose-lowering, and weight-reducing effects similar to what observed for semaglutide. Surface plasmon resonance showed stronger receptor affinity of peptide **43** with association rate constant (k_a_) 13 times higher than semaglutide (1.35 × 10^4^ 1/M⋅s vs. 1.02 × 10^3^ 1/M⋅s), indicating faster receptor engagement. Overall, peptide **43** emerged as potent long-acting GLP-1R agonist that combines improved solubility, receptor affinity, and potent *in vivo* glucose-lowering effects, highlighting the potential of the dual-fatty acid conjugation combined with PASylation as versatile platform to be extended to other lipidated peptide therapeutics.

**FIGURE 28:**
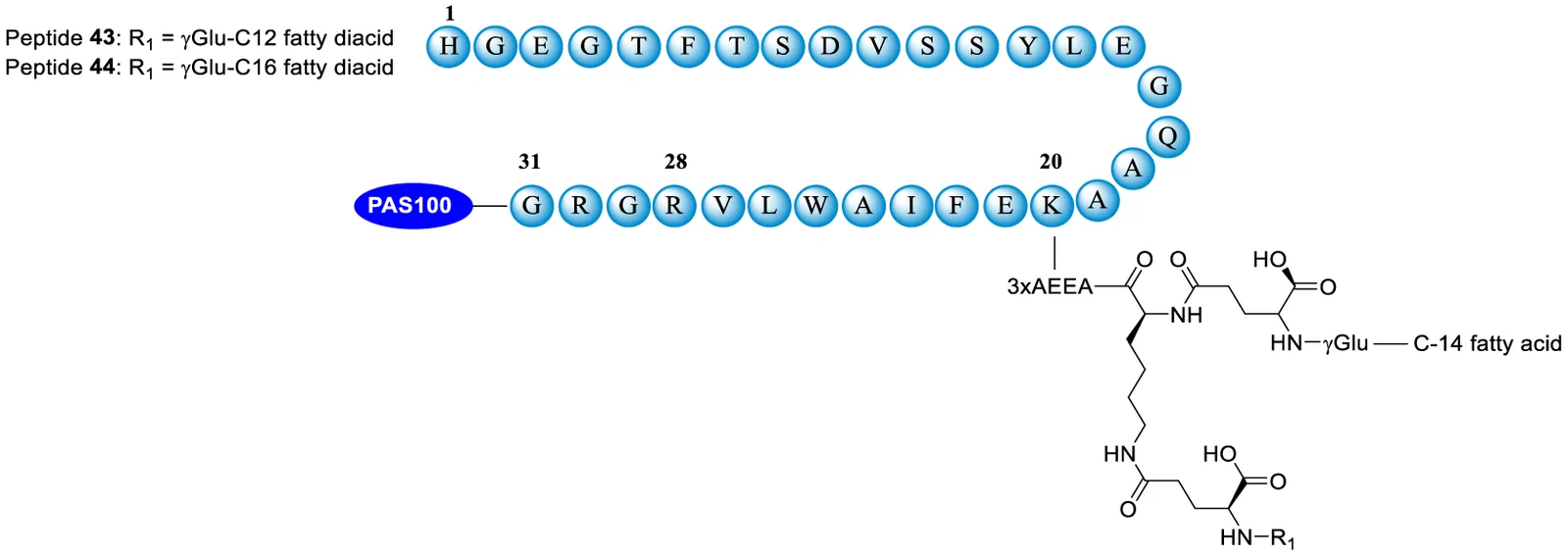
General structure of dual-fatty acid GLP-1 conjugates **43**–**44**.

Conjugation with small molecule protractors represents the most successful strategy to extend peptides half-life, likely due to its low cost, and low immune risk. To date, long-chain fatty acids are the only class of small molecule protracting moieties currently validated in clinical. Semaglutide (including a C-18 diacid) and tirzepatide and insulin icodec both conjugated to a C-20 diacid chain, are notable examples. As previously mentioned, when a fatty acid chain is inserted at the side amino group of a lysine residue, GLP-1 can bind to albumin, and reach a very long residence time in blood circulation. However, the low solubility of long chain fatty diacids and the moderate binding affinity to hSA (K_D_ = 0.6–2.0 μM) limit their use.

Recently, Jing and co-workers developed new conjugates, evaluating the potential of coomassie brilliant blue as an alternative to the fatty C-18 diacid chain for GLP-1 protraction to potentiate its efficacy in T2D therapy (Jing et al., 2025). This dye is widely used to detect proteins on polyacrylamide gels, but it showed a potential in clinical use for retinal surgery, and to treat spinal injuries. The zwitterionic structure makes coomassie brilliant blue a highly water-soluble protractor suitable for oral administration. Moreover, it owns strong binding affinity to albumin and can engage proteins via non-covalent van der Waals forces, and heteropolar interactions. Starting from the structure of semaglutide, new conjugates were designed by preserving the peptide backbone, while selectively replacing the C-18 diacid protractor with the coomassie brilliant blue at Lys20. A series of linkers varying in length (spanning 6 to 34 atoms), and including alkyl chains, phenyl groups, carboxylic acids, sulfonic acids, and PEG units were synthesized. Further SAR studies focused on the optimization of the GLP-1 peptide sequence. A small set of three GLP-1 analogues with natural amino acids were truncated at the C-terminal and derivatized at Lys20 or Lys28 or at both positions, with the coomassie brilliant blue protractors, while Gly2 (inspired from dulaglutide and albiglutide) or Ser2 replaced the non-coded Aib2. Peptide **45** (Figure 29a) exhibited the highest potency with EC_50_ value below 0.5 pM in CRE-Luc production functional assay. In BALB/c mice, after subcutaneous injection, compound **45** showed a half-life much longer than semaglutide (t_1/2_ = 32 h vs. 18 h), and delayed T_max_ (6 h vs. 12 h), suggesting a slow and measured release from the subcutaneous depot. In diabetic mice, compound **45** displayed a dose-dependent antihyperglycemic effect (EC_50_ value of 30 nmol/kg vs. 2 nmol/kg for semaglutide, in a dose-response study calculated on the basis of AUC blood glucose during 0–96 h after dosing) with a duration of action (blood glucose <10 mM) reaching up to 48 h; it also reduced food intake, suggesting its potential of gastric emptying and obesity management. Finally, peptide **45** was able to reversibly bind collagen and hSA, with K_D_ values of around 0.5 μM demonstrating that the specific affinity to albumin is not mandatory for peptide protraction, and pan-binders might be useful.

**FIGURE 29:**
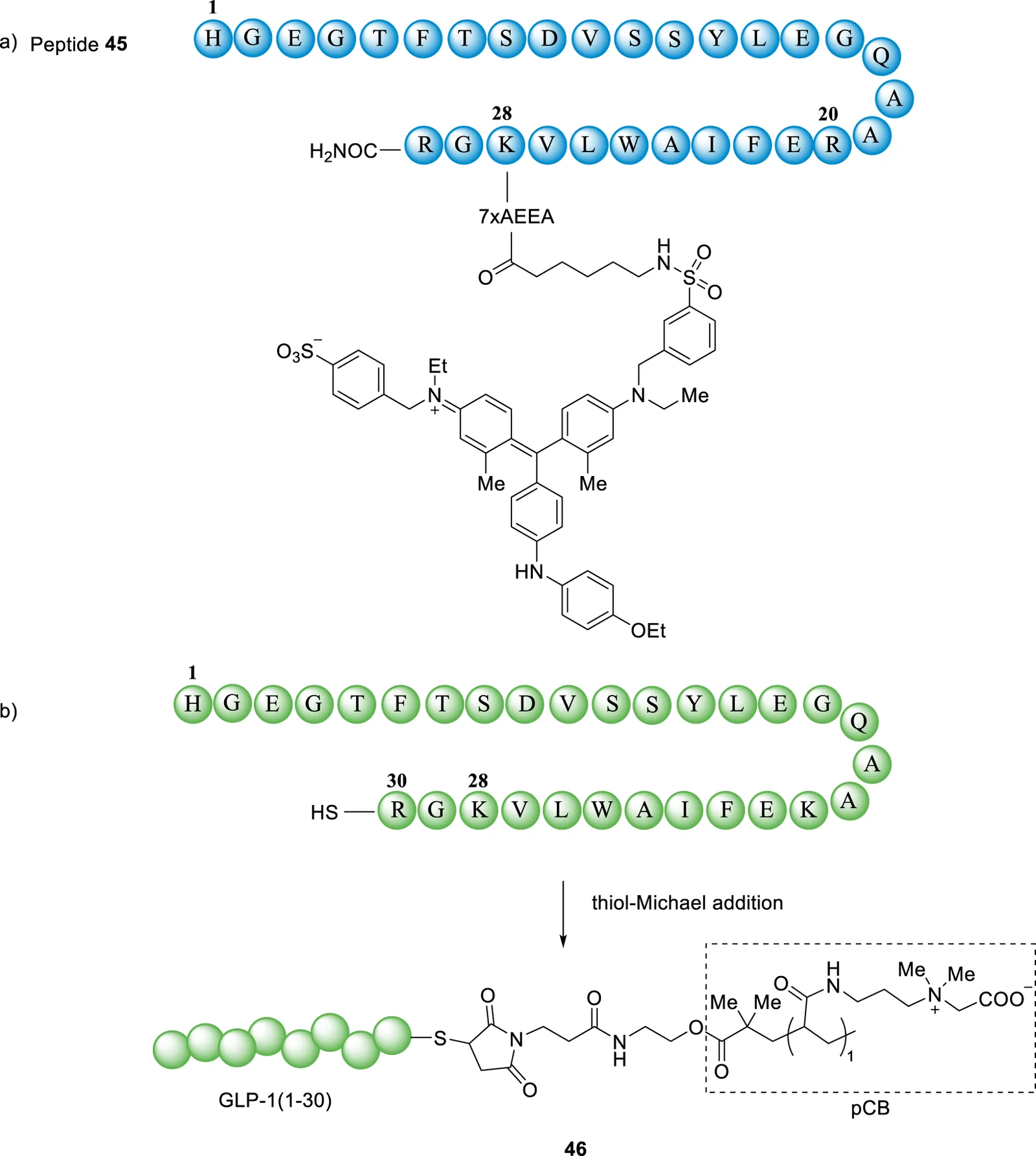
**(a)** General structure of the conjugate peptide **45**; **(b)** Chemical structure of the zwitterionic conjugate **46** derived from truncated (1–30) GLP-1–SH.

##### 3.1.6.3 Zwitterionic polymer conjugates

Fast proteolytic inactivation and renal clearance limit the clinical use of native GLP-1. Along with PEG, Fc integration, lipidation, and conjugation with polysaccharides (Ichikawa et al., 2018) zwitterionic polymers have also been exploited to ameliorate GLP-1 PK properties, prolonging its half-life *in vivo* up to >30 h (Teng et al., 2021). Tsao and co-workers reported the zwitterionic GLP-1 polymer **46** (Figure 29b) as a proof-of-concept that conjugation of the super-hydrophilic poly (carboxybetaine) (pCB) may provide a sustained and long-term glycemic control in T2D management (Tsao et al., 2020; Han Y. et al., 2018). The polymer was conjugated through a thiol-maleimide click chemistry to the C-terminus of GLP-1 modified with a cysteine residue, to ensure a site-specific conjugation (Selis et al., 2012). To avoid renal secretion, a 66 kDa pCB polymer was selected, and the new bioconjugate exhibited an α-helix structure similar to GLP-1. *In vitro,* on rat pancreatic/islet RIN-m5F cells, the derivative showed EC_50_ value of 7.24 nM (vs. 1.08 nM of GLP-1 calculated through *in vitro* insulin secretion assay) which was in line with previous evidences (Mu et al., 2013; Bukrinski et al., 2017). In male C57BL/6 mice, polymer **46** exhibited long circulation half-life with maximum serum concentration observed (C_max_ of 1,427.1 ng/mL at T_max_ = 13.4 h). In additional *in vivo* studies, **46** protected GLP-1 from proteolytic cleavages in the bloodstream, with prolonged glycemic control up to 6 days, supporting further use of poly (carboxybetaine) conjugation as valuable strategy to prolong glycemic control.

##### 3.1.6.4 GLP-1 conjugate with lysosome-targeting chimeras

Lysosome-targeting chimeras represent a novel technology designed to induce the degradation of specific disease-associated proteins through receptor-mediated endocytosis and lysosomal delivery (Banik et al., 2020). Molecular targets are mainly proteins located on the cell surface or outside the cell, which have been undruggable through conventional methods (Li et al., 2026). Conversely, proteolysis-targeting chimeras work degrading intracellular proteins through the ubiquitin-proteasome system, limiting the action to cytosolic or nuclear proteins with accessible intracellular domains. A couple of years ago, Zhu and co-workers disclosed a novel lysosomal targeting receptor ligand GLP-1-based, as new avenue for the concurrent treatment of multiple diseases, such as T2D and several cancers (Zhu et al., 2023). Previous findings reported the remarkable success of GLP-1 conjugates in efficient delivery of antisense oligonucleotides and estrogens to pancreatic β cells (Knerr et al., 2021). Two different modifications of GLP-1 were evaluated as peptide carrier, i.e., the azide-tagged GLP-1^N3^ (exemplified in Figure 30) and the biotin-tagged GLP-1^Biotin^, and labeled with cyanine 5-NHS for easy monitoring to study the internalization process. The peptides were self-assembled with a dibenzocyclooctyne-tagged binder of the protein of interest (e.g., extracellular proteins (GFP and Neutravidin) and cell membrane proteins (i.e., epidermal growth factor receptor and programmed death-ligand 1)). Cetuximab, a FDA-approved epidermal growth factor receptor antibody drug, and the programmed death-ligand 1 monoclonal antibody drug-Sudubrilimab were selected as targeted binders. Upon conjugation via click chemistry, the resulting heterochimera could recruit both GLP-1R and the protein of interest. The formation of this ternary complex could trigger receptor-mediated internalization, followed by lysosomal trafficking of the protein of interest, ultimately leading to its degradation within lysosomes. To probe the effective GLP-1R-mediated endocytosis, cervical cancer line HeLa or fibroblast cell line L-929 were incubated with the labeled peptides for 24 h, confirming intracellular accumulation. Next, to demonstrate the potential of lysosome targeting chimera GLP-1R-driven, a study with the non-covalent avidin (K_d_ <10^−15^ M) was used in HeLa cells, in the presence or absence of GLP-1^Biotin^ for 4 h or 24 h. The results showed that co-incubation with GLP-1^Biotin^ for 24 h led to a 4-fold increase in cellular fluorescence (compared to cells treated with either avidin alone or together with biotin only). Avidin was internalized into cells with addition of GLP-1^Biotin^ and delivered into lysosome with excellent R value (0.84), demonstrating the crucial role of GLP-1 in regulating the uptake, which resulted time-dependent, reaching the top concentration at 6 h. Next, the ability of the conjugate to degrade membrane proteins was evaluated, and, to this purpose, the epidermal growth factor receptor tyrosine kinase and the programmed death-ligand 1 were selected. GLP-1 conjugation enabled the cellular uptake and degradation of these membrane proteins on several cell lines, in a concentration-dependent manner, beginning after 4 h of treatment with the addition of GLP-1^N3^, and progressing to almost full erasure at 48 h, thus confirming this peptide carrier attractive for the design of new hetero-chimera, enabling efficient lysosomal degradation of both extracellular and membrane proteins.

**FIGURE 30:**
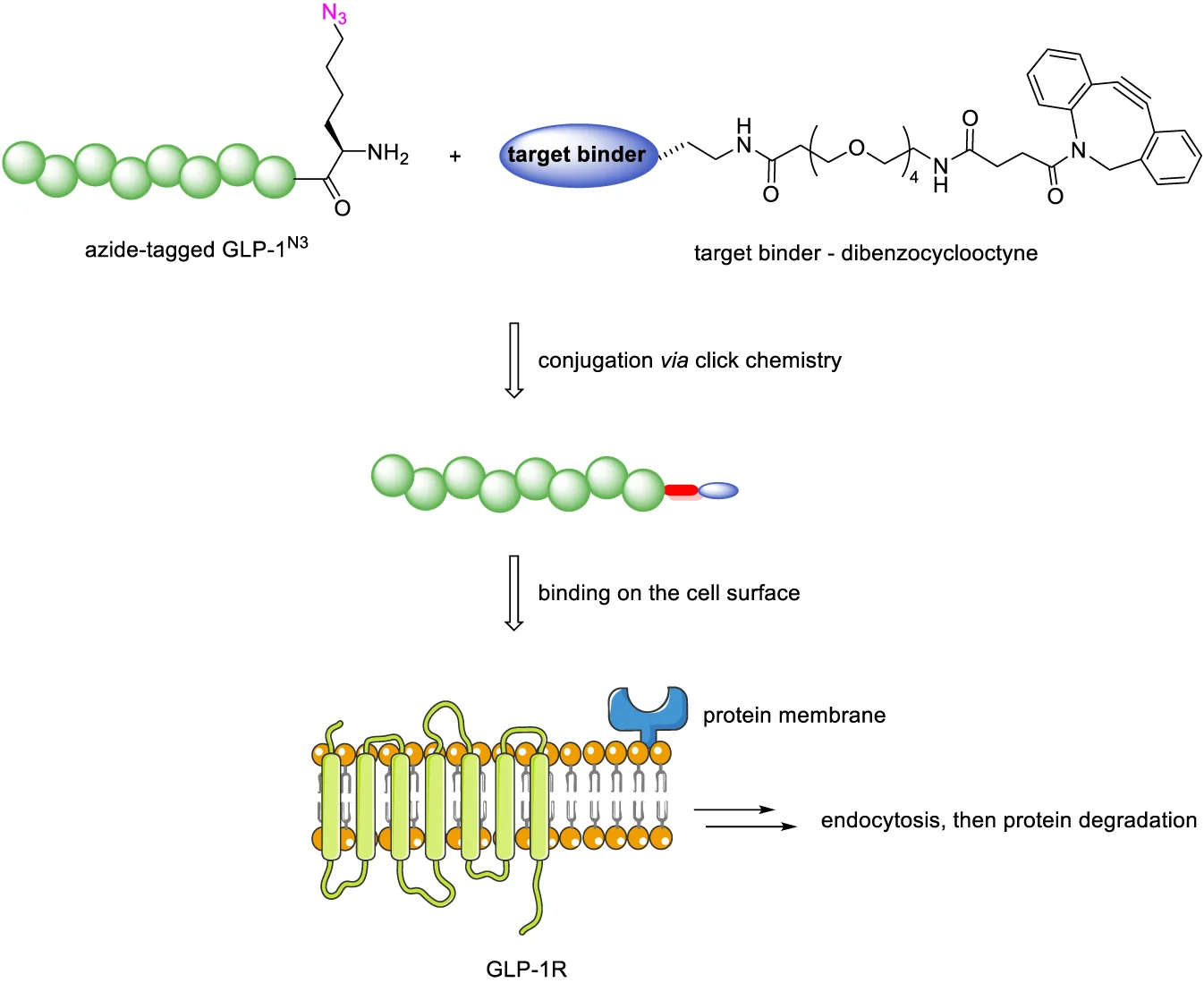
General scheme of lysosome-targeting chimeras GLP-1-based.

#### 3.1.7 GLP-1-based delivery systems

Conventional formulations of semaglutide (e.g., oral tablets) suffer from low bioavailability (∼1%), and strictly require daily dosing. Moreover, the low lipophilicity of the peptide contributes to its reduced cellular uptake. Many oral delivery systems were explored to overcome these issues (Sandmeier et al., 2025; Kweon et al., 2023), and among all, nanotechnology-based drug delivery is emerging to enhance drug stability, prolong the circulation time, and facilitate targeted delivery to metabolic tissues (Engin et al., 2024). In 2025, Lee and co-workers disclosed new semaglutide-based nanoparticles incorporating a rosuvastatin–lipid conjugate, to treat obesity and related metabolic disorders (Lee et al., 2025). The novel statin–lipid conjugate was developed combining rosuvastatin (Figure 31a) with ursodeoxycholic acid (i.e., liver-protective agent) to promote drug encapsulation and self-assembly, and providing complementary therapeutic benefit for clinical translation. Indeed, previous works demonstrated that this conjugate not only preserved the cholesterol-lowering function of statins, but also inhibited the reabsorption of bile acids, thereby promoting cholesterol consumption (Lee et al., 2024). The new nanoparticles were self-assembled through hydrophobic interactions and van der Waals forces between the fatty acid moieties of the statin conjugate and semaglutide (Figure 31b). The formulation was evaluated in high-fat diet-fed mouse model, demonstrating ~30% loss in body weight together with better glucose regulation. Moreover, it showed ~47% improvement in liver function markers compared to the control group, suggesting a protective role against liver damage caused by excessive fat accumulation. This could be exploited in preventing non-alcoholic fatty liver disease and related metabolic complications.

**FIGURE 31:**
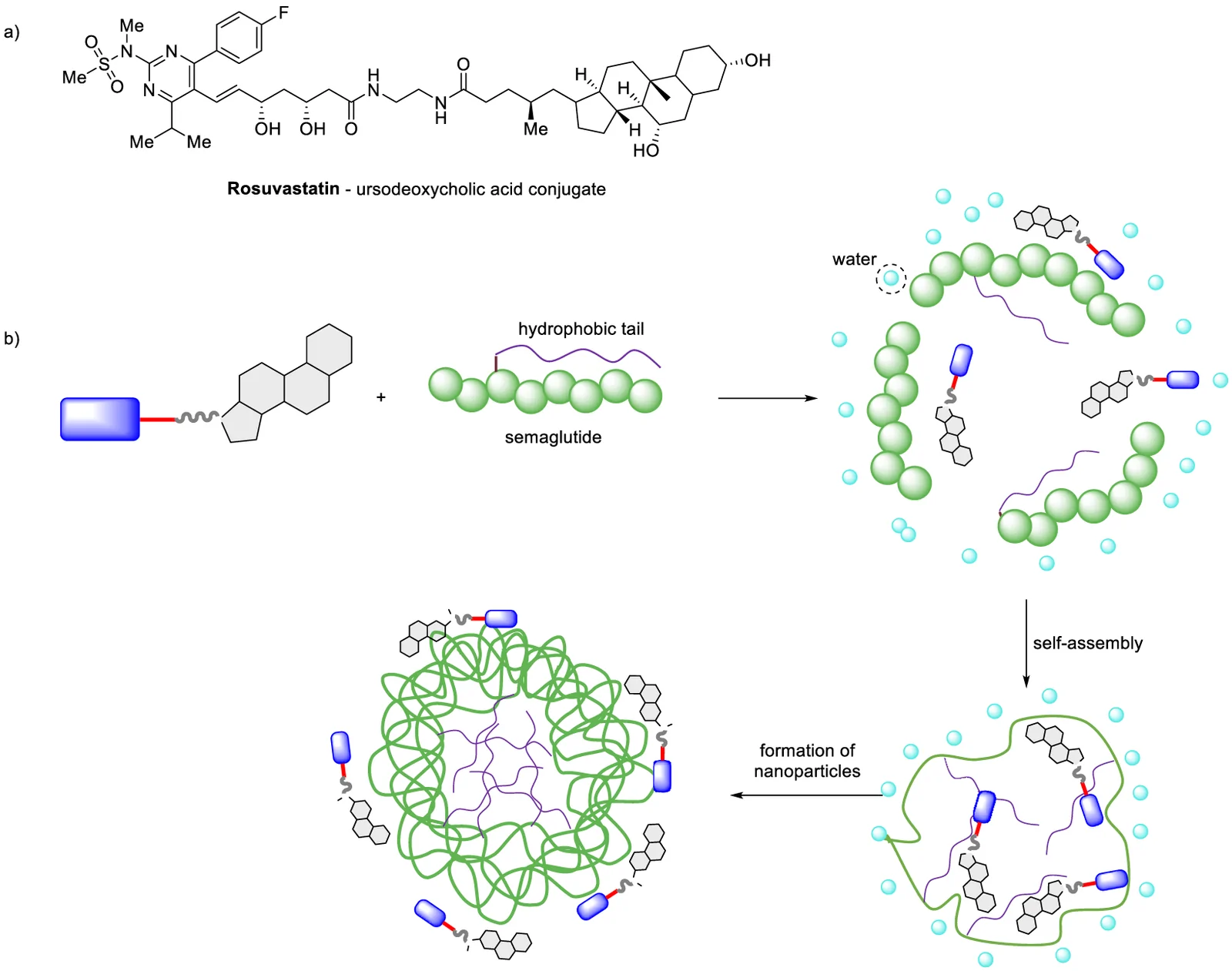
**(a)** Chemical structure of rovustatine–lipid conjugate; **(b)** Schematic representation of self-assembled statin–lipid conjugate nanoparticles in aqueous media.

Marques and collaborators reported the preliminary development of new glucose-sensitive pH-responsive nanoparticles loaded alternatively with exenatide or semaglutide as potential formulation to treat type-1 diabetes (Marques et al., 2024). The idea was to generate an original pancreas biomimetic consisting of α and β cells (differentiated from human induced pluripotent stem cells and immobilized on a biofunctional matrix), which incorporated glucose-responsive nanoparticles encapsulating a GLP-1R peptide agonist. The engineered pH-sensitive nanoparticles were designed to release the GLP-1 analogue in response to decreased environmental pH. The payload (semaglutide or exenatide) was selected to stimulate glucose responsiveness of differentiated β cells, being still immature and not fully responsive. The microspheres were then transplanted into diabetic mice, and the glycemic level was monitored for 1 month. Analysis of blood glucose concentration confirmed a reduction in hyperglycemia compared to the control group. However, the study raised few issues in both *in vitro* and *in vivo* settings, starting from the high mortality rate observed in transplanted animals, likely due to the β cells destruction in the absence of treatment. Additional studies are required to deeply evaluate the impact of this cell therapy in diabetic mouse model. Nevertheless, the study posed the basis for further exploration of stem cell-based nanotherapeutics, to cure type-1 diabetes and related disorders.

### 3.2 Novel recently developed peptide agonists of other GPCRs

#### 3.2.1 AMYR agonists

As already mentioned, pramlintide (Figure 3) was the first synthetic short-acting amylin analogue to receive FDA approval for hyperglycemia’s management. However, the peptide drug suffers from short half-life and requires multiple daily injections. Main degradation products derive from deamidation and isomerization of asparagine residues at positions 3, 21, and 22 (and with a lesser degree at positions 14 and 35). Strategies to extend the duration of action, including peptide acylation (protraction), were adopted to overcome such drawback (Gydesen et al., 2016), and due to these challenges, amylin analogues require acidic pH formulation.

Petrelintide (co-developed by Zealand Pharma and Roche, Figure 4) was designed as chemically stable, long-acting analogue of h-amylin, which allowed coformulation at neutral pH with other peptides used for weight management therapies (Fischer Munch et al., 2025). The drug was obtained after a deep SAR investigation on native amylin, suggesting that (i) N-methylation of Gly24 and Ile26 (the corresponding unnatural N-methylglycine, N-methylisoleucine at positions 22 and 26, respectively, in petrelintide) reduced fibrillation (even at high micromolar concentrations); (ii) C-terminal replacement of Tyr37 with proline (characteristic of salmon calcitonin) or hydroxyproline residues was beneficial; (iii) deletion of Asn21 and Asn22 improved the chemical stability, as these two residues easily undergo deamination and hydrolysis; (iv) the peptides solubility improved when Asn3, Asn14 and Asn31 were replaced with glycine, (*S*)-aminohexanedioic acid and glutamic acid, respectively, thus modifying the overall isoelectric point toward the acidic region; (v) the potential of forming a salt bridge in the central region of the backbone helped to stabilize the α-helical structure and confer high stability; (vi) replacement of the labile disulfide bridge with a more stable bond at the N-terminus (e.g., amide bond, or thioether (Patch et al., 2022), cystathionine and lactam bridges (Fischer et al., 2019)) was useful to avoid dimerization.

In Sprague−Dawley rats, the drug candidate showed favorable PK profile, with T_max_ around 24 h, C_max_ about 2-fold higher compared to other analogues, and half-life of 33.8 h, (suitable for once-weekly dosing in humans) (Kurtzhals et al., 2023). When evaluated for chronic weight management in diet-induced obese rats, petrelintide displayed a dose-dependent effect on food intake and body weight. No fibrillation or significant covalent oligomerization was detected in formulations based on common tonicity agents at neutral pH (e.g., sodium chloride, mannitol, and propylene glycol) at concentrations ranging from 1 to 10 mg/mL; and the peptide showed good chemical stability. Petrelintide progressed to phase 2 clinical trials and is currently under investigation for T2D treatment and chronic weight management in adults with obesity or overweight (ClinicalTrials.gov, 2026a; ClinicalTrials.gov, 2026f). Very recent preliminary results from single and multiple ascending dose randomized, placebo-controlled, and double-blind trials revealed that the drug was well tolerated, with no serious or severe treatment-emergent adverse events. Moreover, it showed a half-life of approximatively 10 days, and body weight reduction up 10.7% at week 42 (vs. 1.7% with placebo; p-value<0.001).

Cagrilintide (Figure 4) was developed by Novo Nordisk as long-acting, lipidated amylin analogue with enhanced metabolic stability, and prolonged PK properties compared to pramlintide (Kruse et al., 2021), given the higher beneficial effect of conjugation with fatty diacids compared to monoacids (liraglutide vs. semaglutide) observed for GLP-1 derivatives. Chemical stability was optimized by targeting deamidation of asparagine residues, to enable formulation at neutral pH. One of the main challenges was indeed the inherent propensity of h-amylin to form amyloid. This tendency was expected to enhance by insertion of lipophilic fatty acid moieties, due to an increase of intermolecular attractive forces (Bech et al., 2018). To limit this, a h-amylin based template was chosen for SAR investigation, and Pro25, Pro28 and Pro29 residues were retained to reduce β-sheet propensity. Furthermore, introduction of salt-bridges in the central region of the amylin sequence and insertion of solubilizing mutations were evaluated. Mutation with lysine residues, and subsequent acylation at positions 1 and 3 of the N-terminal, at residues 11 and 18 of the helical part, and at amino acids 21, 28, and 31 of the C-terminal did not affect the biological activity. Amidation of the C-terminal site was essential for bioactivity with very little room for modification. The disulfide bridge near the N-terminus was also required, while Lys1 could be removed without significant loss of activity. The amino terminus seemed optimal for fatty diacids conjugation. Cagrilintide (with a C-20 fatty diacid at the K1, and a γGlu linker) was selected as the best candidate (EC_50_ values of 49 pM (hAMYR_3_), 62 pM (hCTR) on CRE-Luc production functional assay; 348 pM (rAMYR_3_), 287 pM (rCTR) on cAMP assay; receptor binding IC_50_ values of 170 pM (hAMYR_3_), 223 pM (hCTR); solubility of 200 μM (in the pH range 4.0–8.0)), and showed good resistance toward fibril formation. The compound included (i) 14E and 17R amino acids which were envisioned to stabilize the central helix via a salt bridge; (ii) 25P/28P/29P residues derived from r-amylin to reduce β-sheet propensity and fibril formation; (iii) a C-terminal proline to enhance the potency toward CTR; (iv) a N-terminally linked C-20 fatty acid to give longer *in vivo* exposure. In rats, cagrilintide determined a sustained decrease of around 80% in food intake for several days at doses in the range of 1–10 nmol/kg. Prolonged duration of action was observed with the sustained suppression of appetite in the food intake screening model, and was next confirmed in PK studies in Sprague Dawley rats (t_1/2_ = 20 h and Cl (L/h/kg) = 0.00377 (intravenous). The lag-time in fibril formation propensity assay at pH 7.5 was of 41 h, with 76% of recovery. Cagrilintide advanced in clinical trial, and is currently undergoing in Phase 3 alone (clinicalTrials.gov, 2026g; clinicalTrials.gov, 2025i; clinicalTrials.gov, 2026q), or in coadministration with semaglutide (named CagriSema) for weight management in adults with T2D (clinicalTrials.gov, 2025j; clinicalTrials.gov, 2026p; clinicalTrials.gov, 2026c; Frias et al., 2023). Results from a phase 3a, 68-week, multicenter, double-blind, placebo- and active-controlled trial showed that co-administration of semaglutide and cagrilintide (both at 2.4 mg dose) determined significant and clinically relevant body weight reduction in adults with overweight or obesity (20%–30% or more) (Garvey et al., 2025; Aroda et al., 2026; Buse et al., 2026; Rosenstock et al., 2026; Hamarsheh et al., 2026). The gastrointestinal side effects like nausea, vomiting, diarrhea, constipation, or abdominal pain were transient and with mild to moderate severity, affecting 79.6% of patients in the cagrilintide-semaglutide group, and 39.9% in the placebo group. In another phase 3a, double-blind, randomized, placebo-controlled trial conducted in 12 countries, once-weekly cagrilintide-semaglutide (at a dose of 2.4 mg each) produced greater weight loss compared to placebo (13.7% vs. 3.4%), improved glycemic control, with 73.5% vs. 15.9% of patients achieving glycated hemoglobin less than 6.5%, although gastrointestinal adverse events were more frequent, transient, and predominantly mild to moderate. In a group of people with early stage T2D, cagrilintide-semaglutide combination confirmed higher efficacy compared to placebo in reducing glycated hemoglobin (1.8% and 1.5% at 40 weeks at 2.4 mg and 1.0 mg doses, respectively, compared to 0.1% reduction with placebo).

The same research group disclosed peptide **47** (Figure 32), a novel long-acting h-amylin analogue with a potential in weight management in the clinical setting (Dahl et al., 2024). Inspired by pramlintide, the authors were interested on identifying the minimal and optimal number of mutations of the h-amylin scaffold to achieve selectivity toward AMYR over CTR, with the concomitant improvement of plasma half-life and solubility, while reducing the tendency to form fibrils. Starting from the h-amylin sequence, the new peptides retained the disulfide bridge Cys2−Cys7, and were acylated with an albumin binder on the ε-amino group of Lys1, in the attempt to reduce renal clearance. In accordance to previous findings (Abedini et al., 2016), two proline residues were introduced in the peptide backbone at positions 21 and 27. Moreover, to reduce fibril formation propensity, a neutral formulation was envisaged by inserting negatively charged (e.g., Asp14) residues able to give an acidic isoelectric point. Peptide **47** (acylated at the side chain amino group of Lys1 with a C-20 fatty diacid-γGlu- γGlu), emerged for the highest h-AMYR selectivity (EC_50_ values of 177 p.m. (hAMY_3_R), 5,210 pM (hCTR), measured by CRE-Luc production functional assay; 262 (rAMY_3_R), 102,500 (rCTR), measured by cAMP receptor assay; hCTR/hAMY_3_R = 29), and resulted resistant to physical stress for more than 40 h. Following subcutaneous injection in rats, the peptide was able to reduce appetite by approximately 50% or more after the first 24 h, with sustained effect from 24 to 48 h. Moreover, in a study of sub chronic efficacy in diet-induced obese rats fed with a high energy diet, the lead showed reduced food intake with a maximal effect occurring during the first 2 days (4.7% at the 0.7 nmol/kg dose; 8.7–9.3% at 2.2, 6.6, 22 nmol/kg doses), and slowly diminished with time. The effect of the drug was primarily driven by the loss of fat mass with minimal impact on lean mass.

**FIGURE 32:**
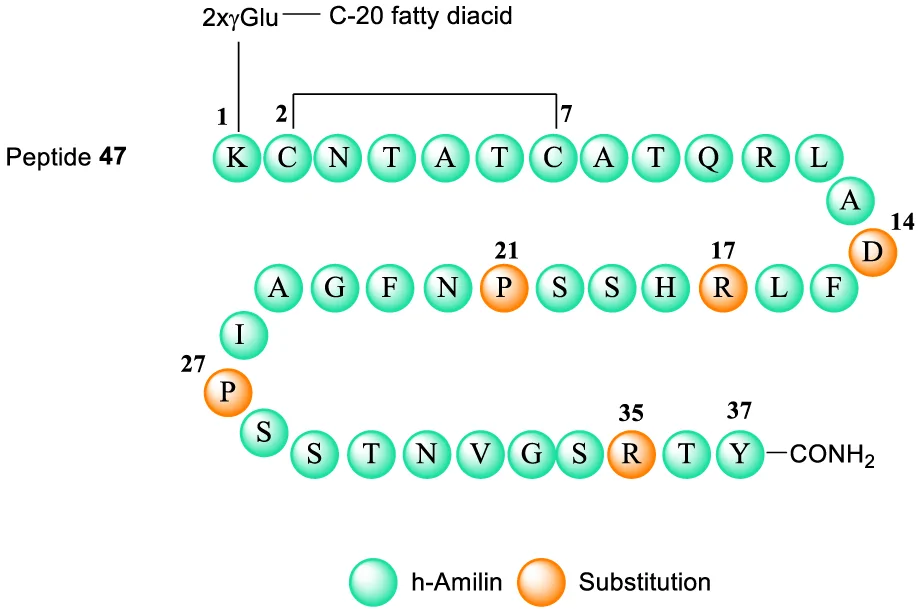
Amino acid sequence of AMYR agonist **47**.

#### 3.2.2 Dual AMYR and CTR agonists (DACRAs)

As previously discussed, despite the remarkable success of GLP-1-based therapy, several drawbacks limit the use of agonists in obesity management. Many patients only achieve modest weight loss or quickly reach a plateau, often showing severe gastrointestinal side effects. In addition, loss of lean muscle mass significantly impairs physical function, and therapy discontinuation often causes rapid weight regains. On the other hand, both amylin and calcitonin have long been considered challenging drug targets, due to their pronounced amyloidogenic susceptibility. The presence of N-terminal disulfide bonds makes these peptides highly prone to reduction, accelerating self-assembly and fibril formation. This aspect has significantly hindered their development in stable formulations. Notably, a well-established correlation exists between fibril formation and the onset of various amyloid-related pathologies (Marmentini et al., 2022; Koopman et al., 2017). In this regard, helix-stabilizing stapling approaches have proven effective in ameliorate formulation stability ensuring therapeutic safety (Nielipińska et al., 2024; Ghareeb and Metanis, 2023). The reinforcement of α-helical conformation (e.g., by triazole bridging via click reaction, side-chain lactam formation, and formaldehyde-mediated cyclization between lysine and tyrosine, namely, KaY stapling) has been widely exploited to avoid fibrillation, and improve the *in vitro* stability and formulation properties of amylin- and calcitonin-based therapeutics (Babych et al., 2024; Ghareeb and Metanis, 2023; Tinsley et al., 2021). Moreover, combination therapy with GLP-1R agonists and DACRAs can improve patient outcomes in obesity treatment. DACRAs are a class of therapeutic peptides that simultaneously activate both AMYR and CTR with beneficial effects beyond those of amylin alone, and thus emerged as promising next-generation anti-obesity drugs. Numerous studies supported the therapeutic efficacy of DACRAs in eliciting a unique combination of weight loss, antinociception and bone protection, in obese patients with osteoarthritis (Katri et al., 2019).

Last year, Zheng and co-workers disclosed peptide **48** (Figure 33), an ultralong-acting and non-aggregating DACRA (Zheng et al., 2025a), identified by eliminating the disulfide bond of the native calcitonin template, and by restoring the structural integrity with the insertion of a PEG-fatty acid stapling between 8 and 15 amino acids. Formulation stability assessment at two concentrations (1.0 and 10.0 mg/mL), alone and in combination with semaglutide, showed high stability with the opportunity to develop a fixed-dose combination. PK studies in multiple species revealed low systemic clearance (3.5 mL/h/kg in rats, 0.2 mL/h/kg in minipigs, and 0.2 mL/h/kg in cynomolgus monkeys), ultralong elimination half-life (t_1/2_ = 20 h in rats, 150 h in both minipigs and cynomolgus monkeys), and high subcutaneous bioavailability (F = 66.1% in rats, 54.5% in cynomolgus monkeys). Moreover, peptide **48** exhibited a robust pharmacological efficacy inducing sustained weight loss, with selective adipose reduction rather than lean mass loss. The use of this drug attenuated the rapid weight rebound typically observed after the discontinuation of GLP-1R agonist-based therapies, supporting its potential to deliver more durable results in long-term obesity treatment. Compound **48** is currently under investigational new drug-studies and might advance soon in clinical phase.

**FIGURE 33:**
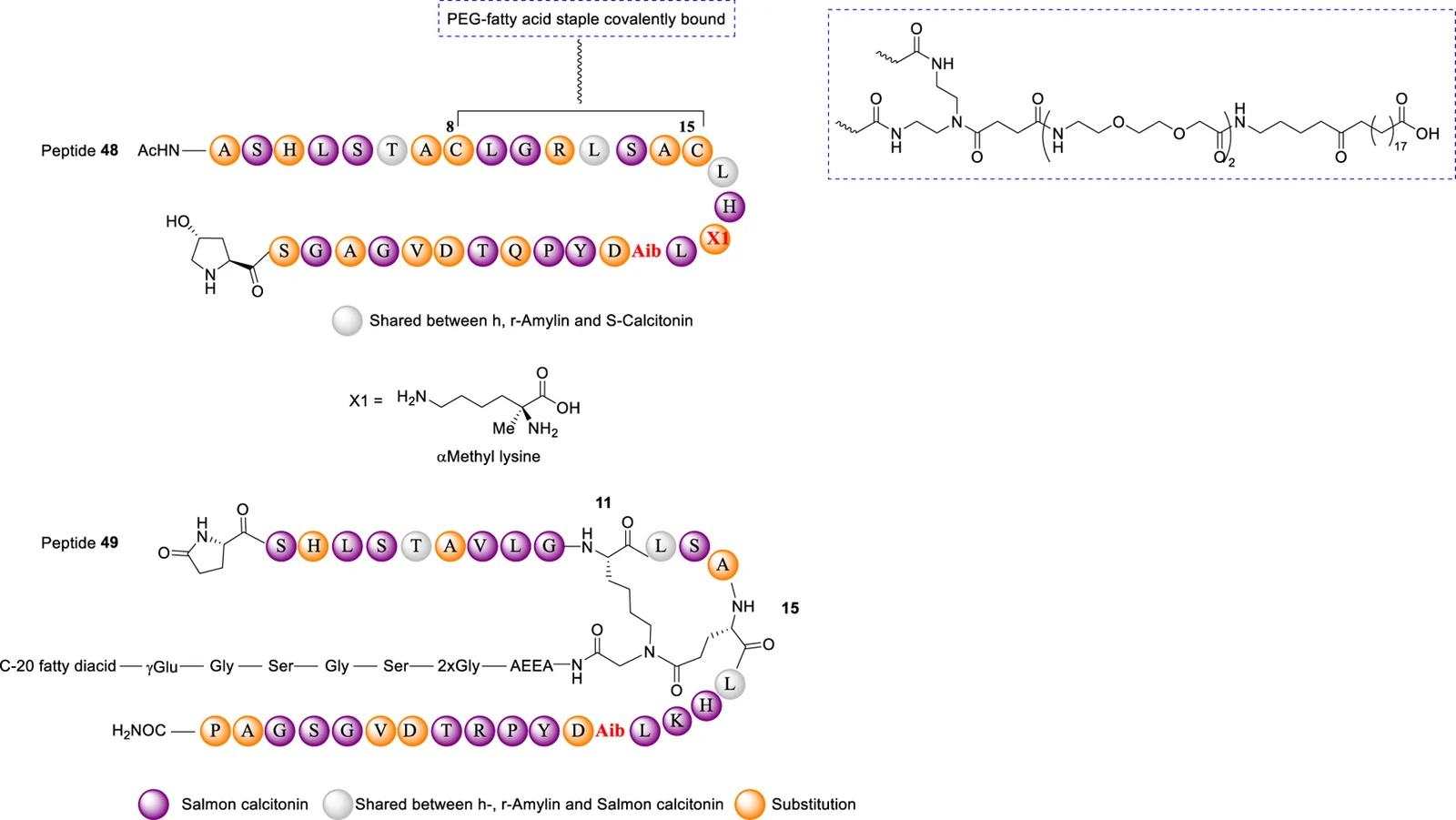
Amino acid sequence of the novel DACRA **48–49**.

Another example of long-acting stapled DACRA obtained from the native calcitonin template was reported early this year (Zhou et al., 2026). In this case, after removal of the C1–C7 disulfide bridge, known to be highly susceptible to enzymatic reduction, the N-terminal was reinforced through the introduction of a Ugi-stapling macrocycle to obtain a new template (see peptide **49**, Figure 33). SAR exploration of the helix stapling impact on bioactivity alteration was performed by a systematic Ugi-staple screening through a [Lys-Glu]-mutated lactam bridge spanning the i−i+4 residue distance. Moreover, the insertion of a pyroglutamate group to shield the free N-terminal amine together with an intrachain α-methylalanine substitution were evaluated to reinforce the helicity. Circular dichroism studies confirmed that almost all stapled peptides possessed an increased content of helix, in comparison to the linear parent compound. *In vitro* biological activity of the newly obtained peptides was assessed by measuring the accumulation of cAMP upon receptor activation in hHEK-293 stably expressing both the hAMY_3_R and CTR, and salmon calcitonin was used as positive control for both receptors. Compound **49** emerged as more promising with a balanced dual receptor engagement activity (EC_50_ (95% of confidence interval) pM of 0.8 and 0.6, against AMY_3_R and CTR, respectively). It also showed promising PK properties in Sprague-Dawley rats after single intravenous injection (1.0 mg/kg), and subcutaneous (3.0 mg/kg) administration. More in detail, it had a long half-life (t_1/2_) of 15 h, and slow clearance rate of 7.0 mL/h/kg when administrated intravenously; while, bioavailability of 65.9% was detected after subcutaneous administration. The increased proteolytic stability conferred by the constraint, together with the prolonged circulation time due to the albumin interaction mediated by the lipophilic Gly-Ser-Gly-Ser-Gly-Gly side-chain modification, supported the potential use of the peptide for once-weekly dosing in humans. In diet-induced obesity models, the staple peptide **49** strongly reduced body weight and ameliorated liver function, with synergistic benefits when co-administrated with semaglutide or tirzepatide, making it a promising candidate for multi-hormone treatment of obesity and related metabolic disorders, and highlighting the crucial role of Ugi macrocyclization as versatile platform for helical peptides.

#### 3.2.3 Novel peptide ligands targeting oxytocin receptor

Oxytocin (Figure 34) is a nine-residue cyclic neuropeptide produced in the hypothalamus and secreted by the posterior pituitary gland (Jurek and Neumann, 2018). It binds the oxytocin receptor (belonging to Class A GPCR family), which is expressed in the periphery and central nervous system. In addition to the control of parturition uterine contractions, social bonding, and nursing milk letdown, recent evidences demonstrated the role of oxytocin in promoting glucose uptake and lipid utilization in adipose tissue and skeletal muscle (Hong et al., 2021). For this reason, it emerged as promising pharmacological tool to guide the development of tailored anti-obesity therapeutics. To overcome the short half-life of the neuropeptide (∼2–3 min), many semi-synthetic analogues (e.g., carbetocin, [Ser(4),Ile (8)]-oxytocin, [aminosuberic acid (1,6)]-oxytocin) were developed, and seemed to ameliorate the metabolic parameters in obese and diabetic mouse models (Zhang et al., 2013). Pflimlin and collaborators reported the design and synthesis of a novel series of oxytocin derivatives (exemplified by derivative **50**, Figure 34), as long-acting, peripherally restricted oxytocin receptor agonists with potential therapeutic benefits in obesity treatment (Pflimlin et al., 2020). The new derivatives were designed by conjugating the oxytocin peptide backbone with a multi-PEG linker and fatty acid moieties via a cysteine thioether to increase the stability and *in vivo* half-life. Three linkers were evaluated, including (i) a short PEG spacer bound to myristic acid (C14 chain, FA1); (ii) a longer PEG spacer conjugated to the octadecane dioic acid (C-18 chain, FA2); (iii) two separate PEG spacers combined with a lysine and the octadecane dioic acid (C-18 chain, FA3). Compound **50** showed the most potent *in vitro* activity and full agonism toward oxytocin receptor, with EC_50_ value of 2.58 nM in β-arrestin assay. Moreover, it showed enhanced interaction with hSA, improved stability, and long *in vivo* half-life. Given the well-established cross-reactivity of oxytocin with vasopressin receptor subtypes (Jepsen et al., 2021), responsible of unwanted side effects (e.g., antidiuresis and local vasoconstriction at the site of application), the ability of peptide **50** to bind hV1aR and hV1bR subtypes was also positively evaluated, demonstrating a superior *in vitro* pharmacological profile compared to the native peptide, thus resulting inactive toward the hV1aR and hV1bR (EC_50_ > 1,000 nM). In C57BL/6 male mice, the lead compound showed increased plasma half-life (24.2 h), delayed onset (T_max_ = 7 h) and high maximal plasma concentration (C_max_ = 5,281 nM), confirming the better stability and injection site depot effect. Moreover, it resulted peripherally restricted, with brain-to-plasma ratio of 0.005 after 7 h, and undetectable brain exposure after 24 h. To evaluate its anorexigenic effects, measurements of acute food consumption were performed on C57BL/6 male mice. Following a single subcutaneous administration, treatment with **50** determined a delayed and long-lasting effect on food intake, with consequent reduction of body weight for 2 days, supporting the therapeutic impact of oxytocin-based peptides for chronic metabolic diseases, and posing the basis for future preclinical evaluation of the drug candidate **50**.

**FIGURE 34:**
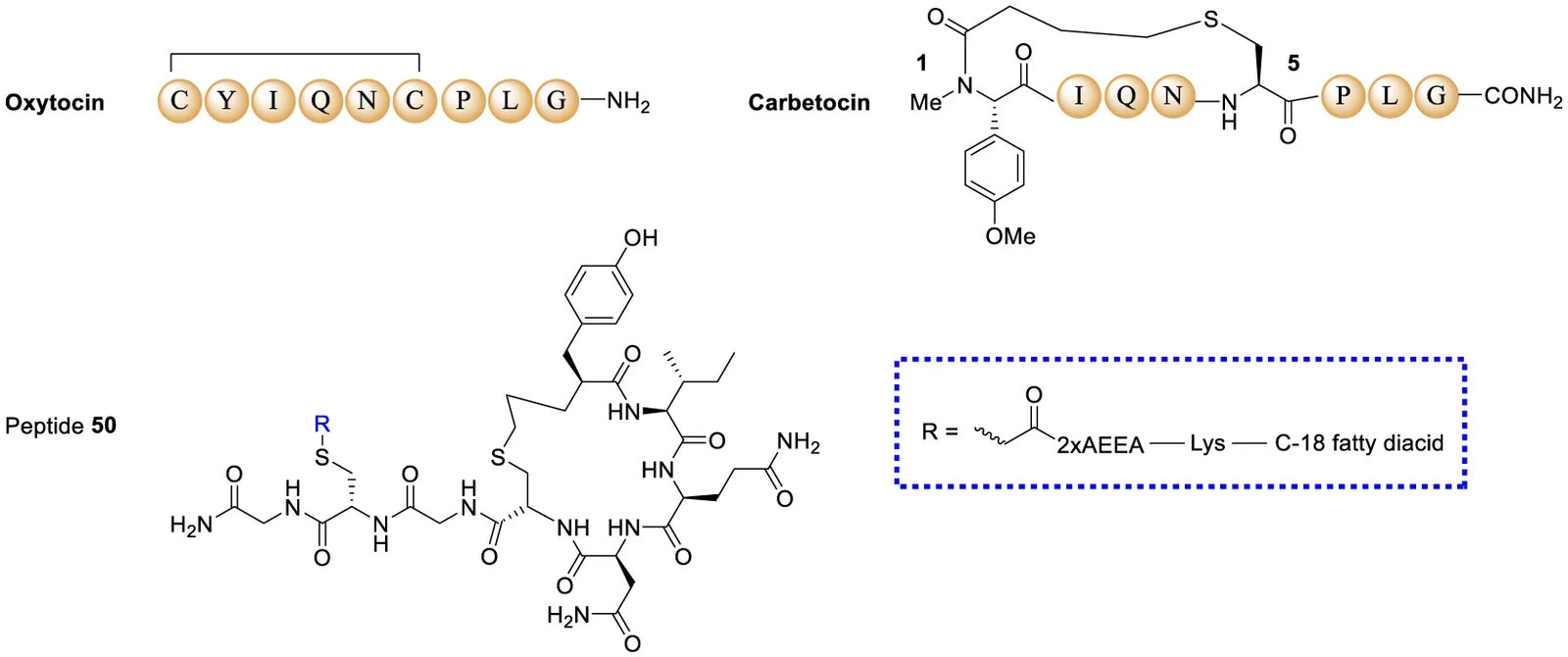
Amino acid sequence of oxytocin and its derivatives carbetocin and peptide **50**.

## 4 Conclusions and future perspective

The rapid growing obesity epidemic is worldwide the major driver of metabolic syndrome–related diseases, including T2D and NASH. GPCR drug discovery is currently experiencing a renewed surge of interest, catalyzed by major advances of the past couple of years in structural biology and elucidation of receptors dynamics. Peptides targeting GPCRs are cornerstones in treating several diseases due to their high specificity *in vivo*. This is evidenced by the great number of peptide-based therapeutics currently in clinical trials. Moreover, conjugation of peptides with small molecules is a valuable alternative to antibody-drug conjugates, enabling the delivery of the drug to the intended site of action. Novel technologies and the expansion of the repertoire of new targetable allosteric sites on GPCRs could help to address current limitations, opening new avenues in precision medicine for the treatment of metabolic disorders, inflammatory diseases, and neurological conditions. Indeed, clinical translation of GPCR-targeting peptides is expected to significantly benefit from strategies like adapting the treatments to individual genetic, environmental, and lifestyle profiles. For instance, obesity is a biologically complex and heterogeneous disease, and the common treatment options could be inadequate for some individuals, as demonstrated by the considerable proportion of patients not reaching sustained weight loss, or not having improvements in metabolic health. In fact, gene variants are able to modulate not only the receptor response to the natural ligands, but also to affect the drug efficacy. Common variants with modest impact on T2D risk include GIPR, MC4R, and the melatonin MT2 receptor. Moreover, some GLP-1R variants in the coding region were found to promote or protect against T2D development. Hence, the integration of pharmacogenomics, predictive biomarkers, and patient stratification may enable biomarker-guided therapeutic selection for individualized healthcare, ultimately improving the clinical outcomes while minimizing off-target effects. The sequencing of a growing number of human genomes to identify GPCR gene variants will facilitate to achieve this goal. Moreover, the ongoing progress in peptide engineering combined with advancement in structural biology and artificial intelligence-driven innovation could help to shape the development of next-generation GPCR-targeted therapies for diabetes, obesity, and other complex diseases.

In this comprehensive perspective, we summarized the most relevant medicinal chemistry campaigns toward GPCRs targeting peptides developed in the past 5 years to potentially treat metabolic disorders, highlighting how strategic amino acid mutations at the backbone of endogenous ligands helped to modulate their pharmacological profiles. We briefly discussed the rational use of non-natural amino acids and site-specific substitutions to enhance resistance to enzymatic degradation, particularly DPP-4-mediated N-terminal hydrolysis, which is one of the main factors limiting the therapeutic use of incretin-based peptides. Moreover, the insertion of fatty acid side chains through tailored linkers emerged as key strategy to promote reversible albumin binding, reduce renal clearance, and extend systemic exposure. We believe that this literature survey highlights opportunities for further exploration of GPCR targets and might be insightful for the development of next-generation peptide therapeutics to cure obesity and diabetes.

## Glossary

Aib: α-Aminoisobutyric acid
AMYR: Amylin receptor
BRET: Bioluminescence resonance energy transfer
B: Bullfrog
cAMP: Cyclic Adenosine Monophosphate
CRE-Luc: cAMP response element-driven -luciferase
CTR: Calcitonin Receptor
DACRAs: Dual amylin and calcitonin receptor agonists
FDA: Food and Drug Administration
Fc: Fragment crystallizable
GIP: Glucose-dependent Insulinotropic Polypeptide
GCGR: Glucagon receptor
GLP-1: Glucagon-like Peptide
GPCR: G-Protein Coupled Receptors
GDF-15: Growth differentiation factor 15
hSA: Human serum albumin
**K**_**D**_: Dissociation constant
MCR: Melanocortin receptor
NASH: Non-alcoholic steatohepatitis
NPY: Neuropeptide Y
OEG: Oligoethylene glycol
OXM: Oxyntomodulin
PP: Pancreatic polypeptide
PEG: Polyethylene glycol
PYY: Peptide YY
PD: Pharmacodynamic
PK: Pharmacokinetic
R: Receptor
r: Rodent
**T**_**max**_: Extended time required to reach maximum plasma concentration
**t**_**1/2**_: Half-life
T2D: Type-2 Diabetes Mellitus
X: *Xenopus*
